# The Multitarget Compound ZLY032 Achieves Treatment of Chronic Wounds

**DOI:** 10.1002/advs.202503098

**Published:** 2025-07-17

**Authors:** Manyu Gong, Zhiyuan Du, Tiantian Gong, Yu Wang, Chenchen Yin, Bosi Sun, Zijia Liu, Lianru Chen, Zibin Liao, Wenxin Wang, Tianjiao Zhao, Yifei Wang, Ying Dong, Kexin Wang, Mengru Ma, Weijun Li, Jiacheng Li, Haodong Li, Congcong Lin, Ying Zhang, Yu Liu, Xin Liu, Tao Ban, Hongxia Bao, Ying Zhang, Yang Zhang, Zheng Li, Lei Jiao

**Affiliations:** ^1^ Department of Medicinal Chemistry and Natural Medicine Chemistry Department of Pharmacology (State‐Province Key Laboratories of Biomedicine‐Pharmaceutics of China Key Laboratory of Cardiovascular Medicine Research, Ministry of Education) College of Pharmacy Harbin Medical University Harbin Heilongjiang 150081 P. R. China; ^2^ College of Bioinformatics Science and Technology Harbin Medical University Harbin Heilongjiang 150081 China; ^3^ School of Pharmacy Guangdong Pharmaceutical University Guangzhou 510006 P. R. China; ^4^ The Second Affiliated Hospital of Harbin Medical University Harbin Heilongjiang 150081 China; ^5^ Department of Pharmaceutics School of Pharmacy Harbin Medical University Harbin Heilongjiang 150081 China; ^6^ Genomics Research Center Key Laboratory of Gut Microbiota and Pharmacogenomics of Heilongjiang Province State‐Province Key Laboratory of Biomedicine‐Pharmaceutics of China College of Pharmacy Harbin Medical University Harbin Heilongjiang 150081 China

**Keywords:** antibacterial, anti‐inflammatory, argininosuccinate lyase, chronic wounds, ZLY032

## Abstract

The approach to dealing with chronic wounds is still challenging in clinic. Multitarget drugs have the advantages of superior therapeutic effect and minimal adverse reactions. The aim of the present study is to develop a multitarget treatment strategy for skin wound healing. It is showed here that topical application of ZLY032, a dual free fatty acid receptor 1 (FFA1)/peroxisome proliferator‐activated receptor (PPARδ) agonist, accelerates the healing of full‐thickness skin wounds in both normal mice and rabbits, even in the diabetic mice and methicillin‐resistant *Staphylococcus aureus* (MRSA) infected mice. At the eukaryotic cell level, ZLY032 shows the ability to accelerate angiogenesis and inhibit inflammation via targeting PPARδ and FFA1. At the prokaryotic cell level, ZLY032 exhibits antibacterial activity against *Staphylococcus aureus* and MRSA via targeting argininosuccinate lyase (ASAL, *argH*). At the application level, ZLY032‐loaded microneedles demonstrate a therapeutic effect on wound healing in rabbits with an improved dosing frequency. In this work, ZLY032 application is verified as a potential multitarget strategy for wound healing with good safety and efficacy. Furthermore, the discovery of the antimicrobial target of ZLY032 provides a theoretical basis for the development of novel antibiotics against drug‐resistant strains.

## Graphical abstract

ZLY032, a dual free fatty acid receptor 1/peroxisome proliferator‐activated receptor δ agonist, accelerates wound healing in normal/diabetic/methicillin‐resistant *Staphylococcus aureus*‐infected models by promoting angiogenesis, reducing inflammation, and exhibiting antibacterial activity via argininosuccinate lyase targeting, with demonstrated efficacy in microneedle delivery systems.

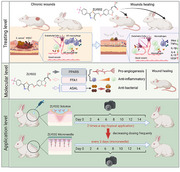

## 1 Introduction

The approach to dealing with large area burns, severe ulcers, surgical wounds, diabetic feet, and other chronic wounds is still challenging in the clinic.^[^ ^1^, ^2^ ^]^ Patients with chronic wounds or extensive trauma have heightened susceptibility to bacterial infection, which increases the difficulty of treatment.^[^ ^3^, ^4^ ^]^ Skin wound healing is a complex and dynamic biological process, which mainly consists of four stages, including hemostasis, inflammation (macrophage‐mediated accumulation of inflammatory factors), proliferation (angiogenesis mediated by endothelial cell proliferation and differentiation), and tissue remodeling (collagen fiber remodeling and extracellular matrix composition adjustment).^[^ ^5^, ^6^, ^7^, ^8^ ^]^ In view of the above wound healing process, growth factors (recombinant platelet‐derived growth factor (PDGF) and recombinant human epidermal growth factor (rEGF), which show proliferation and differentiation promoting effects, are currently used^[^ ^9^, ^10^, ^11^ ^]^ and antibiotics like penicillins (having antibacterial effects) and erythromycin (having antibacterial and anti‐inflammatory effects), were used for treating bacterial infected wound.^[^ ^12^, ^13^, ^14^ ^]^ Due to the complex process of wound healing, which is influenced by multiple factors.^[^ ^15^ ^]^ Multitarget drugs have advantages in treating chronic wounds.^[^ ^16^ ^]^ Therefore, developing multitarget treatment strategies to promote wound healing becomes a prioritized task of both basic researchers and clinicians.

Previously, ZLY032, a dual free fatty acid receptor 1 (FFA1/FFAR1/GPR40)/peroxisome proliferator‐activated receptor (PPARδ) agonist, was designed and synthesized which had a good regulatory effect on improving glucolipid metabolism and alleviating hepatic fibrosis with good pharmacokinetic characteristics.^[^ ^17^, ^18^ ^]^ In these studies, it was verified that ZLY032 had anti‐inflammatory activity in db/db mice. Additionally, it has been reported that FFAR1 agonists had anti‐inflammatory and antioxidant effects in addition to hypolipidemic and hypoglycemic action.^[^ ^17^ ^]^ Moreover, Araújo and co‐workers had demonstrated that FFA1 was expressed in the skin cells^[^ ^19^ ^]^ and that its synthetic selective agonist GW9508 could accelerate wound healing. PPARδ, the other target of ZLY032, is a ligand‐activated nuclear transcription factor belonging to the nuclear receptor superfamily and the main function of which is to inhibit the activation of NF‐κB during the inflammatory process.^[^ ^20^ ^]^ Furthermore, it has been reported that activation of PPARδ has a significant therapeutic effect on vascular homeostasis and promotes wound healing by regulating keratinocyte proliferation, migration, and adhesion.^[^ ^21^ ^]^ In a more recent study, a PPARβ/FFA1 dual agonist Y8 was reported to promote the healing of diabetic wounds and its therapeutic effect was better than that of single‐target agonists.^[^ ^22^ ^]^ These published results, prompted us to propose that ZLY032 might play a role in the promotion of wound healing via activating FFA1 and PPARδ, key targets in the treatment of skin wound healing.

Bacterial infections can trigger delayed wound healing and even more severe systemic damage.^[^ ^23^ ^]^ Recently, it has been established that *Staphylococcus*, *Proteus*, *Enterobacter*, *Helcococcus*, and *Pseudomonas* genera were strongly enriched in wound, compared with normal skin.^[^ ^24^ ^]^ Among them, *Staphylococcus aureus*, one of the most prevalent contributors to skin, could cause chronic wound infection and even threaten to life.^[^ ^24^, ^25^, ^26^ ^]^ Moreover, it has been reported that selective inhibition of endogenous *S. aureus* could promote multiple aspects of wound repair.^[^ ^26^ ^]^ Over the past few decades, the emergence and rapid spread of methicillin‐resistant *Staphylococcus aureus* (MRSA) has made the treatment of *S. aureus* infections increasingly challenging. Thus, identifying multitarget drugs that target both eukaryotic cells (macrophages for anti‐inflammation and endotheliocytes for angiogenesis) and prokaryotic cells (*S. aureus* or MRSA for anti‐infection) may offer a promising strategy for the treatment of chronic wounds. However, it remains unknown that whether ZLY032 has an antibacterial effect.

Herein, we investigated the role of ZLY032 in promoting wound healing in the normal mice wound model, rabbit wound model, diabetic mice wound model, and MRSA‐infected mouse wound model. At the eukaryotic level, we verified the function of ZLY032 on angiogenesis and inflammation. At the prokaryotic level, we investigated the inhibiting effects and target of ZLY032 on *S. aureus* and MRSA. Our findings will lay a scientific foundation for the development of multitarget drug for the treatment of chronic wounds and the development of novel antibiotics against drug‐resistant strains.

## 2 Results

### 2.1 ZLY032 Accelerates Wound Healing in Both Mouse and Rabbit Models

**Figure 1:**
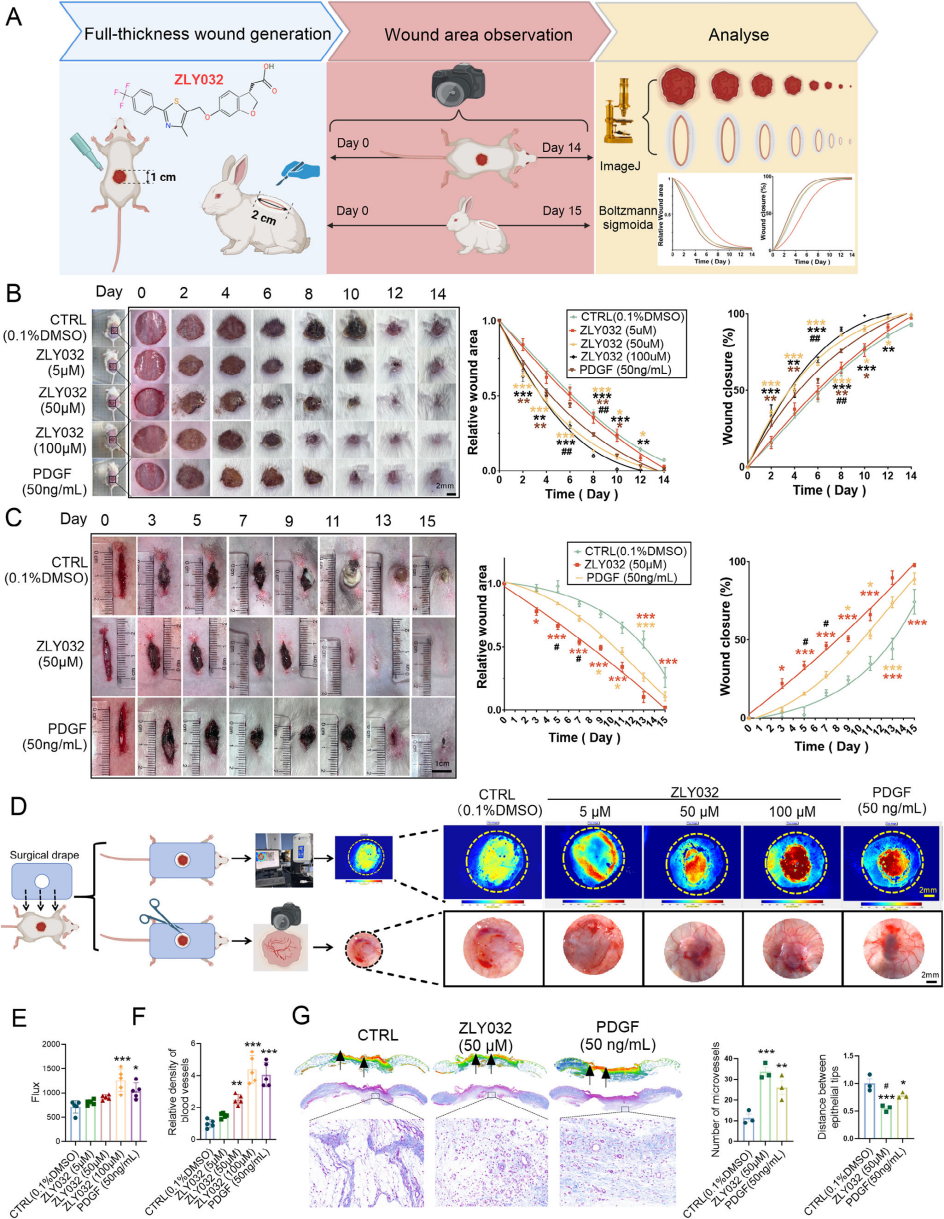
ZLY032 accelerates wound healing in both mouse and rabbit models. **A**) The structure of ZLY032, the wound generation and photo acquisition model diagram, wound size analysis flowchart. B) Left panel: representative photographs showing the time‐dependent closure of wounds in male mice and the wound healing‐promoting effect of ZLY032. PDGF was acted as positive control. Middle and right panel: the wound area and wound closure rate of each male mice at varying time points. *p < 0.05, **p < 0.01, ***p < 0.001 versus CTRL (0.1% DMSO); ^##^ *p* < 0.01 versus PDGF; *n* = 14 for each group. Scale bar: 2 mm. (Mean ± SD; two‐ way ANOVA followed by Tukey's multiple comparisons test among multiple groups.) C) Left panel: representative photographs showing the time‐dependent closure of wounds in rabbits. PDGF was acted as positive control. Middle and right panel: the wound area and wound closure rate of each rabbit at varying time points. *p < 0.05, ***p < 0.001 versus CTRL; ^#^ *p* < 0.05 versus PDGF; *n* = 6 for each group. (Mean ± SD; two‐ way ANOVA followed by Tukey's multiple comparisons test among multiple groups.) D–F) The blood flow at the wound site was detected by Doppler blood flow detector on day 8 after the administration of ZLY032 and PDGF. *p < 0.05, ***p < 0.001 versus CTRL, *n* = 5. (Mean ± SD; ordinary one‐way ANOVA followed by Tukey's multiple comparisons test among multiple groups.) G) Representative examples of Masson staining showing the effects of ZLY032 in microvessels and distance between epithelial tips (as indicated by the shortened distance between the black arrows) during the healing process (days 8). *p < 0.05, **p < 0.01, ***p < 0.001 versus CTRL; ^#^ *p* < 0.05 versus PDGF; *n* = 3. (Mean ± SD; ordinary one‐way ANOVA followed by Tukey's multiple comparisons test among multiple groups).

Prior to the in vivo experiment, we conducted a preliminary experiment at the in vitro level. As shown in Figure S1A–C of the Supporting Information, ZLY032 exhibited effects on promoting the proliferation of HUVECs compared with the control group at concentrations of 0.05, 0.1, 0.25, 0.5, 1, 2.5, 5, 25, 50, and 100 µm. Moreover, ZLY032 tremendously promoted the migration and tube formation of HUVECs at the concentrations of 0.5, 5, and 50 µm compared with the control group. The function of ZLY032 on HUVECs was similar to that of positive control VEGF (20 ng mL^−1^). Next, we verified the effects of ZLY032 on inflammatory responses. As demonstrated in Figure S1D of the Supporting Information, compared with nontreated control cells, the macrophages (RAW264.7 cells) treated with LPS had elevated levels of proinflammatory factors including iNos and Tnfα. This elevation was significantly rescued by ZLY032 in a dose‐dependent manner. Conversely, ZLY032 obviously increased the levels of anti‐inflammatory factors including IL‐10 and Vegfa in the LPS‐stimulated RAW264.7 cells. Peptidoglycan (PGN) from Gram‐positive bacteria has been used to treat macrophages to induce inflammatory reactions.^[^ ^27^, ^28^ ^]^ To further verify the effects of ZLY032 on inflammatory responses, we detected the effects of ZLY032 on the PGN‐treated RAW264.7 cells. The results in Figure S1D of the Supporting Information indicated that ZLY032 (5 and 50 µm) significantly decreased the expression of proinflammatory factors including iNos and Tnfɑ and significantly increased the expression of anti‐inflammatory factors including IL‐10 and Vegfa in PGN‐treated macrophages, while ZLY032 did not have the above‐mentioned effects on Tnfɑ and IL‐10 at a concentration of 0.5 µm. These data prompted us to explore the potential function of ZLY032 on the process of wound healing. We began with developing a wound on the dorsal skin of each mouse and rabbit and the wound areas in different groups were measured and photographed (**Figure** **1**A). As shown in Figure 1B, the wounds treated with ZLY032 (50 and 100 µm) exhibited remarkably accelerated wound closure relative to the control male mice (CTRL‐0.1% DMSO). In order to have a more straight forward view of wound closure, we constructed the curves with percent wound closure as a function of time. ZLY032 (50 and 100 µm) treated male mice had a significantly shortened healing time course with a time span for 50% wound closure (WCT50) of 4 days, compared to the control counterparts that had a WCT50 of 6 days. From day 2 to day 12, ZLY032 significantly promoted wound healing in male mice compared with the control group. Interestingly, on day 6 to day 8, the wound promotion effect of ZLY032 (50 and 100 µm) is better than that of PDGF, an FDA‐approved drug for the treatment of diabetic wound. ZLY032 had concentration‐dependent effects on the promotion of wound healing and it did not act at the concentration of 5 µm. In addition, we found that ZLY032 (50 and 100 µm) had no effect on heart weight and cardiac function, liver weight and ultrastructure, kidney weight and microstructure, lung weight, and spleen weight in male mice after 14 days of local administration (Figure S2, Supporting Information). Furthermore, we established a liquid chromatography‐mass spectrometry (LC‐MS) method to detect the residence of ZLY032 in the wound tissue and whether ZLY032 entered the blood circulation. ZLY032 (100 µm) was detectable in wound tissue homogenates at 0, 0.5, 1, 4, 8, and 12 h following topical administration to mice (Figure S3, Supporting Information), demonstrating its retention at the wound site. Moreover, blood samples were collected at 0.5, 1, 2, 4, and 8 h after ZLY032 (100 µm) treatment in the wound model of mice on days 0, 3, and 14. The results showed in Table S1 of the Supporting Information indicated that ZLY032 applied at the wound site did not enter the systemic circulation of mice. These results suggested that ZLY032 had good safety and effectiveness when used locally in the treatment of wounds. In addition, we investigated the effect of ZLY032 on wound healing in female mice. As shown in Figure S4 of the Supporting Information, similar to male mice, ZLY032 significantly promoted wound healing in female mice at a concentration of 100 µm, compared with the control group.

In order to further verify the effect of ZLY032 on wound healing, we established a rabbit wound model (Figure 1A). As illustrated in Figure 1C, topical application of ZLY032 (50 µm) significantly improved rabbit wound healing compared to the control group from day 3 onward. On days 5 to 13, ZLY032 (50 µm) was more effective in promoting wound healing than PDGF group. Furthermore, the WCT50 of ZLY032 (50 µm) was 8 days for rabbit wound, which was obviously shorter than that of control group (13 days).

To further explore the effect of ZLY032 on chronic wounds, we constructed a wound model in type I diabetic male mice (T1DM). As shown in Figure S5 of the Supporting Information, the wound healing ability of diabetic male mice was significantly weaker than that of nondiabetic male mice. Compared with T1DM group, ZLY032 can significantly promote wound healing of in diabetic mice at concentration of 50 and 100 µm. Moreover, ZLY032 (50 µm) exhibited similar improved effects on blood flow and vessel density at the wound site in diabetic mice (Figure S5E–G, Supporting Information). Since the regulatory effect of the local metabolic microenvironment (such as oxidative stress and advanced glycation end products) under hyperglycemic conditions can also determine the healing of diabetic wounds, therefore, we assessed oxidative stress markers and advanced glycation end products (AGEs) in wound tissues from control (normal wounds), diabetic mice, and diabetic mice treated with ZLY032 (100 µm) on days 3 and 8 postwounding. As shown in Figure S6A,B of the Supporting Information, ZLY032 administration significantly reduced malondialdehyde (MDA) levels and significantly elevated superoxide dismutase (SOD) levels in diabetic wound tissues at both 3 and 8 days, compared to the untreated diabetic wound group. Reactive oxygen species (ROS) staining of wound sections (Figure S6C,D, Supporting Information) demonstrated elevated ROS levels in diabetic mouse wounds. This increase was significantly attenuated by ZLY032 treatment. Furthermore, AGEs levels in wound tissues, evaluated by immunohistochemistry and enzyme‐linked immunosorbent assay (ELISA) assays, were significantly reduced in ZLY032‐treated diabetic mice compared to untreated diabetic controls (Figure S6E,F, Supporting Information). In vitro, we detected the effects of ZLY032 on high glucose‐treated HUVECs and macrophages (RAW264.7), respectively. The results in Figure S7 of the Supporting Information showed that compared with the control group (5.5 mm glucose), high glucose (30 mm glucose) inhibited the proliferation, migration and tube formation of HUVECs, but ZLY032 could significantly improve this situation. Moreover, ZLY032 significantly inhibited the levels of iNos, Tnfα, IL‐1β, and IL‐6 in high glucose‐treated RAW264.7 cells, and significantly increased the level of Vegfa (Figure S8A–F, Supporting Information). Flow cytometry results showed that ZLY032 significantly reduced the level of CD86^+^ cells and increased the level of CD206^+^ cells in high glucose‐induced RAW264.7 cells (Figure S8G,H, Supporting Information). These results indicate that ZLY032 ameliorates the metabolic microenvironment and promotes the diabetic wounds healing.

The above data indicated that ZLY032 could accelerate wound healing in normal mice, T1DM mice, and rabbits.

### 2.2 ZLY032 Promotes Angiogenesis and Inhibits Inflammation by Targeting PPARδ and FFA1

Previous in vitro experiments demonstrated that ZLY032 promotes tube formation in HUVECs and modulates the expression of inflammation‐related factors in RAW264.7 cells. These findings suggest that ZLY032 may facilitate angiogenesis and exert anti‐inflammatory effects at wound sites. To further clarify this issue, we first evaluated the effects of ZLY032 on the vessel density at the mouse wound area. As shown in Figure 1D,F, ZLY032 significantly increased vessel density in the mouse wound area at concentration of 50 and 100 µm, compared with the control group. By using Doppler perfusion imaging, we verified that ZLY032 (50 and 100 µm) could obviously increase blood flow flux at the wound site of normal mice compared with the control group (Figure 1D,E). Consistently, Masson staining in wound sites demonstrated that the number of microvessels was increased and the distance between epithelial tips was decreased after receiving topical ZLY032 (50 µm) for 8 days in the wound area (Figure 1G). The above in vivo data, combined with the data in Figure S1A–C of the Supporting Information indicated that ZLY032 have effects on promoting angiogenesis.

The ongoing inflammatory response is another key factor hindering wound healing. The accumulation of a large number of proinflammatory factors at the wound area will result in a chronic wound.^[^ ^29^, ^30^, ^31^ ^]^ We next tried to explore the effect of ZLY032 on inflammation. First, the expression level of proinflammatory cytokines including IL‐1β and IL‐6 was detected in the wound tissue. The data in Figure S9A–C of the Supporting Information showed that the expression levels of IL‐1β and IL‐6 at the wound site of mice were significantly reduced on the 3rd and 5th day after ZLY032 application, compared with the control group. It is well‏ ‏established that macrophages are key regulators of wound healing, which can regulate the release of inflammatory cytokines and promote tissue repair.^[^ ^32^ ^]^ The generation of reparative macrophages can promote the repair of tissue damage^[^ ^33^ ^]^ and CD206 is a representative marker of reparative macrophages. To evaluate the effect of ZLY032 on the generation of reparative macrophages, we evaluated the amount of reparative macrophages at the wound site by flow cytometry. Results in Figure S9D,E of the Supporting Information showed that the number of CD11b^+^ CD86^+^ macrophages (proinflammatory type) was decreased at the wound site of mice after ZLY032 administration for 3 days. While the number of CD11b^+^ CD206^+^ macrophages (reparative type of macrophages) at the wound site of mice after ZLY032 administration for 7 days was increased significantly, compared with the control group. These in vivo data, combined with the data from Figure S1D of the Supporting Information, indicate that ZLY032 can promote wound healing by exerting anti‐inflammatory effects.

In addition, the compound ZLY032 exhibited a significant inhibitory effect on the inflammation and pain‐associated protein COX‐2 at a concentration of 5 µm (Figure S5H, Supporting Information). Furthermore, we performed coculture cell experiments to further confirm whether ZLY032 indirectly promotes angiogenesis by modulating macrophages. Macrophages (RAW264.7 cells) treated with ZLY032 (0, 0.5, 5, 50 µm) were cocultured with HUVECs and the proliferation, migration, and tube formation of HUVECs were assessed. The results in (Figure S10, Supporting Information) showed that HUVECs cocultured with RAW264.7 cells, which was pretreated with ZLY032 at concentrations of 0, 0.5, 5, and 50 µm, had dose‐dependent promotion effects on proliferation, migration, and tube formation, compared with the un‐co‐cultured HUVECs. The above data indicated that ZLY032 can accelerate wound healing by promoting angiogenesis and inhibiting inflammatory responses.

Next, we performed a series of experiments to verify the molecular mechanisms of ZLY032 in neovascularization and anti‐inflammation. Previously, it was demonstrated that ZLY032 was a dual agonist for FFA1 (EC50: 68 nm) and PPARδ (EC50: 102 nm) and fitted well with the cocrystal structure of PPARδ (PDB number: 1GWX) and FFA1 (4PHU) in binding site by docking study.^[^ ^18^ ^]^ To further illustrate that ZLY032 targets PPARδ and FFA1, the cellular thermal shift assay (CETSA) was performed and the results showed that ZLY032 had direct binding ability with FFA1 and PPARδ (Figure S11, Supporting Information). To prove that ZLY032 exerts proangiogenic and anti‐inflammatory effects by activating PPARδ and FFA1, we took the advantage of PPARδ inhibitor GSK3787 and FFA1 inhibitor DC26016 as pharmacological tools in the subsequent experiments. We demonstrated that the addition of GSK3787 (0.1 µm) or DC26016 (10 µm) separately, or the addition of GSK3787 and DC26016 together could significantly inhibit the proliferation, migration, and tube formation in HUVECs and showed no significant effects on the expression of iNos, Tnfα, IL‐10, and Vegfa (Figure S12, Supporting Information). Moreover, it was verified that ZLY032 lost its effects on promoting proliferation, migration and tube formation in HUVECs pretreated with GSK3787 (0.1 µm) or DC26016 (10 µm) or GSK3787 and DC26016 together (Figure S13A–C, Supporting Information). Moreover, inhibition of PPARδ or FFA1 with GSK3787 or DC26016 or GSK3787 and DC26016 together significantly weakened the anti‐inflammatory effects of ZLY032 in RAW264.7 cells (Figure S13D–G, Supporting Information). Moreover, ZLY032 significantly increased the expression level of CD206, Arg1, Tgfβ1, and Fizz1 in RAW264.7 cells, while inhibition of PPARδ or FFA1 with GSK3787 or DC26016 or GSK3787 and DC26016 together significantly inhibited these effects of ZLY032 (Figure S13H–K, Supporting Information).

**Figure 2:**
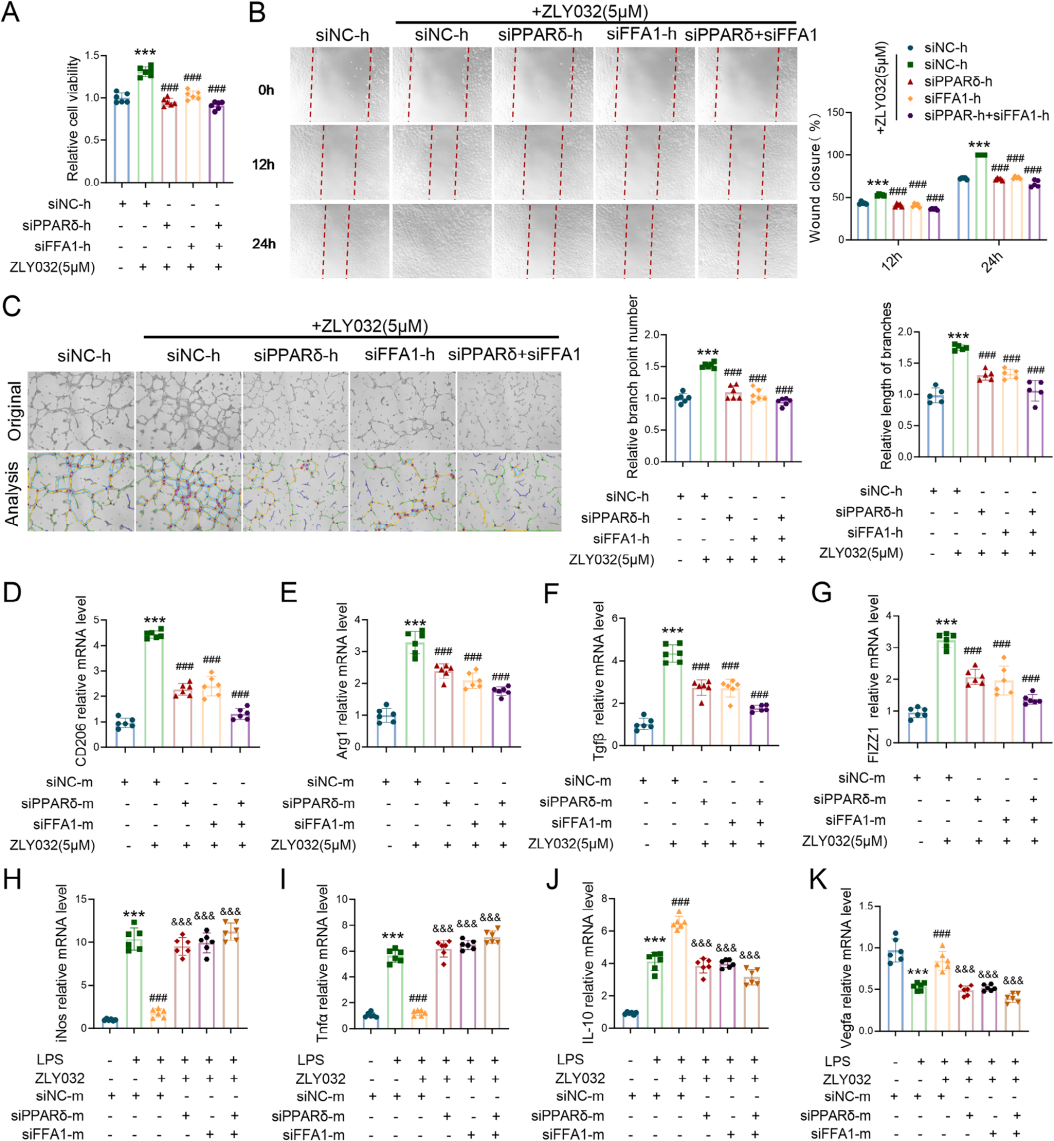
ZLY032 promotes angiogenesis and exerts anti‐inflammatory effects. A) Effect of ZLY032 (5 µm) combined with single or dual PPARδ/FFA1 knockdown on HUVECs activity as assessed by CCK‐8 assay. ***p < 0.001 versus siNC‐h, ^###^ *p* < 0.001 versus siNC‐h +ZLY032 (5 µm); *n* = 6 for each group. (Mean ± SD; ordinary one‐way ANOVA followed by Tukey's multiple comparisons test among multiple groups.) B) Effect of ZLY032 (5 µm) combined with single or dual PPARδ/FFA1 knockdown on HUVECs migratory capacity as assessed by scratch assay. ***p < 0.001 versus siNC‐h; ^###^ *p* < 0.001 versus siNC‐h+ZLY032 (5 µm); *n* = 5 for each group. (Mean ± SD; two‐way ANOVA followed by Tukey's multiple comparisons test among multiple groups.) C) Effect of ZLY032 (5 µm) combined with single or dual PPARδ/FFA1 knockdown on HUVECs tube formation capacity as assessed by tube formation assay. ***p < 0.001 versus siNC‐h; ^###^ *p* < 0.001 versus siNC‐h+ZLY032(5 µm); *n* = 6 for each group. (Mean ± SD; ordinary one‐way ANOVA followed by Tukey's multiple comparisons test among multiple groups.) D–G) qRT‐PCR to detect the effects on the expression of CD206, Arg1, Tgfβ1, and Fizz1 in the ZLY032 (5 µm) combined with single or dual PPARδ/FFA1 knockdown in RAW264.7 cells. ***p < 0.001 versus siNC‐m; ^###^ *p* < 0.001 versus siNC‐m+ZLY032 (5 µm); *n* = 6 for each group. (Mean ± SD; ordinary one‐way ANOVA followed by Tukey's multiple comparisons test among multiple groups.) H–K) qRT‐PCR was used to detect the effects of ZLY032 (5 µm) in combination with single or combined knockdown of *PPARδ* and *FFA1* on the expression of iNos, Tnfα, IL‐10, and Vegfa in LPS stimulated RAW264.7 cells. ***p < 0.001 versus siNC‐m; ^###^ *p* < 0.001 versus LPS+siNC‐m; ^&&^ *p* < 0.01, ^&&&^ *p* < 0.001 versus LPS+siNC‐m+ZLY032 (5 µm); *n* = 6 for each group. (Mean ± SD; ordinary one‐way ANOVA followed by Tukey's multiple comparisons test among multiple groups).

**Figure 3:**
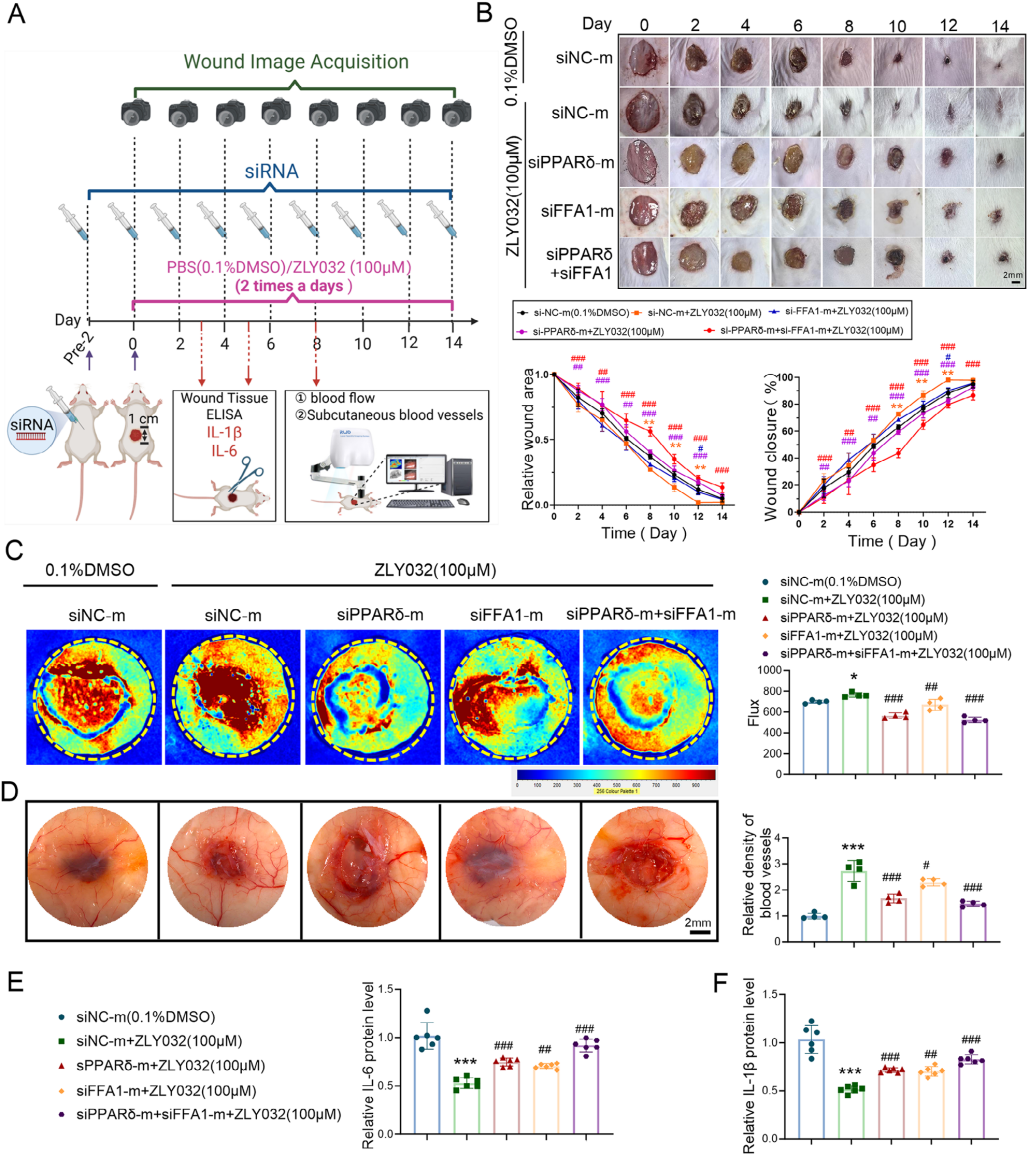
ZLY032 promotes angiogenesis and exerts anti‐inflammatory effects by activating PPARδ and FFA1. A) The wound generation and photo acquisition model diagram and wound size analysis flowchart. B) Up panel: representative photographs showing the time‐dependent closure of wounds in male mice and the wound healing‐promoting effect of ZLY032 combined with single or dual PPARδ/FFA1 knockdown. Down panel: the wound area and wound closure rate of each male mice at varying time points. **p < 0.01 versus siNC‐m (0.1% DMSO); ^#^ *p* < 0.05, ^##^ *p* < 0.01, ^###^ *p* < 0.001 versus siNC‐m+ZLY032 (100 µm); *n* = 4 for each group. Scale bar: 2 mm. (Mean ± SD; two way ANOVA followed by Tukey's multiple comparisons test among multiple groups.) C,D) The blood flow at the wound site was detected by Doppler blood flow detector on day 8 after the administration of ZLY032 combined with single or dual PPARδ/FFA1 knockdown. *p < 0.05, ***p < 0.001 versus siNC‐m (0.1% DMSO), *n* = 4. (Mean ± SD; ordinary one‐way ANOVA followed by Tukey's multiple comparisons test among multiple groups.) E,F) ELISA kits to detect the expression level of IL‐6 and IL‐1β in the wound tissue on the 3rd day of ZLY032 (100 µm) administration combined with single or dual PPARδ/FFA1 knockdown. ***p < 0.001 versus siNC‐m (0.1% DMSO); ^##^ *p* < 0.01, ^###^ *p* < 0.001 versus siNC‐m (0.1% DMSO)+ZLY032 (100 µm); *n* = 6. (Mean ± SD; ordinary one‐way ANOVA followed by Tukey's multiple comparisons test among multiple groups).

To further demonstrate the dependence of ZLY032 on PPARδ and FFA1, we designed species‐specific small interfering RNAs (siRNAs) targeting human (h) and murine (m) PPARδ and FFA1, alongside corresponding nontargeting controls (siNC‐h, siNC‐m, siPPARδ‐h, siPPARδ‐m, siFFA1‐h, siFFA1‐m). Knockdown efficiency was validated in HUVECs and RAW264.7 cells (Figure S14A,B, Supporting Information). Compared with siNC‐h controls, single knockdown of either PPARδ or FFA1, as well as dual knockdown of both targets, significantly attenuated HUVEC proliferation, migration, and tube formation (Figure S14C–E, Supporting Information). Critically, relative to the siNC‐h + ZLY032 (5 µm) group, single or dual knockdown of PPARδ and/or FFA1 abolished the proangiogenic effects of ZLY032, significantly reducing proliferation, migration, and tube formation capacity (**Figure** **2**A–C). Furthermore, compared with the siNC‐m + ZLY032 (5 µm) group, single or dual knockdown of PPARδ and/or FFA1 significantly reduced ZLY032‐induced expression of M2‐associated markers (CD206, Arg1, Tgfβ1, Fizz1) in RAW264.7 cells (Figure 2D–G). In LPS‐stimulated RAW264.7 cells, single or dual knockdown of PPARδ and/or FFA1 significantly diminished the effect of ZLY032 (5 µm) anti‐inflammatory relative to the siNC‐m + ZLY032 group. This was evidenced by significantly elevated expression of inflammatory markers (iNos, Tnfα) and significantly reduced expression of anti‐inflammatory markers (IL‐10, Vegfa) (Figure 2H–K). At the in vivo level, we established mice model with reduced PPARδ and FFA1 at the wound site (**Figure** **3**A; Figure S14F, Supporting Information). As shown in Figure 3B, compared to the siNC‐m + ZLY032 (100 µm) group, individual knockdown of either PPARδ or FFA1, as well as dual knockdown of both targets, significantly attenuated the wound‐healing efficacy of ZLY032. Laser Doppler imaging on day 8 revealed significantly reduced wound perfusion and vascular density in all knockdown groups relative to siNC‐m + ZLY032 controls (Figure 3C,D). ELISA quantification of wound homogenates demonstrated that single or dual knockdown of PPARδ and/or FFA1 significantly elevated proinflammatory cytokines IL‐1β and IL‐6 levels at day 3 postwounding compared to the siNC‐m + ZLY032 group (Figure 3E,F). The above results indicated that ZLY032 exerted proangiogenic and anti‐inflammatory effects in eukaryotic cells by activating of PPARδ and FFA1.

Next, we further explored the downstream signals of ZLY032 lead to angiogenesis or inhibition of inflammation. Previous studies have shown that activating FFA1 or PPARδ can inhibit the NF‐κB signaling pathway, thereby inhibiting the inflammatory response.^[^ ^34^, ^35^ ^]^ To further verify whether ZLY032 inhibits inflammatory responses through the FFA1/PPARδ/NF‐κB pathway, we examined the expression level of pNF‐κB p65 and NF‐κB p65 in ZLY032‐treated RAW264.7 cells. The results in Figure S15A of the Supporting Information showed that ZLY032 significantly inhibited the expression of pNF‐κB p65 in LPS‐treated RAW264.7 cells. In addition, previous studies have shown that PPARδ can promote tubular formation and migration of endothelial cells by mediating VEGF secretion,^[^ ^36^ ^]^ and FFA1 knockdown can significantly inhibit the expression level of VEGF in endothelial cells.^[^ ^37^ ^]^ This suggests that ZLY032 may promote angiogenesis through the PPARδ/FFA1/VEGF pathway. Therefore, we used an ELISA kit to detect the effect of ZLY032 on the expression of VEGFa in HUVECs. Results in Figure S15B of the Supporting Information showed that ZLY032 increased the level of VEGFa in HUVECs compared with the control group. At the in vivo level, topical administration of ZLY032 (100 µm) to murine wounds significantly reduced the expression level of pNF‐κB p65 and markedly increased VEGF levels in wound tissues (Figure S15C, Supporting Information). Therefore, ZLY032 can exhibit anti‐inflammatory effect via regulating FFA1/PPARδ/NF‐κB pathway, and promote angiogenesis by promoting FFA1/PPARδ/VEGF pathway.

In addition to macrophages and endothelial cells, adipocytes are also involved in the wound healing process. Adipocytes can regulate the recruitment of fibroblasts to affect wound healing and can also participate in wound repair by secreting various inflammatory related factors such as IL‐6, IL‐1β, and growth factors.^[^ ^38^, ^39^, ^40^ ^]^ Moreover, Igf1r and Adipor1 were identified as receptors in adipocytes mediating wound healing.^[^ ^41^, ^42^ ^]^ Therefore, we evaluated the effects of ZLY032 on the expression of Fgf, Igf1r, Adipor1, IL‐1β, and IL‐6 in induced 3T3‐L1 cells. Results shown in Figure S16 of the Supporting Information demonstrated that ZLY032 (5 µm) did not affect the expression levels of Igf1r and Adipor1 compared with the control group. However, it promoted the expression of Fgf and inhibited the expression of inflammation‐related factors, including IL‐1β and IL‐6. Moreover, compared with the high glucose group, ZLY032 inhibited the expression of IL‐6 and IL‐1β and increased the expression of Fgf in induced 3T3‐L1 cells (Figure S17, Supporting Information). Combined knockdown of PPARδ and FFA1 attenuated ZLY032's regulatory effects on IL‐6, IL‐1β, and Fgf expression in induced 3T3‐L1 cells (Figure S18, Supporting Information). Furthermore, we also tested the effect of ZLY032 on epithelial cells (NCM460 cells). As shown in Figure S19 of the Supporting Information, ZLY032 promoted the proliferation and migration of NCM460 cells at the concentration of 0.5, 5, and 50 µm compared with the control group. In addition, compared with the high glucose group, ZLY032 enhanced the proliferation and migration of high glucose‐induced NCM460 cells (Figure S20, Supporting Information). Furthermore, single or dual knockdown of PPARδ and/or FFA1 significantly impaired basal migration compared to siNC‐h controls in NCM460 cells (Figure S21A, Supporting Information). And the enhancement of migration by ZLY032 (5 µm) was significantly blunted in NCM460 cells subjected to single or dual knockdown relative to the siNC‐h + ZLY032 group (Figure S21B, Supporting Information). The above data indicated that ZLY032 simultaneously targeting PPARδ and FFA1 can also regulate inflammatory factors in adipocytes while enhancing epithelial cell migration, despite its regulatory effect on endothelial cells and macrophages.

### 2.3 ZLY032 Exhibits Potent Antibacterial Activity against *S. aureus* and MRSA

**Figure 4:**
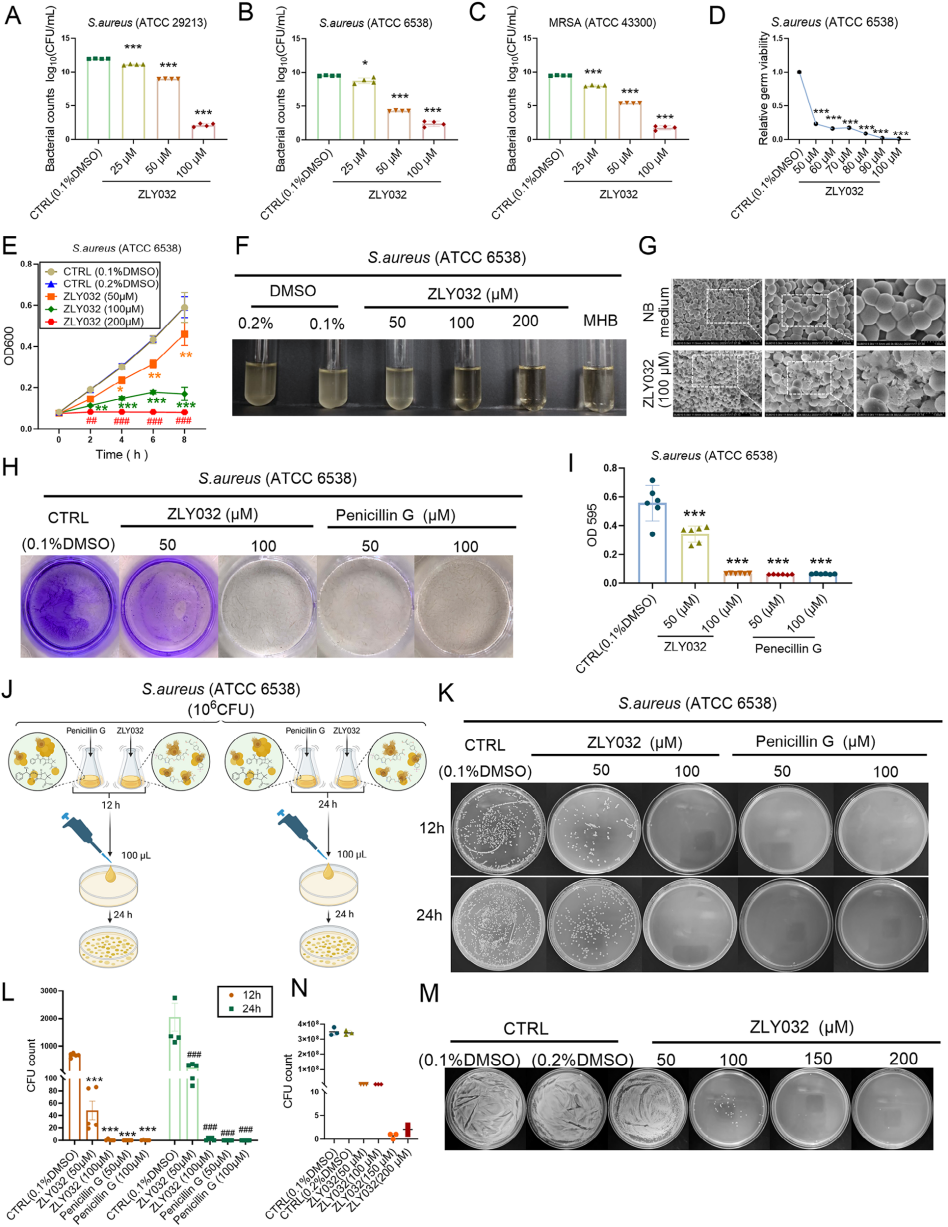
ZLY032 exhibits antimicrobial activities against *S. aureus*. A–C) The inhibiting effects of ZLY032 on *S. aureus* (ATCC 29213, ATCC 6538) and MRSA (ATCC 43300) at the concentrations of 25, 50, and 100 µm. *p < 0.05 versus CTRL (0.1% DMSO), ***p < 0.001 versus CTRL, *n* = 4. (Mean ± SD; ordinary one‐way ANOVA followed by Tukey's multiple comparisons test among multiple groups.) D) To explicit the minimum inhibitory concentration (MIC) of ZLY032 on *S. aureus* (ATCC 6538). ***p < 0.001 versus CTRL, *n* = 6. (Mean ± SD; ordinary one‐way ANOVA followed by Tukey's multiple comparisons test among multiple groups.) E) Time dependent inhibiting of *S. aureus* (ATCC 6538) by ZLY032 at 50, 100, and 200 µm. *p < 0.05, **p < 0.01, ***p < 0.001 versus CTRL (0.1% DMSO), ^##^ *p* < 0.01, ^###^ *p* < 0.001 versus CTRL (0.2% DMSO), *n* = 5. (Mean ± SD; two‐way ANOVA followed by Tukey's multiple comparisons test among multiple groups.) F) In vitro clarification assay to verify the inhibitory effect of ZLY032 on *S. aureus* (ATCC 6538) at the concentrations of 50, 100, and 200 µm. G) Using scanning electron microscope to demonstrate the damage effect of ZLY032 on *S. aureus* (ATCC 6538). H,I) The effect of ZLY032 on the activity of *S. aureus* (ATCC 6538) was determined by crystal violet staining. ***p < 0.001 versus CTRL (0.1% DMSO); *n* = 6 for each group. (Mean ± SD; ordinary one‐way ANOVA followed by Tukey's multiple comparisons test among multiple groups.) J) Experiment flowchart of ZLY032 against *S. aureus* (ATCC 6538). K,L) The effects of ZLY032 on the colony formation of *S. aureus* (ATCC 6538) after treating by ZLY032 (50 and 100 µm) or Penicillin G (50 and 100 µm) for 12 and 24 h. ***p < 0.001 versus CTRL (0.1% DMSO)‐12 h, ^###^ *p* < 0.001 versus CTRL (0.1% DMSO)‐24 h; *n* = 5 for each group. (Mean ± SD; two‐way ANOVA followed by Tukey's multiple comparisons test among multiple groups.) M,N) Determination of the MBC of ZLY032 against *S. aureus* (ATCC 6538) by colony formation assay. *n* = 3. (Mean ± SD; ordinary one‐way ANOVA followed by Tukey's multiple comparisons test among multiple groups).

Wound infection caused by bacterial overgrowth at the wound site is another key factor causing chronic wounds.^[^ ^43^, ^44^ ^]^ In an accidental cell contamination event, we were surprised to find that the HUVECs administered with ZLY032 (5 and 50 µm) were not affected, while the HUVECs treating without ZLY032 were all being contaminated, which aroused our great interest. We hypothesized that ZLY032 might have a potential antibacterial effect. To clarify this issue, we firstly evaluated the antibacterial effects of ZLY032 on Gram‐positive bacteria and Gram‐negative bacteria. Remarkably, ZLY032 showed concentration‐dependent antibacterial activity against *S. aureus* (ATCC 29213, ATCC 6538), and MRSA (ATCC 43300) compared with the control group and the inhibition rate is higher than 90% at the concentration of 100 µm (**Figure** **4**A–C; Figure S22A–C, Supporting Information). On the contrary, ZLY032 did not exhibit antibacterial activity against *Escherichia coli* at concentrations of 6.25, 12.5, 25, 50, and 100 µm (Figure S22D, Supporting Information). Moreover, the inhibition of ZLY032 against *Salmonella* is less than 50% at the concentrations of 16, 32, 64, and 128 µm (Figure S22E, Supporting Information) and the inhibition of ZLY032 against *Pseudomonas aeruginosa* and *Enterococcus faecalis* is less than 50% at concentrations of 6.25, 12.5, 25, 50, and 100 µm (Figure S22F,G, Supporting Information). We further tested the growth inhibitory function of ZLY032 on *S. aureus*. First, the minimal inhibitory concentration (MIC) of ZLY032 against *S. aureus* (ATCC6538) was determined as 100 µm (Figure 4D). Second, we monitored the bacterial viability of *S. aureus* (ATCC6538) exposed to various concentrations of ZLY032 at different times. The time–concentration inhibiting curve in Figure 4E showed that, compared with the control group, ZLY032 could inhibit the proliferation of most *S. aureus* (ATCC6538) from 2 to 8 h at 100 and 200 µm. In tube clarification test, ZLY032 completely inhibited the growth of *S. aureus* (ATCC6538) compared with the control group (Figure 4F). From the microscopic view, we clarified that ZLY032 (100 µm) destroyed membrane integrity of *S. aureus* and showed partial killing effects under scanning electron microscopy (Figure 4G).

To further clarify the anti‐*S. aureus* effect of ZLY032, we compared the antibacterial ability of ZLY032 with that of Penicillin G. By crystal violet staining, we confirmed that both ZLY032 (50 and 100 µm) and Penicillin G (50 and 100 µm) treated *S. aureus* (ATCC6538) had significantly decreased biofilm content compared with the control group (Figure 4H,I). Additionally, colony formation experiments demonstrated that both ZLY032 (100 µm) and Penicillin G (50 and 100 µm) had good inhibition effects against *S. aureus* (ATCC6538) for 12 and 24 h (Figure 4J–L). To further explore the bactericidal activity of ZLY032 against *S. aureus* (ATCC6538), we performed the minimum bactericidal concentration (MBC) determination experiment. As shown in Figure 4M,N, the MBC of ZLY032 against *S. aureus* (ATCC6538) was 150 µm.

**Figure 5:**
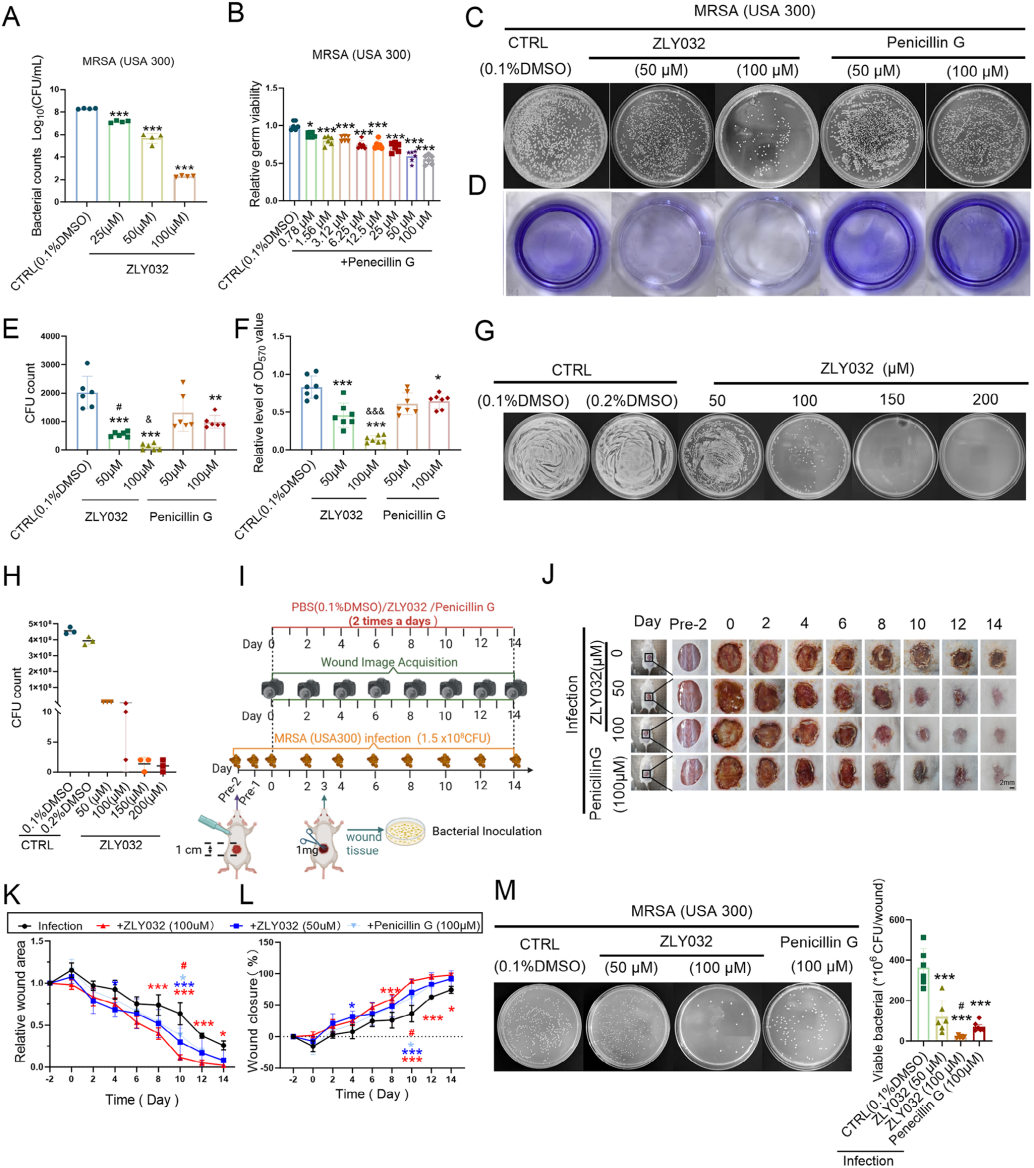
ZLY032 prompted MRSA‐infected wound healing. A) The inhibiting effects of ZLY032 on MRSA (USA300). ***p < 0.001 versus CTRL (0.1% DMSO); *n* = 4 for each group. (Mean ± SD; ordinary one‐way ANOVA followed by Tukey's multiple comparisons test among multiple groups.) B) The effects of Penicillin G on MRSA (USA300). *p < 0.05, ***p < 0.001 versus CTRL (0.1% DMSO), *n* = 6. (Mean ± SD; ordinary one‐way ANOVA followed by Tukey's multiple comparisons test among multiple groups.) C,E) The effect of ZLY032 on colony formation of MRSA (USA300) after treatment with ZLY032 (50 and 100 µm) or Penicillin G (50 and 100 µm) for 12 h. **p < 0.01, ***p < 0.001 versus CTRL (0.1% DMSO). ^#^ *p* < 0.05 versus Penicillin G (50 µm), ^&^ *p* < 0.05 versus Penicillin G (100 µm); *n* = 6 for each group. (Mean ± SD; ordinary one‐way ANOVA followed by Tukey's multiple comparisons test among multiple groups.) D,F) The effect of ZLY032 and Penicillin G on the activity of MRSA (USA300) was determined by crystal violet staining. *p < 0.05, ***p < 0.001, versus CTRL (0.1% DMSO), ^&&&^ *p* < 0.001 versus Penicillin G (100 µm); *n* = 7 for each group. (Mean ± SD; ordinary one‐way ANOVA followed by Tukey's multiple comparisons test among multiple groups.) G,H) Determine the MBC of ZLY032 against MRSA (USA300) by colony formation assay. *n* = 3. I) Flowchart of MRSA infected wound model in male mice. (Mean ± SD; ordinary one‐way ANOVA followed by Tukey's multiple comparisons test among multiple groups.) J–L) The wound healing‐promoting effect of ZLY032 MRSA infected wound model in mice. Penicillin G was acted as positive control. *p < 0.05, ***p < 0.001 versus infection; ^#^ *p* < 0.05 versus Penicillin G; *n* = 5 for each group. Scale bar: 2 mm. (Mean ± SD; two‐way ANOVA followed by Tukey's multiple comparisons test among multiple groups.) M) Representative images of bacterial colony in wound fluid and the amount of MRSA in the wound tissue on day 3. ***p < 0.001 versus CTRL (0.1% DMSO); ^#^ *p* < 0.05 versus Penicillin G (100 µm); *n* = 6 for each group. (Mean ± SD; ordinary one‐way ANOVA followed by Tukey's multiple comparisons test among multiple groups).

MRSA has become one of the significant pathogens in hospital and community infections, with a mortality rate of up to 63.1%.^[^ ^45^ ^]^ The data in Figure 4C indicated that ZLY032 had an inhibitory effect against MRSA (ATCC43300). Thus, we further explored the antibacterial action of ZLY032 against different strains of MRSA at different concentrations and using Penicillin G as a positive control. The results shown in **Figures** **5**A and S23 (Supporting Information) indicated that ZLY032 had significant inhibitory effects on MRSA (USA300). The MIC of ZLY032 against MRSA (USA300) was determined as 100 µm, which was same with the MIC of ZLY032 against *S. aureus* (ATCC6538) (Figure S23). The MIC of Penicillin G against MRSA (USA300) was much higher than 100 µm (Figure 5B). Furthermore, Penicillin G did not reduce the biofilm content of MRSA (USA300) or inhibit its proliferation at concentrations of 50 µm. While ZLY032 had a better inhibitory effect on the biofilm content and proliferation of MRSA (USA300) than Penicillin G at the concentration of 100 µm (Figure 5C–F). In addition, by using LIVE/DEAD assay, we demonstrated that ZLY032 (50 and 100 µm) did not produce significant bactericidal effect against MRSA (USA300) (no significant increase in red color) after 2 h of treatment, compared with the control group. And ZLY032 (50 and 100 µm) produced significant inhibitory effects against MRSA (USA300) (significant decrease in green color) after 12 h of treatment (Figure S24, Supporting Information). To further explore the bactericidal effect of ZLY032, we detected the MBC of ZLY032 against MRSA (USA300). The data shown in Figure 5G,H demonstrated that the MBC of ZLY032 against MRSA (USA300) was 150 µm. Next, we compared the inhibitory effect of ZLY032 with commonly used antibiotics including vancomycin and linezolid on MRSA (USA300) at the concentrations of 0.01, 0.05, 0.1, 0.5, 1, 5, 10, 50, and 100 µm. As showed in Figure S25 of the Supporting Information, ZLY032 started to take effect at concentration of 10 µm and completely inhibited MRSA proliferation at 100 µm. Vancomycin and linezolid started to take effect at 0.01 µm. Vancomycin completely inhibited MRSA proliferation at 0.5 µm and linezolid did so at 5 µm. The above data indicated that ZLY032 displays potent antibacterial activity against both *S. aureus* and MRSA.

### 2.4 ZLY032 Accelerates MRSA‐Infected Wound Healing

To further clarify whether ZLY032 promotes healing of wounds infected with MRSA (USA300), we constructed a mouse wound model infected with MRSA (Figure 5I). As illustrated in Figure 5J–L, ZLY032 (100 µm) administration significantly improved the MRSA (USA300) infected wound closure from day 8 to day 14, compared with the nontreated infection group. Moreover, ZLY032 (100 µm) has a better effect on promoting MRSA‐infected wound healing than Penicillin G (100 µm) on day 10. On the third day after ZLY032 administration, the content of MRSA (USA300) in the wound site was detected and the results shown in Figure 5M demonstrated that ZLY032 (100 µm) application significantly decreased the number of colonies compared with the infection group and Penicillin G treated group.

To assess the in vivo efficacy of ZLY032 at its MBC (150 µm), we examined its therapeutic effect on MRSA (USA300)‐infected wounds. As shown in Figure S26A of the Supporting Information, topical application of ZLY032 (150 µm) significantly accelerated wound closure compared to MRSA‐infected controls treated with vehicle (0.2% DMSO), which demonstrated no adverse effects on wound healing in uninfected mice. On day 3 postinfection, ZLY032 (150 µm) treatment yielded a >99.9% inhibition in viable MRSA colonies recovered from wound tissues relative to vehicle‐treated infected controls (Figure S26B, Supporting Information). Concurrently, ZLY032 treatment significantly suppressed levels of inflammatory cytokines IL‐1β and IL‐6 in wound tissues compared with the infected control group (Figure S26C,D, Supporting Information). These results demonstrate that ZLY032 administered at its MBC (150 µm) exerts potent therapeutic effects in MRSA‐infected wounds by concurrently reducing bacterial burden and dampening infection‐driven inflammation.

Furthermore, given that ZLY032 concurrently inhibits bacterial proliferation and promotes wound angiogenesis/anti‐inflammation, its capacity to penetrate bacterial biofilms is functionally critical. Our data confirm that ZLY032 significantly reduces *Staphylococcus aureus* and MRSA biofilm biomass (Figures 4H and 5D). Notably, vancomycin (0–20 µg mL^−1^, 24 h) exhibits no biofilm‐eradicating activity against MRSA biofilms.^[^ ^46^ ^]^ To evaluate biofilm penetration, MRSA (USA300) which has formed biofilms (24 h growth) was treated with ZLY032 (150 µm) or vancomycin (0.5 µm) for 2 h. Live/Dead staining and confocal laser scanning microscopy with 3D reconstruction revealed that in the control group, there was predominantly green fluorescence (viable MRSA) in upper layers, minimal red fluorescence (dead MRSA) in lower strata. In the ZLY032 (150 µm) treated group, there was marked reduction in green fluorescence and significant increase in red fluorescence. This indicates that ZLY032 can penetrate biofilms, kill MRSA, resulting in a significant increase in red fluorescence (dead MRSA) and a significant decrease in green fluorescence (living MRSA). In the vancomycin group, there was persistent green fluorescence in upper layers with limited red signal in deeper regions, demonstrating that vancomycin did not penetrate the biofilm to kill MRSA after acting at a concentration of 0.5 µm for 2 h (Figure S26E, Supporting Information). These findings indicated that ZLY032 effectively decreased the biofilm content of MRSA and penetrates MRSA biofilms within 2 h, which surpasses vancomycin (0.5 µm), so as to promote the healing of MRSA infectious wounds.

### 2.5 Discovering the Target of ZLY032 against *S. aureus* and MRSA

**Figure 6:**
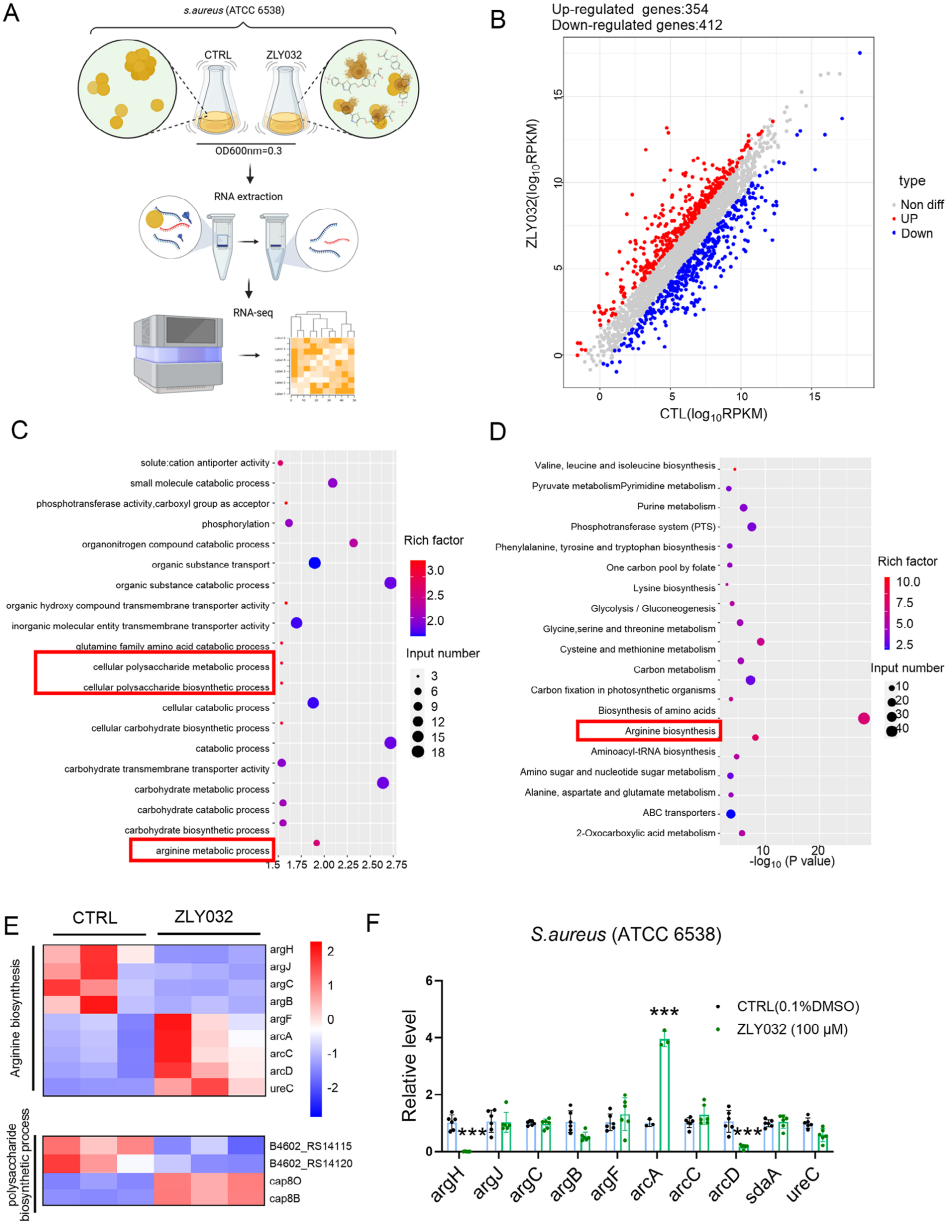
Target identification of ZLY032 in *S. aureus*. A) Flowchart of RNA sequencing in ZLY032‐treated *S. aureus*. B) Gene expression changes in *S. aureus* given ZLY032 treatment. C) GO analysis of differentially expressed genes. D) KEGG analysis of differential genes. E) Differential gene expression analysis screened the genes for significant changes in arginine metabolism and synthesis as well as polysaccharide biosynthesis process after ZLY032 treatment. F) qRT‐PCR detection for the effect of ZLY032 (100 µm) on the expression of the screened genes including argH, argJ, argC, argB, argF, arcA, arcC, arcD, sdaA, and ureC. ***p < 0.001 versus CTRL (0.1% DMSO); *n* = 3–6 for each group. (Student *t*‐test for comparisons between two groups).

**Figure 7:**
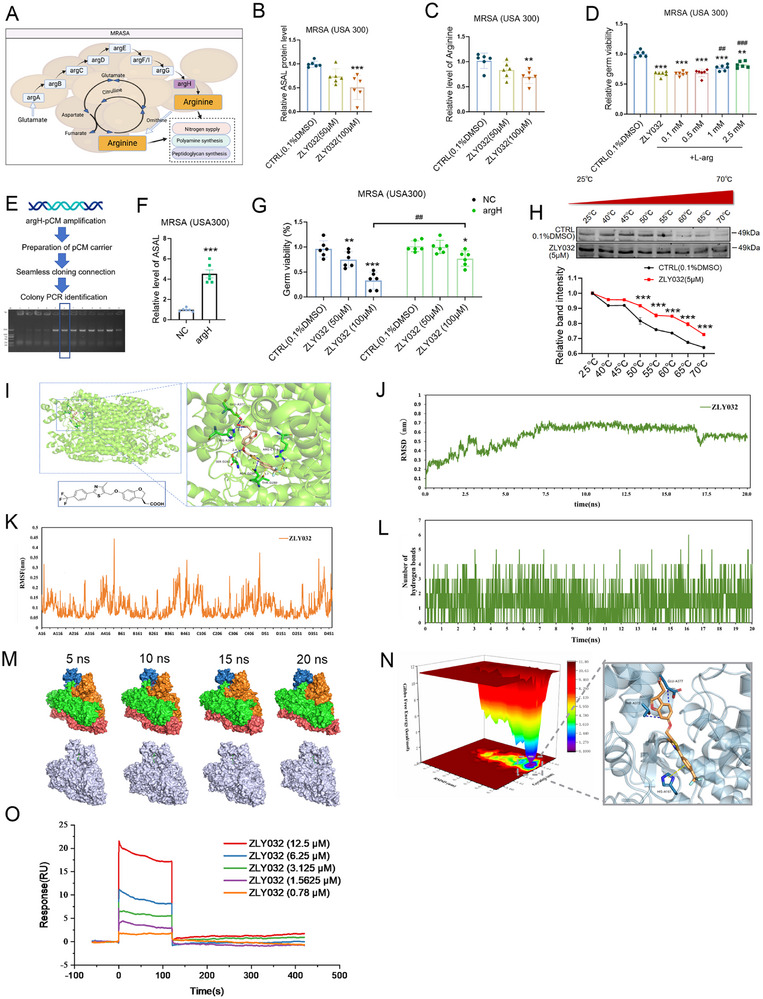
ASAL is the direct target of ZLY032 in *S. aureus*. A) Diagram of arginine synthesis in *S. aureus*. B) ELISA kit to detect the effect of ZLY032 on the expression level of ASAL at 50 and 100 µm. ***p < 0.001 versus CTRL (0.1% DMSO); *n* = 6 for each group. (Mean ± SD; ordinary one‐way ANOVA followed by Tukey's multiple comparisons test among multiple groups.) C) Arginine kit to detect the effect of ZLY032 on the level of arginine at 50 and 100 µm. **p < 0.01 versus CTRL (0.1% DMSO); *n* = 6 for each group. (Mean ± SD; ordinary one‐way ANOVA followed by Tukey's multiple comparisons test among multiple groups.) D) The effect of ZLY032 on MRSA (USA300) after administration of l‐arg (l‐arginine) at concentrations of 0.1, 0.5, 1, and 2.5 mm.**p < 0.001, ***p < 0.01 versus CTRL. ^##^ *p* < 0.01,^###^ *p* < 0.001 versus ZLY032; *n* = 6 for each group. (Mean ± SD; ordinary one‐way ANOVA followed by Tukey's multiple comparisons test among multiple groups.) E) The construction process of stabilized argH overexpression strains. F) ELISA kit to detect the expression level of ASAL in argH overexpressing strains. ***p < 0.001 versus NC. *n* = 6 for each group. (Student *t*‐test for comparisons between two groups.) G) The inhibiting effect of ZLY032 on the strain of MRSA (USA300) with argH overexpression at 50 and 100 µm. *p < 0.05, **p < 0.01, ***p < 0.001 versus CTRL (0.1% DMSO). *n* = 6 for each group. (Mean ± SD; ordinary one‐way ANOVA followed by Tukey's multiple comparisons test among multiple groups.) H) Cellular thermal shift assays (CESTA) to detect the direct binding of ZLY032 on ASAL. ***p < 0.001 versus CTRL (0.1% DMSO); *n* = 3 for each group. (Mean ± SD; ordinary one‐way ANOVA followed by Tukey's multiple comparisons test among multiple groups.) I) Molecular docking of ZLY032 with ASAL. Hydrogen bonds are shown by blue dashed lines, halogen bonds are shown by orange dashed lines, and π–cation interactions are shown by yellow dashed lines. J) Molecular dynamics simulations at the active pocket with 6IG5. RMSD variation curves of the complexes during 20 ns kinetic simulations. K) RMSF values of amino acid residues in the complex system. L) Number of hydrogen bonds between ZLY032 and 6I5G. M) Structures of the complexes of ZLY032 with 6IG5 at 5, 10, 15, and 20 ns. N) Free energy landscape (FEL) plotted between RMSD and gyration coordinates and the energy minimum conformational state of ZLY032. O) SPR assay to determine the direct interaction between ZLY032 and ASAL (from *S. aureus*).

In order to further investigate the target of ZLY032 in inhibiting *S. aureus*, RNA sequencing was conducted (**Figure** **6**A). As illustrated in Figure 6B, compared to the control group, the administration of ZLY032 resulted in upregulation of 354 genes and downregulation of 412 genes. Both Gene Ontology (GO) and KEGG analysis revealed that these differentially expressed genes were primarily related to cellular polysaccharide metabolic and biosynthetic process, arginine biosynthesis, and metabolic process (Figure 6C,D). Additionally, we analyzed the different expressed genes caused by ZLY032 related to arginine biosynthesis and polysaccharide biosynthetic process. As shown in Figure 6E, ZLY032 caused differential expression of several genes that were associated with arginine synthesis including low expression of argH, argJ, argC, argB, and high expression of arcA, arcC, arcD, and ureC in *S. aureus*. Next, qRT‐PCR was employed to examine the impact of ZLY032 on argH, argJ, argC, argB, argF, arcA, arcC, arcD, sdaA, and ureC. Results in Figure 6F displayed that ZLY032 significantly inhibited the expression of argH and arcD, increased the expression of arcA, but showed no effect on argJ, argC, argB, *argF*, arcC, sdaA or ureC. The arginine biosynthesis pathway consists of eight different enzymes encoded by the argC, argJ, argB, argD, argF, argR, argG, and argH genes respectively (**Figure** **7**A), all of which are believed to be necessary for microbial growth.^[^ ^47^ ^]^ Notably, ZLY032 exhibited pronounced inhibitory effect on argH, the gene encoded argininosuccinate lyase (ASAL) (Figure 6F). Moreover, it has been reported that mutation within argH abolished the growth ability of *S. aureus*.^[^ ^48^ ^]^ Therefore, *argH* was speculated to be a target of ZLY032.

To further verify this issue, we first examined the expression difference of argH between *S. aureus* (ATCC6538) and MRSA (USA300). As shown in Figure S27A of the Supporting Information, there was no significant difference between *S. aureus* (ATCC6538) and MRSA (USA300). Second, the effect of ZLY032 on the expression level of argH in MRSA (USA300) was determined. As shown in Figure S27B of the Supporting Information, ZLY032 decreased the mRNA level of argH at the concentration of 100 µm. To verify the reason that ZLY032 decreased the mRNA level of argH, we explored the effects of ZLY032 on the stability of argH mRNA. As shown in Figure S28 of the Supporting Information, ZLY032 significantly inhibited the RNA stability of argH (from *S. aureus*) in actinomycetin D‐treated HEK293T cells from 2, 4, 6, and 8 h, compared with the control group. Moreover, our data demonstrated that ZLY032 could significantly reduce the level of ASAL in MRSA (USA300) at the concentration of 100 µm (Figure 7B). Interestingly, ZLY032 did not affect the expression of argH and ASAL in *E. coli* and *Salmonella* (Figure S29 of the Supporting Information). Then, we investigated whether ZLY032 affected the level of arginine in MRSA (USA300). As demonstrated in Figure 7C, ZLY032 obviously reduced the level of arginine in MRSA (USA300) at the concentration of 100 µm. Furthermore, the inhibitory ability of ZLY032 on MRSA (USA300) was disrupted by administrating l‐arginine (0.1, 0.5, 1, and 2.5 mm) (Figure 7D). To further confirm whether ASAL is the target of ZLY032, we constructed an argH overexpression strain of MRSA (USA300) (Figure 7E). The level of ASAL in the argH overexpression strain was significantly elevated compared to that in the normal control strain, as depicted in Figure 7F. Additionally, we verified the antibacterial activity of ZLY032 on both the normal strain and argH overexpression strains. As shown in Figure 7G, ZLY032 inhibited the proliferation of both the normal MRSA (USA300) and the argH overexpression MRSA (USA300). However, ZLY032 was more effective in inhibiting normal blank strains than argH overexpressed strains. These data suggested that ZLY032 may play an antibacterial role against MRSA by targeting ASAL. To further verify the direct interaction between ZLY032 and ASAL, we first used CETSA to detect the direct binding ability of ZLY032 on ASAL. As shown in Figure 7H, ZLY032 has a potential binding affinity with ASAL.

In addition, computer simulation was adopted to investigate the interaction between ZLY032 and ASAL. Firstly, molecular docking was performed between ZLY032 and ASAL (PDB: 6IG5). As shown in Figure 7I, ZLY032 can match the protein structure of ASAL well. The carboxyl group of ZLY032 forms two hydrogen bonds with the key amino acid residues GLU377A and HIS390A, while the ether bond on the main chain forms a hydrogen bond with SER282D. The thiazole part forms a hydrogen bond with ASN290D. In addition, ZLY032 forms π–cation interactions with THR280D and halogen bonds with ARG114C to stabilize the protein–ligand complex structure. Interestingly, among the above binding sites, except GLU377A of ASAL, the other five sites (HIS390, SER282D, ASN290D, THR280D, and ARG114C) were highly conserved between *Mycobacterium tuberculosis* and *S. aureus* (Figure S30, Supporting Information).

To investigate the binding stability between ZLY032 and ASAL (PDB: 6IG5), we performed 20 ns molecular dynamics simulations using the optimal docking configuration as the initial structure. The root mean square deviation (RMSD) of protein skeleton was recorded to analyze the dynamic stability and structural changes of the complex system. As shown in Figure 7J, we conducted a simulation system with a total of 20 ns. The complex system fluctuated sharply in the range of 0.0–7.5 ns, and the fluctuation tended to be stable obviously after 7 ns, the fluctuation range was ≈0.65 nm, and the value dropped slightly to 0.55 nm when it was ≈17 ns, and then tended to be stable. The covibration of ZLY032 and ASAL (PDB: 6IG5) induces a more stable binding pattern between ZLY032 and the active pocket. This explains why RMSD values level off after a slight drop ≈17 ns. Root mean square fluctuation (RMSF) reflects the motion of each amino acid residue during molecular dynamics simulation. As shown in Figure 7K, the RMSF values of most residues are less than 0.2 nm, which indicates that most residues have relatively stable structures. As shown in Figure 7L,M, the number of hydrogen bonds formed during the simulation is maintained between 1 and 3, and ZLY032 can be stably bound to the active pocket of ASAL (PDB: 6IG5) in the trajectory diagram during the simulation.

Based on the fact that ZLY032 can stably bind to ASAL (PDB: 6IG5), we used the free energy landscape diagram (Figure 7N) to find the lowest energy conformation in the simulation. As the interaction between ZLY032 and the surrounding amino acid residues was greatly changed during the simulation, the carbon atoms on the benzo ring formed two hydrogen bonds with GLU‐A377 and THR‐A373, respectively, and HIS‐A161 formed π–cation interactions with the halogen‐bonded benzene ring. It is worth noting that ZLY032 interacts more tightening with ASAL in the lowest energy conformation, and compared with the initial optimal docking conformation (Figure 7I), GLU‐A377 residues still form hydrogen bonds with ZLY032 in the lowest energy conformation. Considering that ASAL from Mycobacterium tuberculosis as a homology model protein may not be sufficient, we performed the surface plasmon resonance (SPR) to further determine the specific binding between ZLY0332 and ASAL (from *S. aureus*). The results in Figure 7O showed that ZLYO032 had an affinity constant of 10.9 µm with ASAL. These data indicated that ZLY032 had a good combination ability with ASAL and interfered the synthesis of arginine and therefore inhibiting MRSA.

### 2.6 ZLY032‐Loaded Microneedles Promote Wound Healing in Rabbits

**Figure 8:**
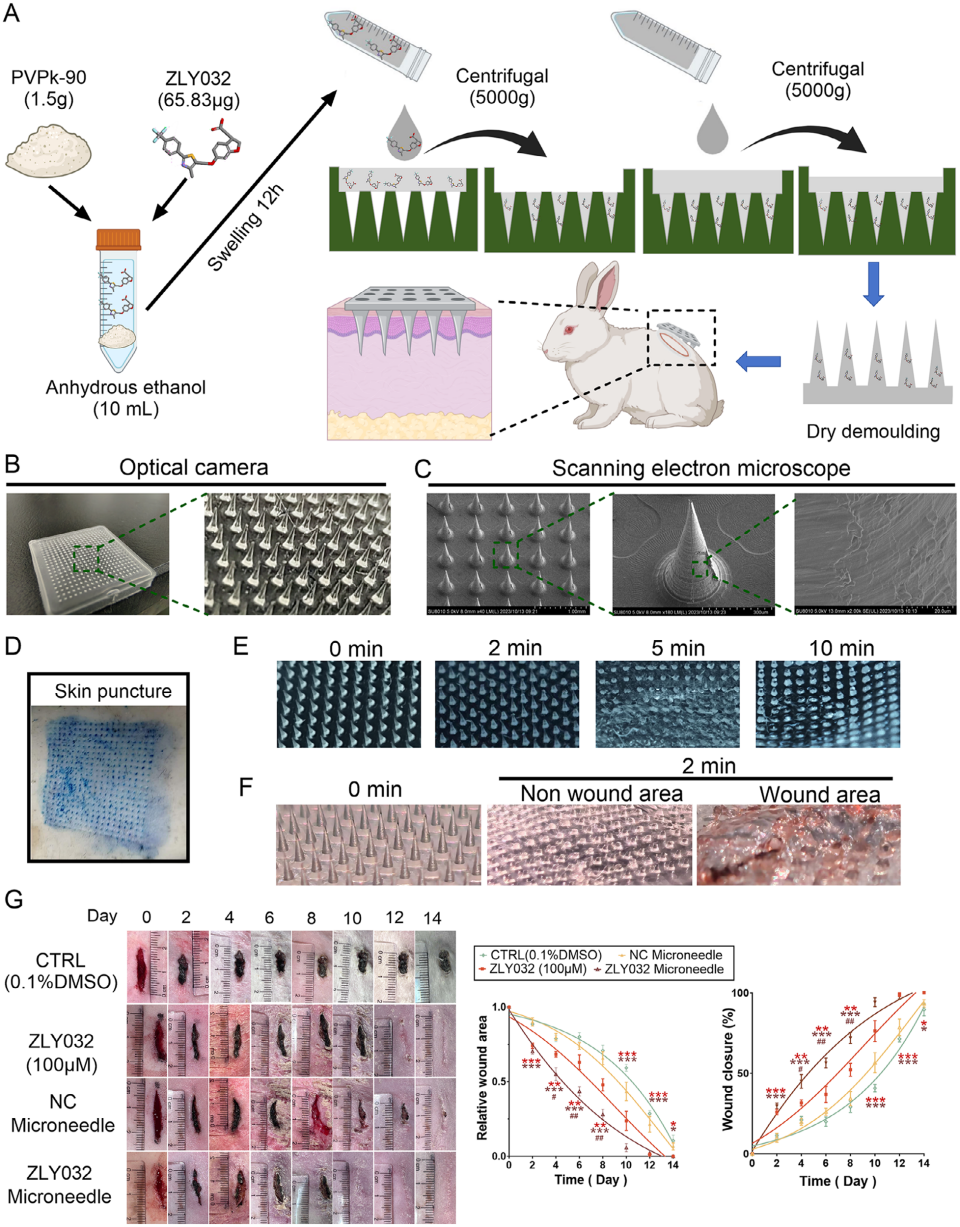
ZLY032‐loaded microneedles promote wound healing in rabbits. A) Microneedle preparation and animal experimental procedures. B) Observation of microneedle morphology. C) Scanning electron microscope observation of microneedle. D) Methylene blue staining to characterize the skin penetration effect of microneedles. E) Microneedle dissolution experiment at general skin. F) Microneedle dissolution experiment at wound area. G) Left panel: representative photographs of time‐dependent wound healing and ZLY032‐loaded microneedles promoting wound healing in rabbits. Empty‐loaded microneedles were used as positive controls. Middle and right panels: wound area and wound closure rate of each rabbit at different time points. **p < 0.01, ***p < 0.001 versus CTRL (0.1% DMSO). ^#^ *p* < 0.05, ^##^ *p* < 0.01 versus NC microneedle; *n* = 4 for each group. (Mean ± SD; two‐way ANOVA followed by Tukey's multiple comparisons test among multiple groups).

In order to deliver ZLY032 better to the dermis of wounds, a ZLY032‐loaded microneedle patch was prepared by using polyvinylpyrrolidone (PVPK‐90) and ethanol (**Figure** **8**A). As shown in Figure 8B,C, the morphology of the prepared microneedles was observed by a high‐resolution microscope and a scanning electron microscope. The microneedle length was 600 µm, the base diameter was 300 µm, the array number was 20 × 20, and the microneedle patch size was 16.7 mm × 16.7 mm. In Figure 8D, neatly arranged microchannels in the mouse skin were observed after applying microneedle patch for 2 min, indicating that the ZLY032‐loaded microneedle had sufficient strength and sharpness to penetrate skin. In addition, ZLY032‐loaded microneedle was determined having a good dissolution efficiency in the skin (Figure 8E). Quantification via LC‐MS revealed an actual ZLY032 loading of 0.410 ± 0.0178 µg per microneedle. In vitro release kinetics demonstrated sustained ZLY032 release over 48 h, with the cumulative release profile shown in (Figure S31A, Supporting Information). CCK‐8 assays confirmed that no detectable cytotoxicity of ZLY032‐loaded microneedles toward HUVECs or human immortalized epidermal keratinocyte cells (Hacats) compared to both untreated controls and blank microneedle groups and even exhibited enhanced proliferation in both cell types (Figure S31B,C, Supporting Information). Moreover, we inserted the ZLY032‐loaded microneedles into the wound area and nonwound area of male mice, and it was observed that most of the tip had dissolved in the wound area (Figure 8F). At 24 h postadministration of ZLY032‐loaded microneedles to infected wounds in mice: the treated wounds exhibited reduction in bacterial load versus controls, significant downregulation of iNos and Tnfα, marked upregulation of IL‐10 and Vegfa (Figure S31D–H, Supporting Information). These data indicated that ZLY032 loaded microneedles did not increase the risk of infection after penetrating the skin. Subsequently, the results of LC‐MS showed that ZLY032 could be detected in local tissues and circulating blood after the ZLY032 loaded microneedle was applied to the wound of mice (Figure S32A,B, Supporting Information). And local application of ZLY032 loaded microneedle does not affect fasting blood glucose (FBG), triglycerides (TG), total cholesterol (TC), low‐density lipoprotein cholesterol (LDL‐C), and high‐density lipoprotein cholesterol (HDL‐C) levels in the circulation system of mice (Figure S32C–G, Supporting Information).

To further investigate the effect of the ZLY032‐loaded microneedle on wound healing, we applied the microneedle patch to the rabbit wound site on the 0th, 3rd, and 7th days, and removed the patch 5 min after the microneedles dissolved completely. The results in Figure 8G demonstrated that the ZLY032‐loaded microneedle significantly promoted wound healing in rabbits compared to the nonloaded microneedle (Figure 8G) The WCT50 of ZLY032‐loaded microneedle was 5 days for rabbit wound, which was obviously shorter than that of ZLY032 nonloaded microneedle group (7 days).

## 3 Discussion

**Figure 9:**
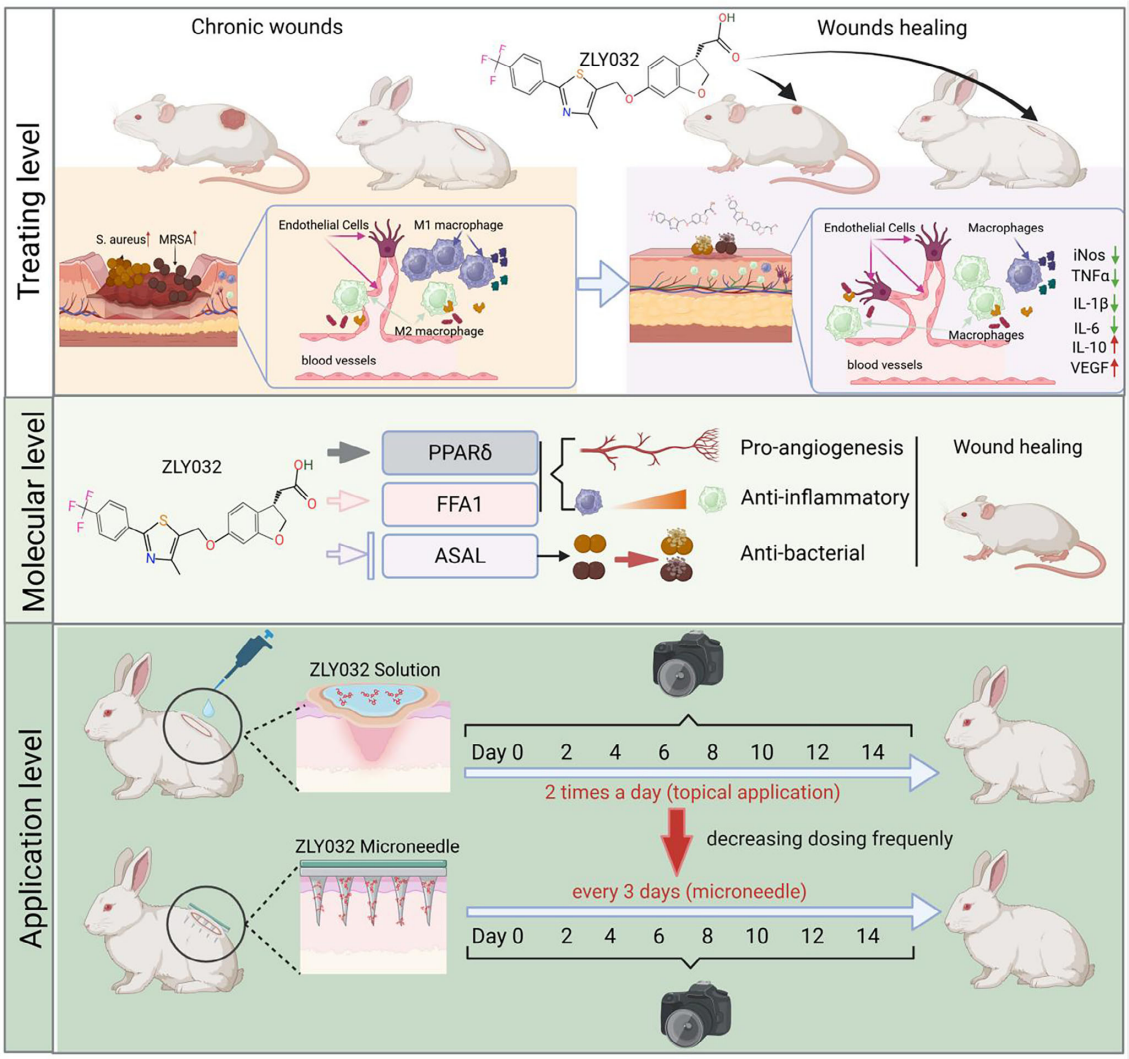
Multifunctional role of ZLY032 in wound healing promotion. At treating level, topical application of ZLY032 effectively accelerated the wound healing processes in both mice and rabbits with low toxicity by promoting angiogenesis, inhibiting inflammation and inhibiting *S. aureus*. At molecular level, ZLY032 acts by targeting PPARδ, FFA1, and ASAL in both eukaryotic and prokaryotic cells. At application level, the microneedles containing ZLY032 can reduce the number of doses and promote wound healing in rabbits.

The current lack of efficient strategy for the treatment of chronic wounds has sparked interest in the development of drugs to promote wound healing. ZLY032 was the first‐in‐class dual FFA1/PPARδ agonist and showed antidiabetic, improved glucolipid metabolism and alleviated hepatic fibrosis effects.^[^ ^17^, ^18^ ^]^ The aims of the present study were to investigate the role of ZLY032 in controlling the healing of wound and to elucidate the underlying mechanisms. Our study first provided strong evidence that topical application of ZLY032 effectively accelerated the wound healing processes in normal mice, diabetic mice, rabbits, and MRSA infected mice with low toxicity. Moreover, our results demonstrated that the wound healing‐promoting properties of ZLY032 could be ascribed to its ability to promote angiogenesis, inhibit inflammation, and inhibit *Staphylococcus aureus*, especially MRSA. Our data also demonstrated that ZLY032 acts by targeting PPARδ, FFA1, and ASAL (ArgH) in both eukaryotic and prokaryotic cells. In addition, the microneedles containing ZLY032 can reduce the dosing frequency and promote wound healing in rabbits, reflecting the application value of ZLY032 (**Figure** **9**).

Angiogenesis is a rate‐limiting step in wound healing and vascular endothelial dysfunction delayed wound healing.^[^ ^49^, ^50^ ^]^ Our data unraveled that ZLY032 not only promoted wound healing in normal mice and rabbits, but also in diabetic mice. ZLY032 showed reactivate ability in the processes of neovascularization. When ZLY032 was administered to normal wounds with increased blood flow as well as to diabetic mice. Masson staining showed increased vascular density at the wound site after ZLY032 administration. At the cellular level, ZLY032 treated HUVEC cells with enhanced cell proliferation, migration, and tube formation. In addition, we found that ZY032 can also indirectly promote endothelial proliferation, migration, and tube formation by affecting macrophages via coculture experiments. It has been reported that PPARδ activation increased VEGF expression in cancer cells and HUVEC cells.^[^ ^36^ ^]^ Our results showed that the promotion effect of ZLY032 on the proliferation, migration, and tubularization of endothelial cells was significantly weakened after the administration of PPARδ and FFA1 inhibitors or knockdown PPARδ and/or FFA1 in HUVEC cells. Moreover, ZLY032 exerted promoting angiogenesis effects by increasing the level of VEGF, the downstream of PPARδ and FFA1.

The transition from inflammation to proliferation is a critical step for wound healing.^[^ ^51^, ^52^ ^]^ At the wound site, excessive proinflammatory cytokines accumulation and anti‐inflammatory factors deficiency can lead to a vicious circle of chronic inflammation, resulting in chronic or even nonhealing wounds.^[^ ^31^, ^53^, ^54^ ^]^ In vivo, ZLY032 treatment significantly reduced IL‐1β and IL‐6 levels while increasing the population of reparative macrophages (CD11b^+^ CD206^+^) in wound tissues. In LPS‐stimulated macrophages, ZLY032 significantly inhibited the expression levels of proinflammatory factors iNos and Tnfα, and increased the expression levels of anti‐inflammatory factors including IL10 and Vegfa. Moreover, knockdown of PPARδ and/or FFA1 or administration of PPARδ and FFA1 inhibitors significantly interferes with the effect of ZLY032 on inflammatory factors in RAW264.7 cells. Furthermore, we verified that ZLY032 exerted anti‐inflammatory effects by inhibiting the expression of pNF‐κB p65, the downstream of PPARδ and FFA1. The pain response of chronic wounds seriously reduces the life quality of patients. COX‐2 is a key target for inflammation and pain, and inhibition of COX‐2 has anti‐inflammatory and pain‐inhibiting effects. We unexpectedly found that ZLY032 had a significant inhibitory effect on COX‐2. This may be an important reason for its anti‐inflammatory effect, and ZLY032 may have analgesic effect to release the pain of the patient with wounds. Additionally, we found that ZLY032 exerted effects not only on macrophages and endothelial cells, but also on adipocytes (inhibiting expression of inflammatory cytokines including IL‐1β and IL‐6; promoting expression of growth factor Fgf) and epithelial cells (promoting proliferation and migration) via targeting PPARδ and FFA1. Therefore, ZLY032 can achieve wound healing by regulating a variety of wound healing relative cells.

During the wound healing process, bacterial infection is another important factor causing persistent inflammatory response and delaying healing process.^[^ ^55^, ^56^ ^]^ *S. aureus* is a common opportunistic pathogen, and its infection can cause a series of diseases including chronic wounds. The severe drug resistance caused by *S. aureus* has brought great difficulties to clinical treatment.^[^ ^57^, ^58^ ^]^ Through a series of experimental studies, we found that ZLY032 not only has an inhibiting effect on *S. aureus*, but also can inhibit the proliferation of MRSA. In vivo, ZLY032 significantly promoted the healing of MRSA‐infected wounds by inhibiting the proliferation of MRSA at the wound site. At molecular level, the ASAL protein encoded by argH was identified as a potential target of ZLY032 by RNA sequencing and qRT‐PCR. Moreover, we demonstrated that ZLY032 can inhibit the mRNA stability of ArgH. This is why ZLY032 inhibits the mRNA levels of ArgH. ASAL is a key enzyme for bacteria to synthesize arginine, which is an important amino acid for maintaining bacterial growth and metabolism. Inhibition of arginine biosynthesis can combat methicillin‐resistant *Staphylococcus aureus*.^[^ ^47^, ^59^ ^]^ Our results demonstrated that ZLY032 inhibited ASAL and arginine levels in *S. aureus*, but not in *E. coli* and *Salmonella*. SPR, CETSA combining with the computer simulation of molecular docking and molecular dynamics further verified that ZLY032 directly binds to ASAL with high stability. And the potential binding sites are GLU377, HIS390, ASN290D, SER282D, THR280D, and ARG114C. These sites may contribute to the development of new antibiotics against drug resistant strains. In addition, these sites can also be used as a basis for screening other PPARδ and FFA1 agonists for antimicrobial effects or for developing multitarget wound‐healing agents.

At the level of medical application, our study demonstrated that the microneedle patch containing ZLY032 had a promoting effect on wound healing in rabbits, and greatly reduced the dosing frequency of ZLY032. Following topical administration via ZLY032‐loaded microneedles in murine wound models, ZLY032 exhibited both local tissue retention and limited systemic absorption into circulation. However, no significant alterations were observed in circulating metabolic markers including FBG, TG, TC, LDL‐C, or HDL‐C levels. This contrasts with our previous findings demonstrating that oral ZLY032 administration (40 mg kg^−1^) significantly reduced atherogenic lipids (TC, TG, and LDL‐C) in ob/ob mice.^[^ ^17^ ^]^ The observed differential effects may be attributed to the substantially lower systemic exposure achieved by topical delivery (plasma concentration: 0.1–0.15 µg mL^−1^), which appears insufficient to elicit detectable metabolic modulation compared to therapeutic oral dosing. As shown by Saghazadeh et al., the systemic circulation exposure of drugs after local delivery for wound healing is extremely low.^[^ ^60^ ^]^ Similarly, our findings revealed that ZLY032 was undetectable in the bloodstream following topical application of ZLY032 solution. In contrast, Anjani et al. reported detection of carvedilol in the systemic circulation after microneedle‐mediated transdermal administration,^[^ ^61^ ^]^ indicating that microneedle‐based drug delivery enhances the capacity of drugs to permeate through the skin into the bloodstream. This mechanism elucidates why ZLY032 could be detected in the blood circulation of mice after applying ZLY032‐loaded microneedles to wound sites.

It has been reported that the activation of PPARβ/δ accelerated the diabetic wound healing.^[^ ^22^, ^62^ ^]^ Lu et al. had found that FFA1 is associated with inflammatory cytokine expression and cell migration and is a potential target for treatment of wound healing.^[^ ^63^ ^]^ Guan et al. demonstrated that PPARβ and FFA1 dual agonist Y8 promoted diabetic wound healing.^[^ ^22^ ^]^ We demonstrated that ZLY032 ameliorates the metabolic microenvironment in diabetic wounds via decreasing MDA level, increasing SOD level, inhibiting ROS and AGEs. Substantial evidence implicates PPAR signaling in the modulation of oxidative stress, with PPAR agonism demonstrated to suppress oxidative damage by reducing ROS levels, and also, activation of PPAR decreased MDA and restored superoxide dismutase SOD level.^[^ ^64^, ^65^, ^66^ ^]^ Similarly, FFA1 agonism has been shown to attenuate oxidative stress.^[^ ^67^ ^]^ Our findings establish that dual agonism of FFA1 and PPARδ by ZLY032 inhibits activation of the NF‐κB pathway. This observation is mechanistically significant, as NF‐κB suppression is known to inhibit AGEs formation and restore redox homeostasis by normalizing MDA and SOD levels.^[^ ^68^, ^69^ ^]^ Collectively, these established mechanisms and our experimental data support the conclusion that compound ZLY032 ameliorates the diabetic wound metabolic microenvironment through its dual‐targeted agonism of FFA1 and PPARδ.

The advantages of ZLY032 in promoting wound healing were that it has multitarget and multifunctional effects including promoting angiogenesis and anti‐inflammatory on eukaryotic cells and antibacterial effect on prokaryotic cells. Although, ZLY032 has less inhibitory effect on MRSA than vancomycin and linezolid at concentrations below 100 µm. However, ZLY032 has the advantage of multiple functions, that is, proangiogenesis, anti‐inflammatory, and antibacterial effects. Specially, ZLY032 effectively decreased the biofilm content of MRSA and penetrates MRSA biofilms within 2 h, which surpasses vancomycin (0.5 µm). Thus, while ZLY032 requires a higher MBC (150 µm) than conventional antibiotics, its dual mechanisms—biofilm suppression and deep penetration—provide a distinct therapeutic advantage against biofilm‐embedded MRSA. Moreover, the ability to penetrate biological membranes also enables ZLY032 to target both prokaryotic cells (MRSA) and eukaryotic cells (vascular endothelial cells, macrophages, epithelial cells, and adipocytes) simultaneously at the wound site to exert its effects. The low toxicity and versatility of ZLY032 in eukaryotic cells, as well as its stable killing properties against *S. aureus* and MRSA, make it more likely to be developed as a therapeutic agent for the treatment of refractory wounds such as diabetic wounds and drug‐resistant bacteria infected wounds. In addition, the research on the antimicrobial mechanism of ZLY032 also laid the foundation for the development of new antibiotics against resistant *S. aureus*. Previous studies have confirmed that oral administration of ZLY032 can regulate glucolipid disorders and improve liver fibrosis in mice, and our study confirmed that topical ZLY032 can promote wound healing, suggesting that ZLY032 has the potential to be developed into a multitherapeutic drug. Our research also provides theoretical support for the development of multitarget therapeutic drugs and even antibiotics for drug resistant strains.

There are some limitations of our study. First, the inhibitory sites of ZLY032 on argH mRNA and ASAL may be different among species. Therefore, ZLY032 only inhibited S *aureus*, but not *E. coli* or *Salmonella*. It still needs further experimental verification. Second, in this study, we verified ZLY032 may bind to GLU377, HIS390, ASN290D, SER282D, THR280D, and ARG114C in ASAL and inhibit the synthesis of arginine and therefore has an anti‐MRSA effect. However, we did not mutate these binding sites and verify the function of these sites in the living activity of *S. aureus*. Third, we did not demonstrate the molecular mechanism of ZLY032 in inhibiting and penetrating MRSA biofilms, which will be completed in the following study. At last, as for the synthesized microneedles, their performance needs to be further optimized, and interdisciplinary research is needed in the future.

Overall, the highlight of our study is that ZLY032 plays a multifunctional role in promoting skin wound healing via targeting both eukaryotic and prokaryotic cells, which makes ZLY032 have potential translational value in the field of wound healing.

## 4 Experimental Section

### Study Approval

The experimental protocols involving the use of animals in this study were approved by the Animal Care and Use Committee of Harbin Medical University (HMUIRB3041724), and are conformed to the Guidelines for the Care and Use of Laboratory Animals set forth by the US National Institutes of Health (NIH Publication No. 85–23, revised 1996).

### The Synthesis Procedure and Characterizations of Compound ZLY032

ZLY032 was synthesized according to the previous study^[^ ^18^ ^]^ and the reaction process depicted in Figure S33 the Supporting Information. Ethyl 4‐methyl‐2‐(4‐(trifluoromethyl)phenyl)‐thiazole‐5‐carboxylate (1) was synthesized by treating 4‐(trifluoromethyl)benzothioamide with ethyl 2‐chloroacetoacetate in refuxing ethanol for 6 h in the presence of Na_2_CO_3_. 5‐(chloromethyl)‐4‐methyl‐2‐(4‐(trifluoromethyl)phenyl) thiazole (2) was accordingly prepared by the reaction of compound 1 and NaBH4 in refluxing THF for 0.5 h in the presence of methanol. The reaction of 2‐(6‐hydroxy‐2,3‐dihydrobenzofuran‐3‐yl) acetic acid and (R)‐1‐phenylethan‐1‐amine in refluxing acetone for 1 h resulted in the formation of intermediate product (3). This intermediate 3 subsequently underwent esterification by the addition of 1 m hydrochloric acid and the dropwise addition of sulfuric acid to methanol, followed by reflux for 4 h to obtain methyl (S)‐2‐(6‐hydroxy‐2,3‐dihydrobenzofuran‐3‐yl)acetate (4). Products ZLY032 was synthesized through the reaction of compound 2 and compound 4 in acetonitrile with K_2_CO_3_ as a base at 45 °C for 12 h. Finally, ZLY032 was obtained by adding lithium hydroxide to a mixed solution of THF/methanol/water. After stirring at room temperature for 4 h, the volatiles were removed under reduced pressure. The residue was acidified with 1 N hydrochloric acid solution, and then filtered and the filter cake was dried in vacuum, recrystallization from 75% ethanol affords ZLY032 as a white powder, m.p. 208–210 °C; [α]D +4.8° (c 0.3020, CH3CN), 99.1% ee. ^1^H NMR (300 MHz, DMSO‐d6) δ: 12.39 (s, 1H), 8.09 (d, J = 8.0 Hz, 2H), 7.81 (d, J = 8.0 Hz, 2H), 7.16 – 7.13 (m, 1H), 6.52 (m, 1H), 5.27 (s, 2H), 4.71 (t, J = 9.0 Hz), 4.28 – 4.10 (m, 1H), 3.70 – 3.68 (m, 1H), 2.74, 2.69 (dd, J = 16.5, 5.5 Hz, 1H), 2.55, 2.50 (dd, J = 16.5, 9.0 Hz, 1H), 2.45 (s, 3H, CH3). ^13^C NMR (75 MHz, DMSO‐d6) δ: 173.59, 163.75, 161.21, 158.99, 152.23, 136.87, 129.43, 126.99, 126.55, 125.14, 123.05, 107.46, 97.48, 77.67, 62.29, 40.79, 37.57, 15.51. TOF‐MS m/z: calcd for C22H17F3NO4S‐ [M‐H]‐: 448.0836, found: 448.0834. Anal. calcd. For C22H18F3NO4S: C, 58.79; H, 4.04; N, 3.12; Found: C, 58.65; H, 4.06; N, 3.11.

### Mouse Wound Healing Model

The mouse model of wound and wound‐healing analysis were carried out on the basis of previous studies^[^ ^70^ ^]^ with some modifications. Briefly, Kunming male or female mice (25–30 g; Liaoning Changsheng Biotechnology Co, China) were used for the experiments and housed in single cages. The mice were fully anesthetized with tribromoethanol (2%, 10 µL g^−1^; Sigma, St. Louis, MO, USA) and the hair on the back of the mice was cleaned off. A single round‐shape full‐thickness wound with a diameter of 1 cm was created using a sterile punch biopsy. Apply the appropriate medication to the incision and allow full contact of the medication with the wound. Digital images of wound were obtained every two days from day 0 to day 14 postwounding for monitoring the time course of wound healing. Each time wound images were taken, the whole mouse was placed on a graduated background plate to ensure that the initial wound area of the mouse was consistent (Figure S34, Supporting Information). The area surrounded by visible edge of dermis was defined as the wound area, which was measured by using the image analysis software Image Pro Plus and pixel values were recorded to calculate the percentage of wound closure.

Skin‐specific PPARδ and/or FFA1 knockdown wound model: two days before injury (pre‐day 2), a 1‐cm‐diameter circle was marked on the mouse's back. siNC‐m, PPARδ‐m, siFFA1‐m, and PPARδ‐m + siFFA1‐m (2.5 nmol, MingSheng Bio, siRNA sequences are provided in Table S3, Supporting Information) were subcutaneously injected along the circle's edge. On day 0, a 1‐cm‐diameter full‐thickness skin punch wound was made. From day 0, 2.5 nmol of siRNAs or siNC was administered to the wound every two days.

### Dissolution and Topical Application of ZLY032

For the ZLY032 administration group: different amounts of ZLY032 were dissolved in DMSO and added into phosphate buffer saline (PBS) solution, so that the proportion of DMSO in the solution was 0.1%, and the final concentration of ZLY032 was 5, 50, and 100 µm. A solution containing the above concentration of ZLY032 was topically applied onto the wound area of mice twice a day at 10 µL each time. For the CTRL group: DMSO was added into PBS solution and the proportion of DMSO in the solution was 0.1%. PBS solution containing DMSO (0.1%) was topically applied onto the wound area of mice twice a day at 10 µL each time.

### The Dissolution and Topical Application of PDGF and Penicillin G on Wound

PDGF purchased from MedChemExpress (Cat#HY‐P7055, MCE, USA) was added into the PBS solution, which containing 0.1% DMSO, and the final concentration of PDGF was 50 ng mL^−1^. The PDGF containing solution was topically applied onto the wound area of mice twice a day at 10 µL each time. Penicillin G purchased from MedChemExpress (Cat#HY‐17591, MCE, USA) was added into the PBS solution, which containing 0.1% DMSO, and the final concentration of Penicillin G was 5, 50, and 100 µm. The Penicillin G containing solution was topically applied onto the wound area of mice twice a day at 10 µL each time.

### Rabbit Wound Healing Model

The same procedures were followed for developing a rabbit model of wound as described previously^[^ ^71^ ^]^ with some modifications. Briefly, male white rabbits (15–17 weeks old, 2–2.5 kg; Liaoning Changsheng Biotechnology Co, China) were anesthetized with urethane (20%; Shanghai Shanpu Chemical Co, China), the hair on the back of the rabbits was shaved off and sterilized with povidone‐iodine. Four 2 cm wounds were made on the back of each rabbit with a scalpel. The corresponding medication was topically applied to the incisions twice a day with the volume of 20 µL each time. A camera (Nikon D300; Nikon, Melville, NY, USA) with standardized exposure and focal length was used to record the wound area at day 0 (the day that the wound opened was assigned as Day 0), 3, 5, 7, 9, 11, 13, and 15. Every time taking a picture of the wound, a ruler was placed near the wound. The vertical distance from the lens to the wound was kept as 25 cm. Image Pro Plus software was used to count the unhealed area of the wounds. Percentage of wound closure at each time point was calculated as: wound closure (%) = ((wound area day 0 – wound area day *n*)/wound area day 0) × 100, with *n* = from Day 3 to the Day 15.

### Type 1 Diabetes Mellitus (DM) Mouse Model of Wound

The DM mouse model of wound and wound‐healing analysis were conducted as described previously.^[^ ^70^ ^]^ Kunming male mice (25–30 g; Liaoning Changsheng Biotechnology Co, China) were used for the experiments. Food and water were provided 1 week before the experimental procedures, and all mice were treated with a 12 h fast at the night before modeling. A single high‐dose intraperitoneal injection of streptozotocin (150 mg kg^−1^; STZ; Sigma, St Louis, MO, USA) dissolved in citrate buffer (pH 4.5) was administered intraperitoneally to mice provided with glucose water for 12 h after the injection. Seven days after streptozotocin injection, blood was obtained from the tail vein of mice and FBG levels were measured using a Roche blood glucose meter (Roche, Germany), and the DM model was considered successfully established in mice with two consecutive random blood glucose values >11.5 mmol L^−1^. Based on this, a mouse wound model was established, with a circular wound diameter of 0.5 cm and the medication (the volume was 10 µL) was administered twice a day. Photographs were taken and recorded at day 4, 8, 12, and 14 of the wounds on the back of the mice. Image Pro Plus software was used to count the unhealed area of the wounds and the wound closure rate.

### Assessment of Blood Flow and Vascular Recovery in Mouse Wound Healing

On the eighth day of the mouse wound model (DM model: day 14), male mice were fully anesthetized with tribromoethanol (2%, 10 µL g^−1^; Sigma, St. Louis, MO, USA), the recovery of blood flow at the wound was detected and recorded using a Doppler flow detector (Gene&I, China). The male mice were executed, and the skin on their backs was removed and recorded of blood vessels in the wound with a microscope. Images as well as data were processed using moorFLPI and Image Pro Plus.

### MRSA‐Infected Mouse Model of Wound

The MRSA‐infected wound model in mice was constructed according to the previous studies with some modifications.^[^ ^72^, ^73^ ^]^ The wounds, according to the Mouse Wound Healing Model described above, were infected with MRSA (1.25 × 10^9^ CFU mL^−1^, 20 µL) at pre‐day 2 and pre‐day 1 and MRSA (1.25 × 10^9^ CFU mL^−1^, 10 µL) day 0, day 2, day 4, day 6, day 8, day 10, day 12, and day 14, totally 1.5 × 10^8^ CFUs per mouse. Male mice infected with MRSA were randomized into groups including Infection group, Infection+ZLY032 (50 µm) group, Infection+ZLY032 (100 µm) group, Penicillin G (100 µm) group. Infection group: a PBS solution containing 0.1% DMSO was administered topically twice a day at 10 µL each time. Infection+ZLY032 (50 or 100 µm) group: ZLY032 was dissolved in DMSO and added to PBS solution, so that the final concentration of DMSO was 0.1% and the final concentration of ZLY032 was 50 or 100 µm, and the final solution was administered topically to the wound twice a day, at 10 µL each time. Penicillin G (100 µm) group: Penicillin G was dissolved in PBS solution and DMSO was added to this solution, so that the final concentration of DMSO was 0.1% and the final concentration of Penicillin G was 100 µm, the final solution was administered topically to the wound twice a day, at 10 µL each time. The unhealed wound size was measured using the Image Pro Plus and pixel values were recorded to calculate the percentage of wound closure.

### The Bacterial Content in the Wound Tissue from MRSA‐Infected Mouse Model

The traumatic tissue from the above mentioned MRSA‐infected mouse model of wounds was taken on the third day and ground using a sterile mortar and pestle until it was free of lumps to the naked eye, weighed and diluted with PBS to a uniform concentration of 10 mg mL^−1^.

The tissue grinding solution was taken for gradient dilution 1000 times, coated on LB solid culture plates, repeated three times to ensure uniform coating, and continued to be incubated in a bacterial incubator at a constant temperature of 37 °C for 24 h, and then photographed to record the density of the distribution of the plate colonies of the different groups, and then analyzed the tissue bacterial content semiquantitatively to evaluate the effect of bacterial inhibition in vivo.

### LC‐MS to Detect the Concentration of ZLY032 in the Blood and Wound Tissue of Wound Model Mice

A liquid chromatograph mass spectrometer (LC‐MS) (MS: SCIEX4500, USA; HPLC: Shimadzu LC‐20A, Japan) was used to measure the concentration of ZLY032 in the blood of wound model mice. The HPLC conditions were set as follows: Shim‐pack GIST CI8‐AQ HP, 1.9 µm, 2.1 × 50; Flow rate: 0.3 mL min^−1^; Column temperature: 40 °C; Injection volume: 1 µL, mobile phase: phase A: water (containing 0.05% formic acid), phase B: methanol. Gradient elution: 0.01 min (B: 80%), 2 min (B: 100%), 4 min (B: 100%), 5 min (B: 80%). The mass spectrometry conditions were set as follows: ion source temperature: 200 °C, ion source voltage: −4500 V; Curtaingas: 35 psi; Collision gas: 9 psi; Atomization gas (lonsource gas1): 50 psi: dry gas (Lonsource GAS2): 50 psi: ESI ion source, MRM detection mode for negative ion detection: ZLY032 (Q1Mass: 448.1 Da; Q3Mass: 404.2 Da; DP‐93.126 V: CE: −23.175 V); Internal standard (QlMass: 398.1 Da; Q3Mass: 354.1 Da: DP: −90.67 V; CE: −20.54 V). Here, Q1Mass is the molecular weight of the parent ion, Q3Mass is the molecular weight of the daughter ion, DP is the decluster voltage, and CE is the collision voltage. To establish a standard curve, ZLY032 and the internal standard compound were each prepared as stock solutions of 10 mg mL^−1^ using methanol. The stock solution was diluted in gradient to 1000, 500, 250, 100, 50, 25, 10, and 5 ng mL^−1^ standard solutions. An internal standard compound of 500 ng mL^−1^ was used as an internal standard for the standard curve. Standard solution (50 µL), internal standard solution (50 µL) and methanol (200 µL, containing 0.2% formic acid) were added successively to mouse plasma (50 µL). The mixture was mixed in a vortex oscillator for 3 min, centrifuged at 12 000 r min^−1^ for 10 min, and the supernatant was taken for testing. The concentration of the substance to be measured was plotted on the abscissa, and the ratio of the peak area of the substance to the internal standard was plotted on the ordinate to make a standard curve. Blood samples were collected at 0.5, 1, 2, 4, and 8 h after ZLY032 administration in the wound model mice on day 0, day 3, and day 14 after wound creating. The collected blood was centrifuged at 12 000 r min^−1^ for 10 min and the obtained plasma (50 µL), blank methanol (50 µL), internal standard solution (50 µL), and methanol (200 µL, containing 0.2% formic acid) were mixed and centrifuged at 12 000 r min^−1^ for 10 min, and the supernatant was loaded to LC‐MS. The peak area ratio of the tested substance to the internal standard was calculated and substituted into the standard curve to obtain the plasma concentration of ZLY032.

For ZLY032 tissue retention: construct a wound mouse model with a diameter of 1 cm according to the previous method. Skin tissues were collected and weighed at 0, 0.5, 1, 4, 8, and 12 h after treatment with ZLY032 (100 µm, 10 µL), 0.9% NaCl was added at a skin tissue mass/volume ratio of 1:4, and a homogenate was prepared. 100 µL of the tissue homogenate was taken into a tube, 100 µL of internal standard solution and 600 µL of methanol (containing 0.2% formic acid, analytical pure) were added, mixed in a vortex oscillator for 3 min, centrifuged at 12 000 rpm for 10 min, and loaded the supernatant. The aforementioned LC‐MS methodology was employed to determine the concentration of ZLY032 within the sample, and ultimately the tissue content of ZLY032 was computed.

### Microneedle Preparation

The ZLY032 (65.83 µg) was dissolved in 10 mL of anhydrous ethanol, and 1.5 g of PVP K90 (MB1925, Meilun, China) was mixed for 12 h. The drug‐containing microneedle tip matrix solution was prepared. Approximately 200 µL of tip matrix solution was added to the microneedle mold and centrifuged at 5000 × *g* for 5 min at room temperature. The mold was removed to scrape off the excess tip solution and left to dry at room temperature. After drying, ≈300 µL of airborne material was injected to make the backing layer and centrifuged at 5000 × *g* for 5 min at room temperature. Finally, the molds were placed in a desiccator for overnight drying and demolding.

### Microneedle Smorphology Evaluation

The shape and height of the fabricated microneedles were examined using an optical microscope and a scanning electron microscope, respectively. The resulting microneedles comprised a conical needle body and a backing layer, arranged in a 20 × 20 array configuration. Inspection revealed that the array was intact, the backing layer was devoid of bubbles, and the needle shape was well‐formed.

### In Vivo Dissolution Test of Microneedles

One day before the experiment, the rabbits were anesthetized with urethane (20%; Shanghai Shanpu Chemical Co, China). Subsequently, the hair on the back of the animals was shaved using a shaver, and the prepared microneedles were lightly pressed against the skin on the back of the animals with a force of ≈5 N and maintained for 0, 2, 5, and 10 min, respectively, to observe the dissolution of the microneedles in vivo.

### In Vitro Evaluation of Microneedle Skin Penetration

Prior to experimentation, male mice were anesthetized as previously described and euthanized by cervical dislocation following approved animal welfare protocols. The dorsal skin was carefully excised, thoroughly rinsed with 0.9% saline, and excess moisture was removed using filter paper. The prepared skin samples were then mounted on a rigid platform for microneedle application. Microneedles were applied to the skin surface with a controlled force of approximately 5 N using a mechanical press. To visualize penetration, the treated skin areas were stained with 0.1% methylene blue solution for 5 min, followed by gentle rinsing with saline to remove excess dye. Successful penetration was characterized by the presence of distinct blue microchannels corresponding to microneedle insertion sites.

### Application of Dissolving Microneedles in Rabbit Wound Healing Model

The hair on the back of the rabbits was removed, and the microneedle was placed at the wound (edge of the scabbed wound) and pressed with a force of about 5 N until the microneedle was completely adhered to the skin for 10 min, and then removed, and the dissolution of the microneedle at the tip was observed. The treatment was given every three days, and the photos were recorded during the wound healing status every two days. Image Pro Plus software was used to count the area of unhealed wounds and the rate of wound closure.

### Masson Staining

Using Masson Trichrome Staining Kit (G1340, Solarbio, China), the sections were deparaffinized; stained with weigert iron hematoxylin for 5–10 min and rinsed with running water; then differentiated with 1% hydrochloric acid in alcohol and rinsed with running water; stained with Rexigen red acidic magenta stain for 5–10 min and rinsed with running water; treated with phosphomolybdic acid solution for ≈5 min, and directly stained with aniline blue dye solution for 5 min; treated with 1% glacial acetic acid for 1 min, dehydrated with 95% alcohol for several times and then sealed with a sealing agent (S2150, Solarbio, China).

### HE Staining

The paraffin sections were stained with HE staining kit (G1120, Solarbio, China), and the processed paraffin sections were differentiated by 1% hydrochloric acid in ethanol, rinsed under running water for 2 min. The specimen was immersed in a NaHCO_3_ solution, stained with eosin for 1 min, air‐dried for 30 min, and then sealed with a sealing agent (S2150, Solarbio, China).

### Immumohistochemical Staining

The skin tissue samples were fixed, embedded in paraffin, dewaxed, and rehydrated with graded ethanol. Then, endogenous peroxidases were quenched and antigens were retrieved. Nonspecific protein binding was blocked with serum. The samples were incubated with a primary antibody against AGE (HY‐P818087, MCE, China), followed by color development using a universal SP kit (SP0041, Solarbio, China). Positive signals were indicated by brownish‐yellow particles.

### Measurement of Metabolic Parameters in Plasma after ZLY032 Treatment

After administering ZLY032 (100 µmol, 10 µL) or PBS (0.1% DMSO) to the wound site for 0.5 h, peripheral blood was collected from the mice and the plasma was isolated. The FBG levels in mice were assayed using a Roche glucometer. Triglyceride test kit (A110‐1‐1, Nanjing Jiancheng Bioengineering Institute, China), total cholesterol test kit (A111‐1‐1, Nanjing Jiancheng Bioengineering Institute, China), low‐density lipoprotein (LDL) test kit (A113‐1‐1, Nanjing Jiancheng Bioengineering Institute, China), and high‐density lipoprotein (HDL) test kit (A112‐1‐1, Nanjing Jiancheng Bioengineering Institute, China) were used to determine plasma TG, TC, LDL, and HDL levels according to the manufacturer's protocol.

### Immunofluorescence (IF)

Skin tissue sections were immobilized with a hydrophobic barrier pen. They were fixed with 4% paraformaldehyde for 15 min, followed by three rinses with PBS (each for 5 min). Permeabilization was conducted with 0.2% Triton X‐100 (P0096, Beyotime Biotechnology, China) for 12 min. Subsequently, the sections were blocked with 5% goat serum (C0265, Beyotime Biotechnology, China) for 30 min. Staining was performed with an ROS assay kit (MA1301‐1, Meilunbio, China). After that, DAPI (1 µg mL^−1^, 10 min, #D9542, Sigma‐Aldrich, USA) was utilized for nuclear counterstaining. The sections were mounted with ProLong Gold Antifade reagent (P0126, Beyotime Biotechnology, China) and imaged using a Zeiss LSM 800 confocal microscope. The fluorescence intensity was quantified using ImageJ.

### Culture and Treatment of HUVECs, RAW264.7, 3T3‐L1, and NCM460

HUVECs or NCM460 were cultured in Dulbecco's modified Eagle medium (DMEM) (Gibco, Invitrogen, USA) with 10% fetal bovine serum (FBS), 100 IU mL^−1^ penicillin, and 100 µg mL^−1^ streptomycin, at 37 °C, with humidified air (5% CO_2_).

RAW264.7 cells were cultured in RPIM medium 1640 (Gibco, Invitrogen, USA) with 10% FBS, 100 IU mL^−1^ penicillin, and 100 µg mL^−1^ streptomycin, at 37 °C, with humidified air (5% CO_2_).

3T3‐L1 was cultured in DMEM (Gibco, Invitrogen, USA) with 10% FBS, 100 IU mL^−1^ penicillin, and 100 µg mL^−1^ streptomycin, at 37 °C, with humidified air (5% CO_2_). When the cell density reaches 80–90%, the culture medium is replaced with 3T3‐L1‐induced differentiation medium (PCM‐I‐010, ZQXZBIO, China). After 3 days, the culture medium is replaced with DMEM complete culture medium. When the cells show a mature adipocyte phenotype (more than 80%), they can be used for experiments.^[^ ^74^ ^]^

Control group: DMSO was added to DMEM medium for cell culture and the final concentration of DMSO was 0.1%. ZLY032 treated groups: ZLY032 was dissolved in DMSO and added to DMEM for cell culture, so that the final concentration of DMSO was 0.1% and the final concentration of ZLY032 was 0.5, 5, and 50 µm. VEGF treated group: VEGF was dissolved in PBS (containing 0.1% HSA) and added to DMEM and DMSO was added to this DMEM solution. The final concentration of DMSO was 0.1% and VEGF was 20 ng mL^−1^. LPS treated groups: LPS was dissolved in PBS and added to DMEM, and DMSO was also added to this DMEM solution. The final concentration of DMSO was 0.1% and LPS was 200 ng mL^−1^. RAW264.7 was treated with LPS for 2 h.

High glucose treatment group: in the high‐glucose treatment group, the final concentration of glucose in the culture medium was 30 mm, while in the control group, concentration was 5.5 mm. Experiments were conducted after 72 h of incubation.

### SiRNA Transfection

SiRNA (MingSheng Bio, China) lyophilized powder was diluted with DEPC water. Cells were seeded in advance with a serum concentration of 5%. After 16–18 h of seeding, the cell confluence reached 40–60%. In 200 µL of Opti‐MEM, 3 µL of siRNA (20 µm) was added and mixed five times. In another 200 µL of Opti‐MEM, 6 µL of Lipomax (32105, Sudgen, China) was added and mixed five times. The mixtures were left to stand for 5 min. Then, the two solutions were mixed and left to stand for another 20 min. The resulting mixture was then evenly added to each well and mixed. The cells were incubated in a 37 °C incubator. After 6–8 h of transfection, the medium was replaced with fresh culture medium.

### CCK‐8 Assay to Evaluate Cell Viability

HUVECs were spread in 96‐well plates, cells were treated according to the experimental requirements, 10 µL of CCK‐8 reagent (MA0218‐5, Meilunbio, China) was added to each well, and the absorbance value at 450 nm was detected by an enzyme labeling instrument (Molecular Devices, USA) after incubation for 1–4 h in a 37 °C incubator.

### Scratch Assay

Spread the HUVECs evenly within the 6‐well plate and treat the cells accordingly to the experimental requirements. A 10 µL white sterile tip was used to uniformly scratch the cell surface along the centerline of the culture well plate to form a clear gap. The treated cells were added to the culture medium and put back into the 37 °C constant temperature cell culture incubator for further incubation. At 0, 12, 24, and 36 h, pictures were taken by using an inverted microscope (MA100, Nikon, Japan) to observe the migration distance of the cells, and the scratched area was quantitatively evaluated using ImageJ software.

### Tube Formation Assay

The matrix gel (356237, BD, USA) was spread in a confocal dish and placed in the incubator at 37 °C to solidify for 30 min, and the counted cells were added into the confocal dish with matrix gel according to the specific requirements and conditions of the experiment and incubated at 37 °C for 6 h. The pictures were taken with an inverted microscope after 6 h for observation of the lumen formation of the HUVECs. The results were analyzed and processed with ImageJ.

### RNA Extraction

RNA was extracted from cells by using Trizol method (10296010CN, Invitrogen, USA). And the concentration was measured by NanoDrop ND‐8000 (Thermo Fisher Scientific, Waltham, MA, USA) to ensure that the RNA/DNA value was 1.8–2.0. The ReverTra Ace qPCR RT (FSQ‐101, Toyobo, Japan) kit was applied to reverse transcribe the RNA into cDNA.

*Staphylococcus aureus* suspensions (1 × 10⁸ CFU mL^−1^) were centrifuged at 8000 × *g* for 10 min after 8 h of culture. The bacterial pellets were treated with lysozyme (10 mg mL^−1^, 500 µL; Solarbio, L8120) for cell wall digestion. Total RNA was extracted using the TRIzol method (Invitrogen) according to the manufacturer's protocol. RNA concentration and purity were assessed using a NanoDrop 8000 spectrophotometer (Thermo Fisher Scientific, Waltham, MA, USA), with A260/A280 ratios between 1.8 and 2.0 considered acceptable. Reverse transcription was performed using the ReverTra Ace qPCR RT kit (Toyobo, Japan) to generate cDNA templates for subsequent analysis.

### Quantitative Real‐Time Polymerase Chain Reaction (qRT‐PCR)

Real‐time fluorescence quantitative PCR was performed using SYBR Green PCR (04913914001, Roche, Germany) premixes and primers used for amplification were listed in Table S2 of the Supporting Information. Target genes were quantified on an ABI Real‐time Fluorescence Quantitative PCR System (Applied Biosystems, USA).

### Western Blot

Cell lysate containing 1% PMSF was added to the cells, and protein was extracted. Protein concentration was assayed using the BCA analysis kit (Beyotime, Beijing, China). Equal amounts of protein were subjected to SDS‐PAGE electrophoresis and then transferred onto PVDF membranes (Pall Life Science, Pensacola, FL, USA). After blocking with 5% milk, the membranes were probed with specific antibodies against NF‐κB P65 (10745‐1‐AP, Proteintech, China), p‐NF κB p65 (ab76302, Abcam, USA), and GAPDH (Ab0035, Abways, China). After PBST cleaning, the corresponding secondary antibodies configured with PBS were added, incubated for 1 h. Western blot band images were obtained by using an Oddessy infrared imaging system (LI‐COR Biosciences, Lincoln, NE, USA), and the band densities of each group were quantified using ImageJ software.

### Cellular Thermal Shift Assay (CETSA)

ZLY032 or DMSO‐treated HEK293T cells were collected and equally distributed. Thermal denaturation was performed at different temperatures using a PCR instrument. The cells were then frozen‐thawed with liquid nitrogen for three cycles and centrifuged at 13 500 rpm for 15 min at 4 °C. The obtained supernatants were transferred to new microtubes for Western blotting analysis. Equal amounts of proteins were electrophoresed by SDS‐PAGE and transferred onto PVDF membranes (Pall Life Science, Pensacola, FL, USA). After blocking with 5% milk, the membranes were probed with specific antibodies against ASAL (16645‐1‐AP, Proteintech, China), PPARδ (ab178866, Abcam, USA), FFAR1 (DF8146, Affinity, China), and β‐actin (Ab0035, Abways, China). Western blot band images were obtained by using an Oddessy infrared imaging system (LI‐COR Biosciences, Lincoln, NE, USA), and the band densities of each group were quantified using ImageJ software.

### Enzyme‐Linked Immunosorbent Assay (ELISA)

Arginine and ASAL were detected by using ELISA kits (MY10289, MY10288, Shanghai MeiYi Biotechnology Co., Ltd, China) according to the product specification. The protein samples from MRSA (USA300), *E. coli* (ATCC 43895), *Salmonella* (ATCC 14028s), standards, and HRP‐labeled detection antibody were added into microtiter wells, which were coated with arginine antibody or ASAL antibody. After incubating for 60 min, the well was washed and substrate was added. The absorbance (OD) was measured at 450 nm.

The skin from the wound sites of mice after treating with ZLY032 for three days and five days was collected and lysed with an appropriate amount of lysis buffer (RIPA:PMSF = 100:1). An ELISA kit precoated with anti‐IL‐6 and anti‐IL‐1β antibodies (SEA079Mu; SEA563Mu, Cloud‐clone Corp, China) was used to detect the levels of IL‐6 and IL‐1β.

The skin from the diabetic wound sites of mice after treating with ZLY032 for three days or eight days was collected and lysed with appropriate amount of lysis buffer (RIPA:PMSF = 100:1). An ELISA kit precoated with AGEs antibodies (UCEB353Ge, Cloud‐clone Corp, China) was used to detect the levels of AGEs on day 3 and day 8.

ZLY032 or DMSO treated HUVECs were collected, and an appropriate amount of lysis buffer (RIPA:PMSF = 100:1) was added to the cells for fully lysing. An ELISA kit precoated with anti‐VEGF antibodies (SEA143Hu; Cloud‐clone Corp, China) or anti‐COX‐2 (S0168, Beyotime, China) was used to detect the level of VEGF or COX‐2 in the HUVECs.

### Assessment of MDA and SOD Levels in Wound Tissues

The malondialdehyde (MDA) (A003, Njjcbio, China) and SOD (A001, Njjcbio, China) were measured in diabetic wound tissues. Following the manufacturer's instructions, all indicators were measured using a microplate reader with the appropriate detection kits. The absorbance (OD) was measured at 550 nm (SOD) and 532 nm (MDA).

### RNA Sequencing

The wild strains were incubated in TSB for 16–18 h and treated with ZLY032 or DMSO for 8 h. RNA was extracted using Trizol (10296010CN, Invitrogen, USA), and RNA sequencing was performed using the Illumina HiSeq X platform and the PE‐150 (KSEQHEALTH, China). Differentially expressed genes were analyzed using DEGseq software. Fold‐change >2, *p* < 0.005 indicates differential gene expression between samples.

### Sample Preparation for Scanning Electron Microscopy

The processed *S. aureus* samples were fixed in a refrigerator at 4 °C for more than 1.5 h. Gradient ethanol was used for dehydration. After dehydration, the sample was replaced with tert‐butanol: (100% ethanol:tert‐butanol = 1:1). The sample was put into the freezer at −20 °C for 30 min and then it was put into freeze‐dryer (ES‐2030, HITACHI, Japan) to dry the sample for 4 h, and finally the ion sputtering coater was used to coat the sample surface with a layer of metal film of 100–150 Å. The treated samples were put into the sample box for examination.

### Molecular Docking

To investigate the binding mode of interaction between ZLY032 and ASAL, the protein–ligand docking software Autodock Vina 1.1.2 was used. For docking study, the protein was added polar hydrogen and gasteiger charger, removed water, and assigned atoms as AD4 based on AutoDock Tools. The MM2 function of ChemBio3D Ultra 14.0 was used for energy optimization of the 3D structure of ZLY032 and protein. Docking was operated using full flexibility of ligand, binding pocket parameters were set (center of *x*, *y*, *z* was 31.0, 9.7, 8.8; size of *x*, *y*, *z* was 21.6, 22.4, 15.9) and kept other parameters as default values. Finally, the molecular docking diagrams were edited and exported using Pymol 2.3.1 (Education Edition).

### Molecular Dynamics Simulation

Molecular dynamics simulations of the interaction of ZLY032 with 6IG5 were performed using the GROMACS 2019.6 software package and the Charm36 standpoint, utilizing the most protein–ligand conformation generated by previous molecular docking as the initial structure for molecular dynamics simulations. The topology file of the ligand was generated using CGenFF web server (https://cgenff.umaryland.cedu). For the simulation parameter settings, periodic boundary conditions were used, TIP3P was used as the water model of the system, and cubes were built. Simulations were performed in a solvent environment containing 0.145 m NaCl and a net charge. To ensure sufficient distance between the protein–ligand system and the boundary of the simulation box, the distance between the boundary of the simulation box and the system needed to be greater than the minimum distance of 12 Å. The simulation was performed in a solvent environment containing 0.145 m NaCl and a net charge. Prior to molecular dynamics simulations, each system was subjected to energy minimization using a most rapid descent algorithm. In addition, each system was gradually heated from 0 to 310 K by 100 ps under NVT conditions, and then equilibrated at 1000 ps under NPT and 310 K conditions. Finally, 20 ns was simulated for ASAL‐ZLY032, respectively, during which the results were stored every 2 fs and the outputs were analyzed.

### Antibacterial Activity Determination

The antibacterial activity of compound ZLY032 was evaluated against six species of Gram‐positive bacterias including *S. aureus* (ATCC 6538, ATCC 29213), MRSA (ATCC 43300, USA300), *E. faecalis (*ATCC 29212), and Gram‐negative bacterias including *P. aeruginosa*, *E. coli* (ATCC 43895), and *Salmonella* (ATCC 14028s). *S. aureus* (ATCC 6538) and MRSA (ATCC 43300) were purchased from Guangdong Provincial Microbial Strain Collection Center (GDMCC), and the rest of the bacterial strains were supplied by the Laboratory of Genetic Center of School of Pharmacy, Harbin Medical University.

The bacterial suspension in logarithmic growth phase was diluted to 10^6^ CFU mL^−1^ with MHB medium. The MHB medium containing medicine with different concentrations was mixed with the bacterial suspension and placed in a 96‐well plate. The culture was continued at constant temperature of 37 °C for 24 h. And the OD value was measured at a wavelength of 595 nm on an enzyme labeling instrument (Molecular Devices, USA). The bacterial suspension was diluted tenfold for eight serial times. The diluted bacterial suspension (10 µL) was dropped on LB agar. The agar plates were incubated at 37 °C for 24 h, and the bacterial colonies were counted.

ZLY032 treated group: ZLY032 was dissolved in DMSO, added to MHB medium, and mixed with bacteria‐containing supernatant, the final concentration of DMSO was 0.1%. Penicillin G treated group: Penicillin G was dissolved in PBS and added to MHB medium, and DMSO was added to this medium. Then the medium was mixed with bacteria‐containing supernatant, the final concentration of DMSO was 0.1%. CTRL group: DMSO was added to MHB medium, and mixed with bacteria‐containing supernatant, the final concentration of DMSO was 0.1%.

Specially, ZLY032 treated group (the final concentration of ZLY032 was greater than 100 µm): ZLY032 was dissolved in DMSO and added to MHB medium, making the final concentration of DMSO 0.2%; correspondingly, CTRL (0.2% DMSO) group: DMSO was added to the control group, and the final concentration was 0.2%.

### Minimum Bactericidal Concentration (MBC) Test

The MBC of ZLY032 against *S. aureus* (ATCC6538) and MRSA (USA300) was determined by LB agar plates. *S. aureus* (ATCC6538) or MRSA (USA 300) of logarithmic growth was diluted to 10^6^ CFU mL^−1^ using MHB medium. ZLY032 was added to the bacterial suspension and the final concentrations of ZLY032 were 0, 50, 100, 150, and 200 µm. The bacterial suspension was spread in 96‐well plates and placed in an incubator at 37 °C for 24 h. Each group of bacterial suspension (100 µL) was evenly coated on LB agarose plate and cultured at 37 °C for 24 h. The growth of bacteria on the plate was recorded and the concentration that inhibited the growth of more than 99.99% of the colonies was defined as MBC.

### Bacterial LIVE/DEAD Assay

The bactericidal effect of ZLY032 against MRSA (USA300) was detected by using the LIVE/DEAD Bac Light Bacterial Viability Kit (L7012, Thermo Fisher, Shanghai, China) according to the previous study.^[^ ^75^ ^]^ In brief, bacterial suspensions (10^7^ CFU mL^−1^) were treated with ZLY032 at the final concentrations are 0, 50, and 100 µm, respectively. After incubation for 2 or 12 h, the bacterial suspension was collected and centrifuged at 10 000 × *g* for 5 min. The bacteria was coincubated with PI and SYTO 9 fluorescent dye for an additional 30 min at 37 °C in a dark condition and laser scanning confocal microscopy (Zeiss, LSM800, Germany) was used to observe live and dead bacteria.

### Viable Bacterial Colonies on Plates

The bacterial suspension was diluted to 10^6^ CFU mL^−1^ at logarithmic growth phase, and gradient dilution was performed on it. Then the gradient dilution (10 µL) was added to the LB solid plate, and continued to be incubated for 24 h in the bacterial incubator at 37 °C. The density of the colonies on the plates of different groups was photographed and recorded. A semiquantitative analysis was performed on the colonies in plates.

### The Tube Clarity Test

ZLY032 or DMSO was added to the bacterial suspension (4 mL, 2 × 10^6^ CFU mL^−1^) placing in glass test tubes, and incubated in a bacterial incubator at a constant temperature of 37 °C for 24 h. Photographs were taken to record the clarification of the test tubes of different groups.

### Bacteriostatic Time Curve

The bacterial solutions with different treatments were added into 96‐well plates. The absorbance value at 595 nm was in the range of 0.1–0.2. And the absorbance was recorded from 0 to 8 h cultivation at intervals of 2 h after ZLY032 or DMSO administration in the bacterial solutions. The concentration‐time curves were plotted to analyze the effect of ZLY032 on bacterial inhibition.

### Crystal Violet Staining Assay

Bacteria (10^6^ CFU mL^−1^) containing appropriate concentration of medicine were added to a 24‐well culture plate and incubated in a bacterial incubator at 37 °C for 24–48 h until the bacteria form a mature biofilm. The planktonic bacteria were softly rinsed and removed, then they were placed in 60 °C oven for 15 min, and methanol was added to fix for 15 min. After drying, crystal violet (C8470, Solarbio, China) was used for dyeing for 15 min. Excess staining solution was rinsed and removed and anhydrous ethanol was added to dissolve the crystal violet. The absorbance was detected at 570 nm.

### Flow Cytometry Analysis of the Skin Tissue at the Wound Site

Clean scissors and tweezers were used to obtain skin from the wound sites of mice treated with ZLY032 for three and seven days, it was crushed and it was placed in 15 mL centrifuge tubes, an appropriate amount of trypsin was added at room temperature for 10 min to digest the tissue, and then an equal volume of complete culture medium was added to stop the digestion. This step was repeated three times until the tissue is fully digested. The obtained cells (2300 rpm, 5 min) were centrifuged and they were resuspended in 1× PBS. CD11b (Abs1890030, Absin, China) and CD86 (561963, BD, USA) were respectively incubated at room temperature with the cells obtained from the wound sites on day 3 for 15 min, and CD11b and CD206 (568808, BD, USA) were incubated at room temperature with the cells obtained from the wound sites on day 7 for 15 min. After staining, the cells were centrifuged (2300 rpm, 5 min) and resuspended in 1× PBS. The cells were analyzed by flow cytometer (APOGEE, USA).

### mRNA Stability Assay

The HEK293T cells were transfected with ArgH plasmid, which was derived from *S. aureus* and carries a human promoter (Ldmbio, China). After 24 h post‐transfection, actinomycin D (5 µg mL^−1^, Sigma, USA) and DMSO were added to the control group, and actinomycin D (5 µg mL^−1^, Sigma, USA) and ZLY032 (50 µm) were added to the ZLY032 group. RNA extraction and PCR were performed at 0, 2, 4, 6, and 8 h, respectively, after the addition of actinomycin D and DMSO or ZLY032.

### Surface Plasmon Resonance (SPR)

The SPR experiment was performed according to the previous study.^[^ ^76^ ^]^ Briefly, the CM5 sensor chip (Cat #29149603, Cytiva) was activated for 420 s with the mixture containing 400 mm dichloroethane (EDC) and 100 mm *N*‐hydroxysuccinimide (NHS), at a flow rate of 10 µL min^−1^. ASAL (from *S. aureus*) was diluted to 50 µg mL^−1^ in immobilization buffer, then it was injected to sample channel of BiacoreT200 (Cytiva) at a flow rate of 10 µL min^−1^, and typically result in immobilization levels of 13 200 RU, the reference channel does not need ligand immobilization step. The chip is deactivated by 1 m ethanolamine hydrochloride at a flow rate of 10 µL min^−1^ for 420 s. Compound ZLY032 with the same analyte buffer was diluted to six concentrations (12.5, 6.25, 3.125, 1.5625, 0.78, and 0 µm). Compound ZLY032 was injected to channel Fc1‐ Fc2 at a flow rate of 30 µL min^−1^ for an association phase of 120 s, followed by 300 s dissociation. The association and dissociation process were all handling in the analyte buffer. 6 cycles of analyte were repeated according to analyte concentrations in ascending order.

### Statistical Analysis

Results are expressed as mean ± SD. Data were analyzed using SPSS (version 26.0) and GraphPad Prism 10.0.1 (GraphPad software). Comparisons among multiple groups were analyzed by one‐way analysis of variance (ANOVA) followed by Dunnett's multiple comparison test. Comparisons between the groups were conducted using the Student's *t*‐test. Values of *p* < 0.05 were considered statistically significant.

## Conflict of Interest

The authors declare no conflict of interest.

## Author Contributions

M.G., Z.D., and T.G. contributed equally to this work. L.J., Y.Z., and M.G. conceived and designed the research. Y.W., C.Y., T.G., and Z.D. performed detailed experiments, B.S., Z.L., M.M., W.L., L.C., J.L., T.Z., W.Y., and H.L. assisted in the experiments. M.G., Z.D., Y.D., and Y.W. participated in the analysis of the results. L.J. wrote the entire paper. Z.L., W.W., C.L., H.B., and K.W. prepared the materials and performed the molecular docking. H.B. provided the experimental site. Z.L. synthesized and supplied ZLY032. Y.Z., Y.L., X.L., T.B., and Y.Z. carried out the discussion part. All authors reviewed the manuscript.

## Supporting information



Supporting Information

## Data Availability

The data that support the findings of this study are available on request from the corresponding author. The data are not publicly available due to privacy or ethical restrictions.
