# Supplementary material for: The Multitarget Compound ZLY032 Achieves Treatment of Chronic Wounds
Source: Adv Sci (Weinh). 2025 Jul 17;12(37):e03098. doi: 10.1002/advs.202503098 (PMC12499452; doi:10.1002/advs.202503098)
Supplement: Supplementary file 1 — Supporting Information [file ADVS-12-e03098-s001.docx]

Supporting Information

Title

**The Multi-target Compound ZLY032 Achieves Treatment of Chronic Wounds**

**Authors**

*Manyu Gong ^1,2,A^, Zhiyuan Du ^1,B^, Tiantian Gong ^1,C^, Yu Wang ^1^, Chenchen Yin^1^, Bosi Sun^1^, Zijia Liu^1^, Lianru Chen^3^, Zibin Liao^3^,Wenxin Wang^3^, Tianjiao Zhao^1^, Yifei Wang^1^, Ying Dong^4^ Kexin Wang^1^, Mengru Ma^1^, Weijun Li^1^, Jiacheng Li^1^, Haodong Li^1^, Congcong Lin^5^, Ying Zhang^1^, Yu Liu^1^, Xin Liu^1^, Tao Ban^1^, Hongxia Bao^6,*^, Ying Zhang^1,*^, Yang Zhang^1, *^, Zheng Li^3,*^, Lei Jiao ^1,*^*

**Affiliations**

^1^ Department of Medicinal Chemistry and Natural Medicine Chemistry, Department of Pharmacology (State-Province Key Laboratories of Biomedicine-Pharmaceutics of China, Key Laboratory of Cardiovascular Medicine Research, Ministry of Education), College of Pharmacy, Harbin Medical University, Harbin, Heilongjiang 150081, PR China

^2^ College of Bioinformatics Science and Technology, Harbin Medical University, Harbin 150081, Heilongjiang, China

^3^ School of Pharmacy, Guangdong Pharmaceutical University, Guangzhou 510006, PR China.

^4^ The Second Affiliated Hospital of Harbin Medical University, Harbin, 150081, Heilongjiang, China,

^5^ Department of Pharmaceutics, School of Pharmacy, Harbin Medical University, Harbin, 150081, China

^6^ Genomics Research Center, Key Laboratory of Gut Microbiota and Pharmacogenomics of Heilongjiang Province, State-Province Key Laboratory of Biomedicine-Pharmaceutics of China, College of Pharmacy, Harbin Medical University, Harbin,150081, China.

*^A,B,C^* These authors contributed equally: Manyu Gong, Zhiyuan Du, Tiantian Gong .

* Corresponding authors at: Department of Pharmacology (The State-Province Key Laboratories of Biomedicine-Pharmaceutics of China), Harbin Medical University, 157 Baojian Road, Nangang District, Harbin, Heilongjiang, PR China.

E-mail addresses: 102476@hrbmu.edu.cn (L.Jiao), lizhengdrug@gdpu.edu.cn (Z.Li), [zhangyang0421@hrbmu.edu.cn (Y.Zhang),](mailto:zhangyang0421@hrbmu.edu.cn,) jennying223@hrbmu.edu.cn (Y.Zhang), [Hbao@hrbmu.edu.cn (HX,](mailto:Hbao@hrbmu.edu.cn(HX,) Bao)

**Keywords:** ZLY032, chronic wounds, Argininosuccinate lyase, Antibacterial, Anti-inflammatory


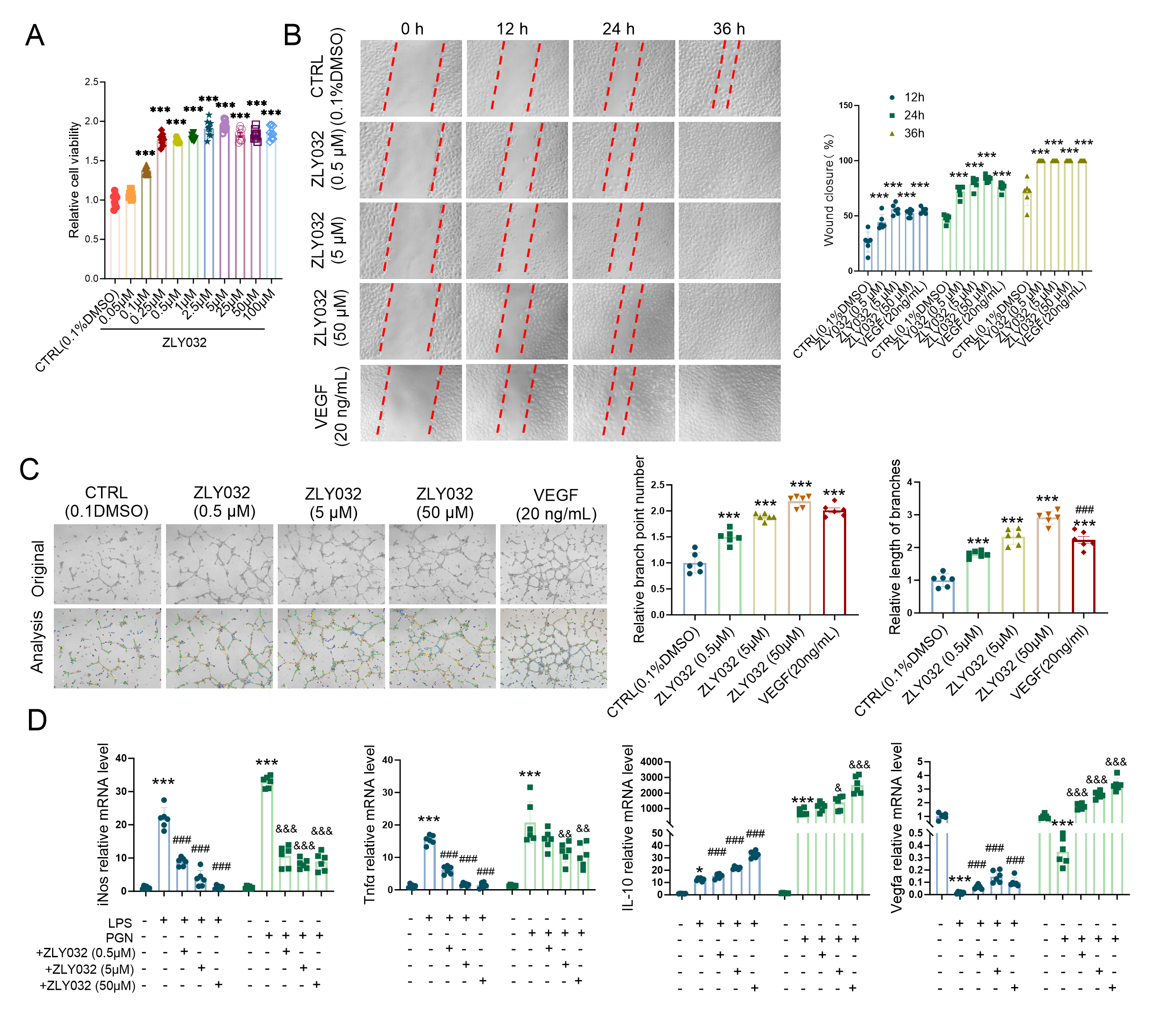


**S1. The effects of ZLY032 on HUVECs and RAW264.7 cells. (A)** The effect of ZLY032 on the activity of HUVECs were detected by CCK8 assay at 0.05 μM, 0.1 μM, 0.25μM, 0.5μM, 1μM, 2.5μM, 5μM, 25μM, 50μM and 100μM concentrations. ****p*<0.001 *vs*. CTRL(0.1%DMSO); n=9 for each group. (Mean ± SD; ordinary one- way ANOVA followed by Tukey's multiple comparisons test among multiple groups). **(B)** Scratch assay to detect the effect of ZLY032 on the migration ability of HUVECs at the concentration of 0.5 μM, 5 μM and 50 μM. ****p*<0.001 *vs*. CTRL(0.1%DMSO); n=6 for each group. (Mean ± SD; two-way ANOVA followed by Tukey's multiple comparisons test among multiple groups). (**C**) Tube formation assay for the effect of ZLY032 on tube formation of HUVECs at the concentration of 0.5 μM, 5 μM and 50 μM. ****p*<0.001 *vs*. CTRL(0.1%DMSO), ^###^*p*<0.001 *vs*. Vegf; n=6 for each group. (Mean ± SD; ordinary one- way ANOVA followed by Tukey's multiple comparisons test among multiple groups). (**D**) qRT-PCR to detect the effects of ZLY032 on the expression of iNos, Tnfα, IL-10 and Vegfa in LPS or PGN stimulated RAW264.7 cells at the concentration of 0.5μM, 5μM and 50μM. **p*<0.05, ****p*<0.001 *vs*. CTRL(0.1%DMSO), ^##^*p*<0.01, ^###^*p*<0.001 *vs.*LPS: LPS(0.1%DMSO), ^&&^*p*<0.01,^&&&^*p*<0.001 *vs.* PGN:PGN(0.1%DMSO); n=6 for each group. (Mean ± SD; ordinary one- way ANOVA followed by Tukey's multiple comparisons test among multiple groups).


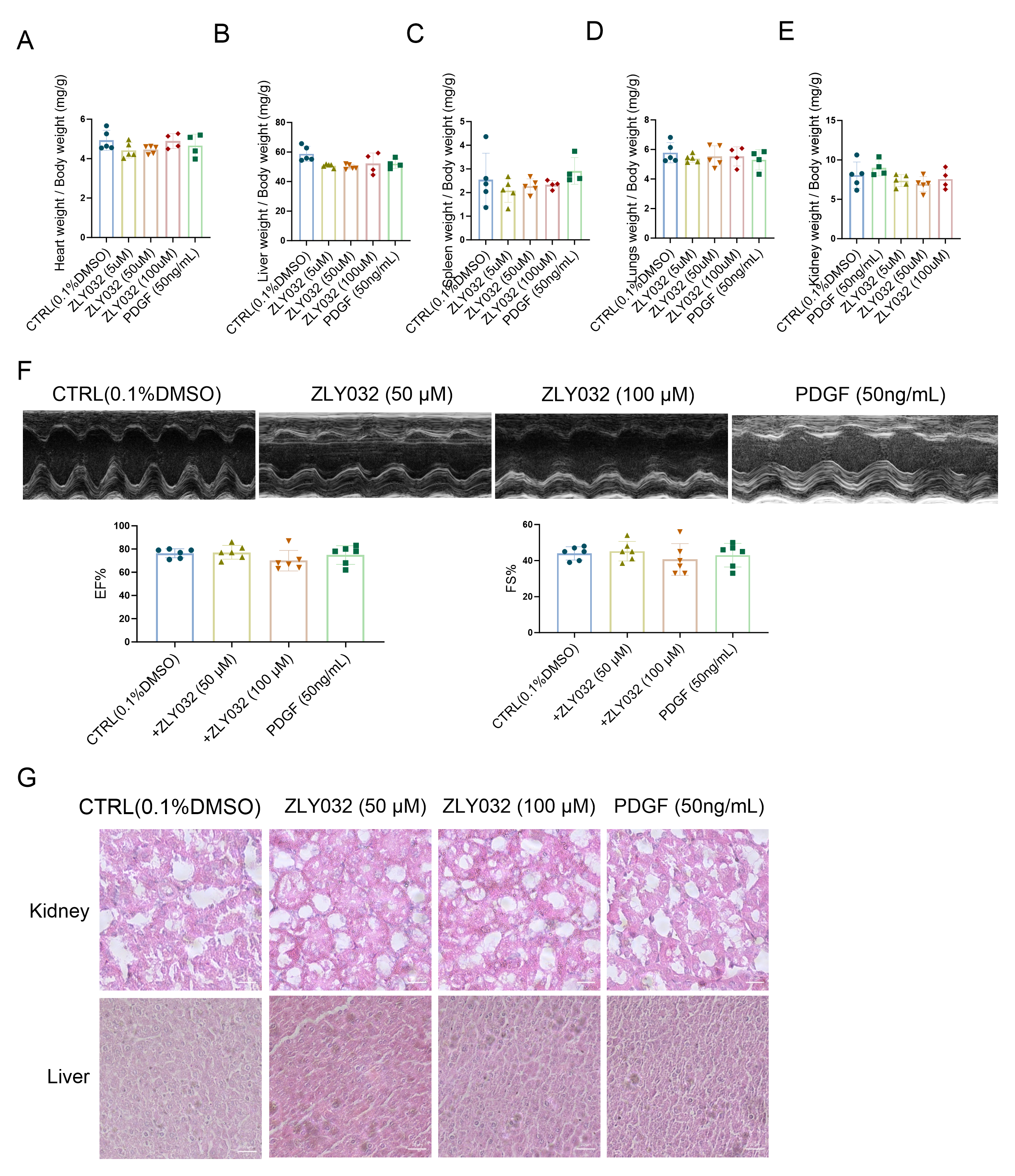


S2. Evaluating the toxicity of ZLY032 in vivo. (A-E) Measurement of heart weight, liver weight, spleen weight, lung weight and kidney weight to body weight ratios under the treatment of 5μM, 50μM and 100μM concentrations. n=4-6 for each group. (F) Up panel :Echocardiographic detection of the effects of ZLY032 on cardiac function in mice treated with MIC and 2MIC concentrations.PDGF was acted as positive control.Down panel: statistical results of left ventricular ejection fraction and shortening of the short axis of the left ventricle of the heart in mice. n=6 for each group. (G) HE staining to observe the morphological changes of kidney and liver under the treatment of ZLY032. (Mean ± SD; ordinary one- way ANOVA followed by Tukey's multiple comparisons test among multiple groups).


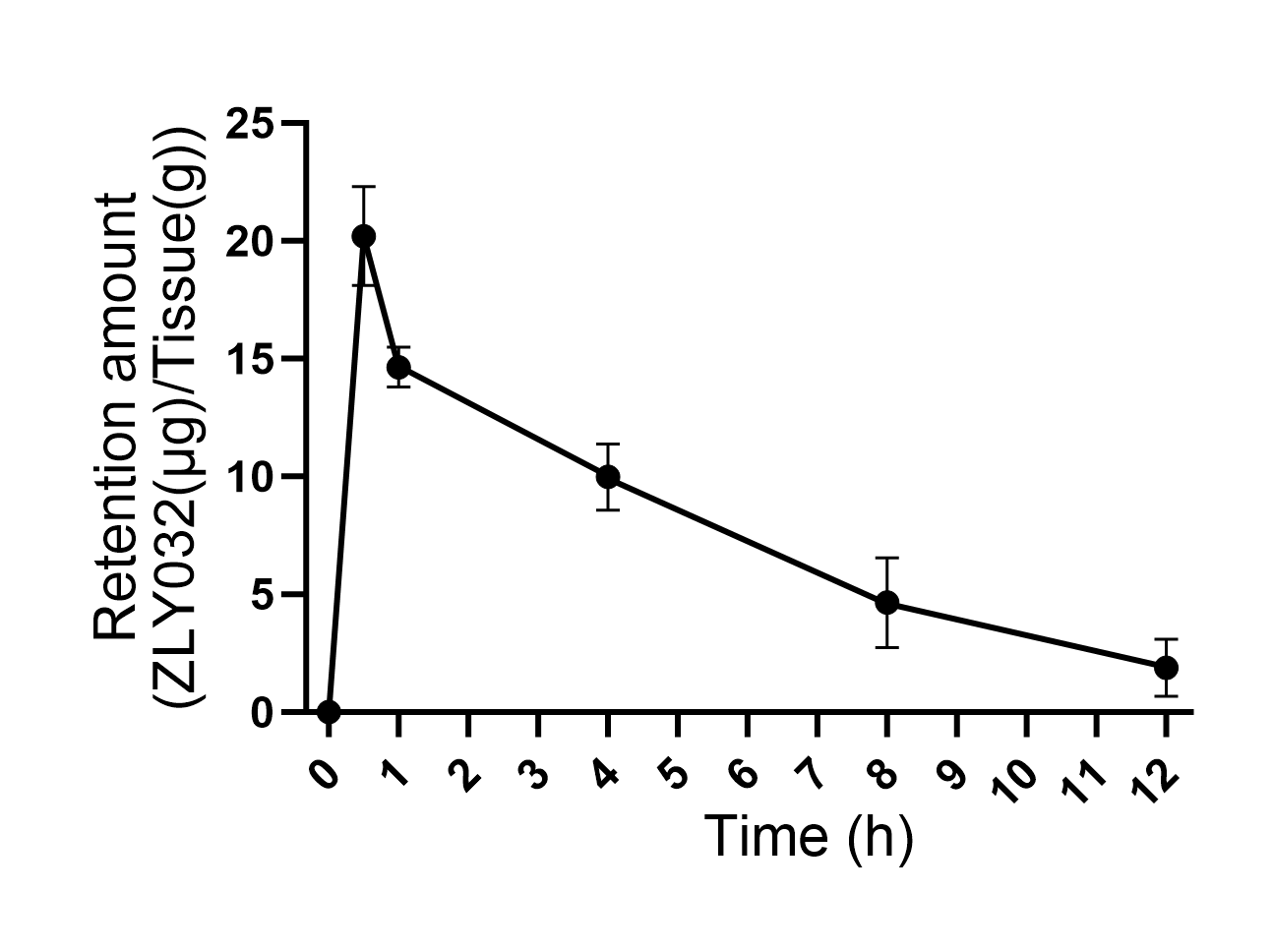


S3. Retention amount of ZLY032 in the wound tissue of mice. LC-MS to detect the rerention of ZLY032 in the wound tissue after topical administration of ZLY032 (100 μM, 10 μL) for 0h, 0.5h, 1h, 4h, 8h and 12h.


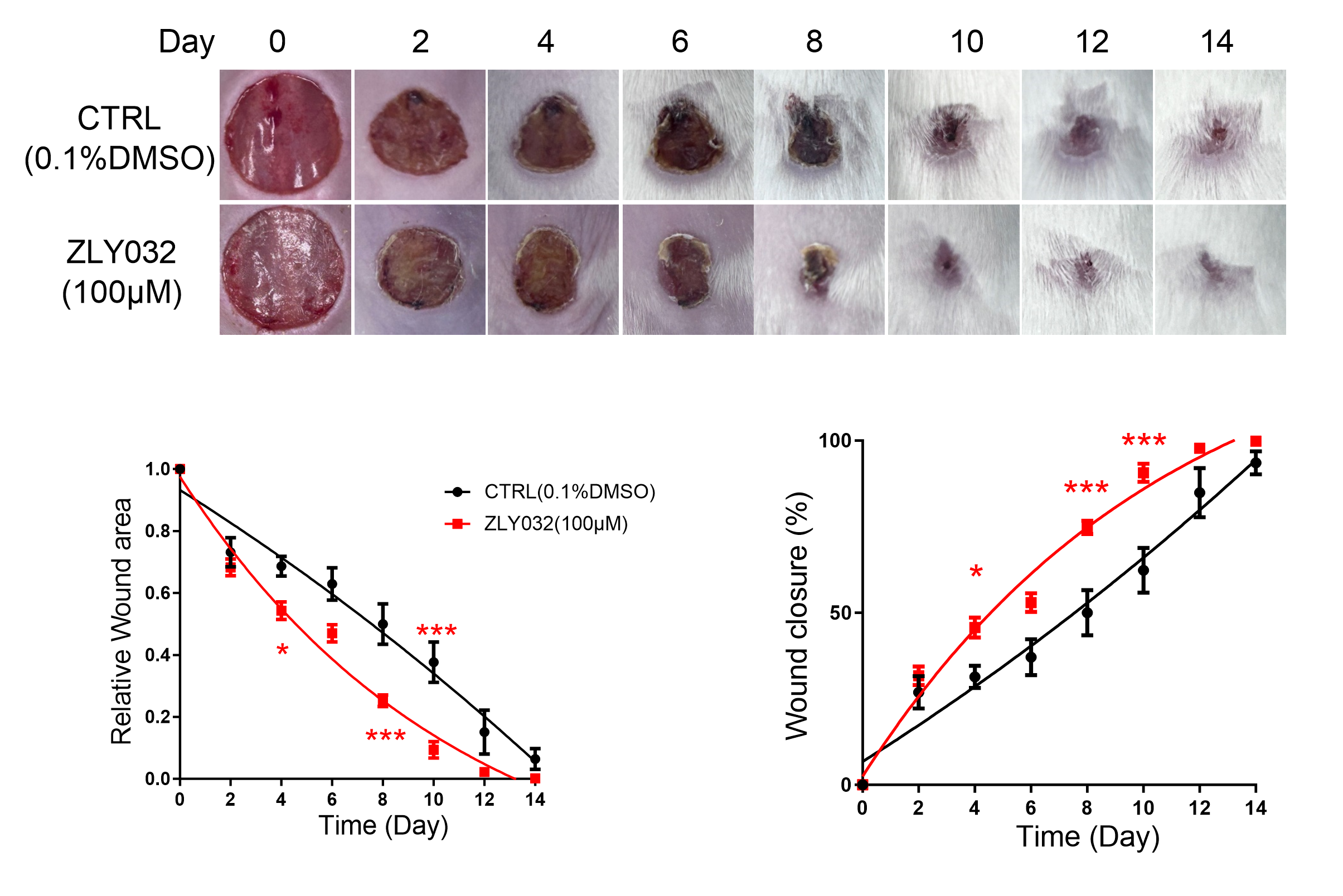


S4. Effect of ZLY032 on wound healing in female mice. Up panel: representative photographs showing the time-dependent closure of wounds in female mice and the wound healing-promoting effect of ZLY032. Down panel: the wound area and wound closure rate of each mice at varying time points. **p<*0.05, ****p*<0.001 vs. CTRL(0.1%DMSO); n=6 for each group. (Mean ± SD; two-way ANOVA followed by Tukey's multiple comparisons test among multiple groups).

**
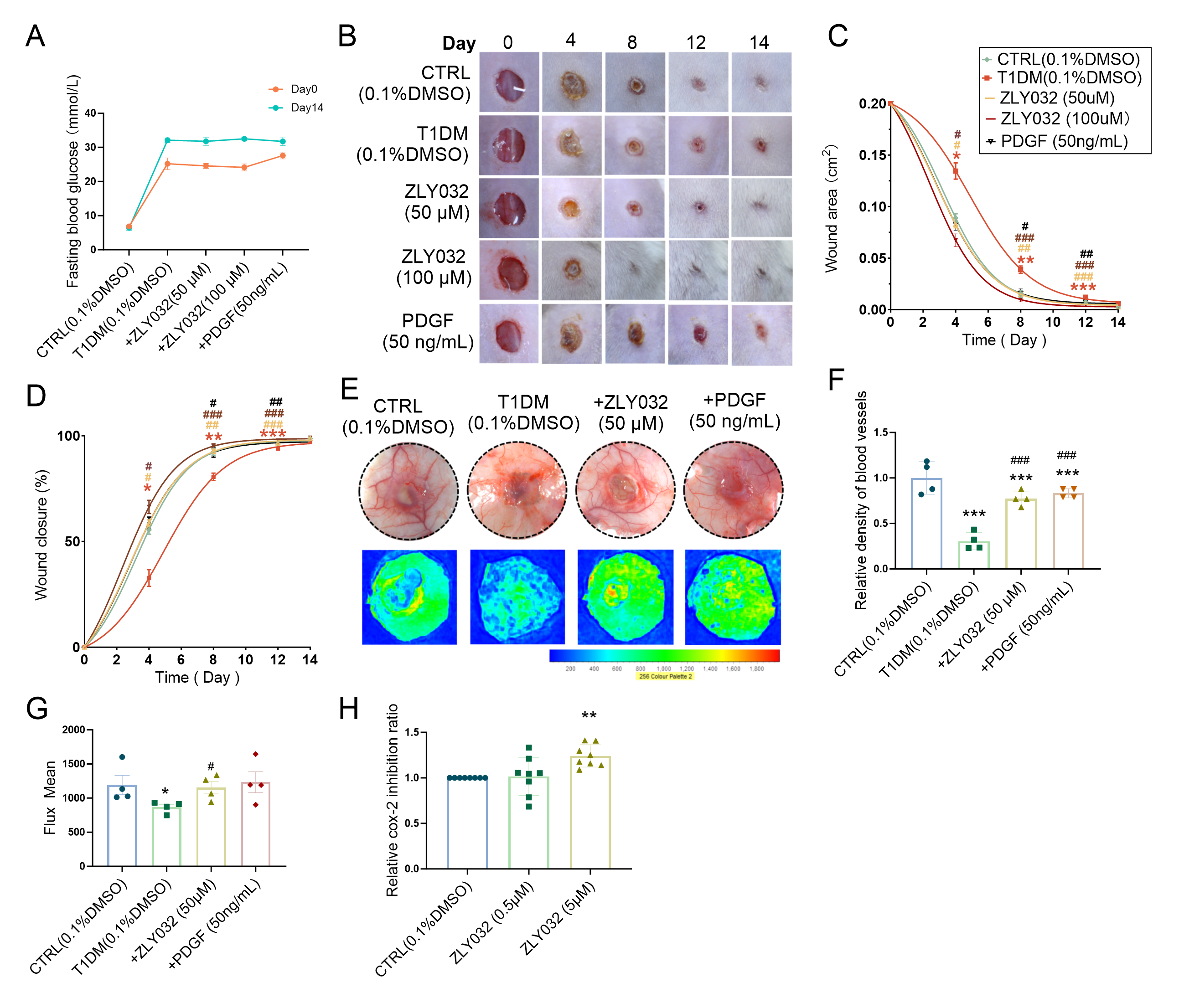
**

S5. Effect of ZLY032 on wound healing in a mouse model of type 1 diabetes mellitus (T1DM). (A) Blood glucose assay in T1DM model mice. (B) Representative photographs showing the time-dependent closure of wounds in T1DM mice and the wound healing-promoting effect of ZLY032. PDGF was acted as positive control. **(C-D)** The wound area and wound closure rate of each mice at varying time points. **p<*0.05, ***p*<0.01, ***p*<0.01 vs. T1DM(0.1%DMSO); *^#^p*<0.05,*^##^p*<0.01,*^###^p*<0.001 vs. PDGF; n=6 for each group.(Mean ± SD; two- way ANOVA followed by Tukey's multiple comparisons test among multiple group) (E-G) In the T1DM model, blood flow at the wound site was measured by using a Doppler flow detector on day 8 after administration of ZLY032 and PDGF. ****p*<0.001 *vs*. CTRL(0.1%DMSO), *^###^p*<0.001 *vs*. T1DM ; n=4 for each group. (H) Cox-2 inhibitor screening kit to detect the pain inhibitory effect of ZLY032 at 0.5 μM and 5 μM concentrations.***p*<0.01 *vs*. CTRL(0.1%DMSO); n=8 for each group. (Mean ± SD ordinary one- way ANOVA followed by Tukey's multiple comparisons test among multiple groups).


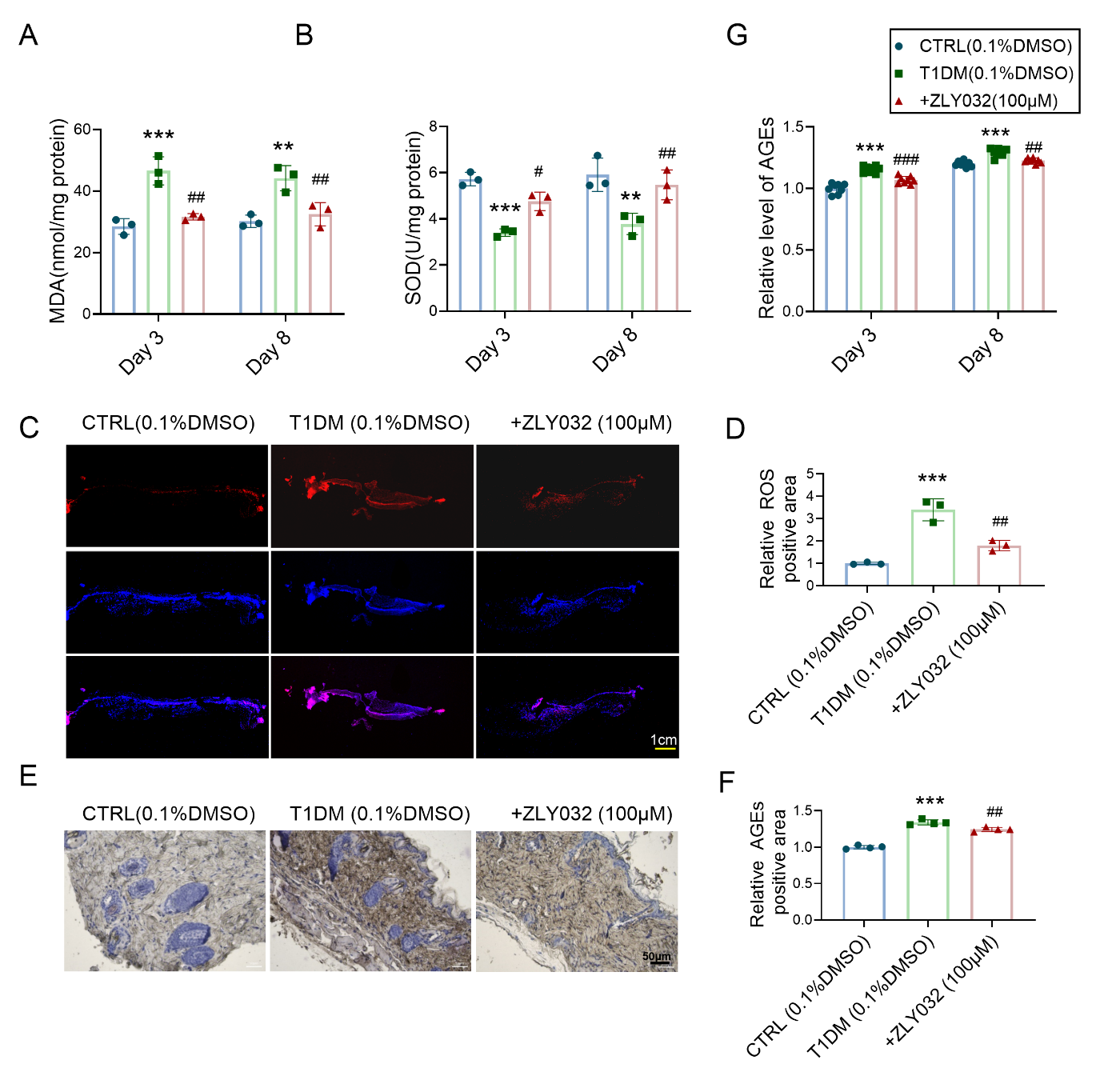


**S6. Effect of ZLY032 on the metabolic microenvironment at the wound site**. **(A-B)** ELISA analysis to evaluate the malondialdehyde (MDA) and superoxide dismutase (SOD) levels in wound tissues at both 3 and 8 days. ***p*<0.05,****p*<0.001 *vs.* CTRL(0.1% DMSO); ^#^*p*<0.05, ^##^*p*<0.01, ^###^*p*<0.001*vs.* T1DM (0.1% DMSO) ; n=6 for each group. (Mean ± SD; ordinary one- way ANOVA followed by Tukey's multiple comparisons test among multiple groups). **(C-D)** Immunofluorescence to evaluate the reactive oxygen species (ROS) levels in day 8 wound tissue. ****p*<0.001 *vs.* CTRL(0.1% DMSO); ^##^*p*<0.01 *vs.* T1DM (0.1% DMSO); n=3 for each group. (Mean ± SD; ordinary one- way ANOVA followed by Tukey's multiple comparisons test among multiple groups). **(E-F)** Immunohistochemical (IHC) to detecte the advanced glycation end products (AGEs) levels in day 8 wound tissue. ****p*<0.001 *vs.* CTRL(0.1% DMSO); ^##^*p*<0.01 *vs.* T1DM (0.1% DMSO); n=4 for each group. (Mean ± SD; ordinary one- way ANOVA followed by Tukey's multiple comparisons test among multiple groups). (G) ELISA kits to detect the AGEs levels in the wound tissue at both 3 and 8 days. *** *p*<0.001 *vs.* CTRL(0.1% DMSO); ^###^*p*<0.001, *vs.* T1DM (0.1% DMSO) ; n=6 for each group. (Mean ± SD; two-way ANOVA followed by Tukey's multiple comparisons test among multiple groups)


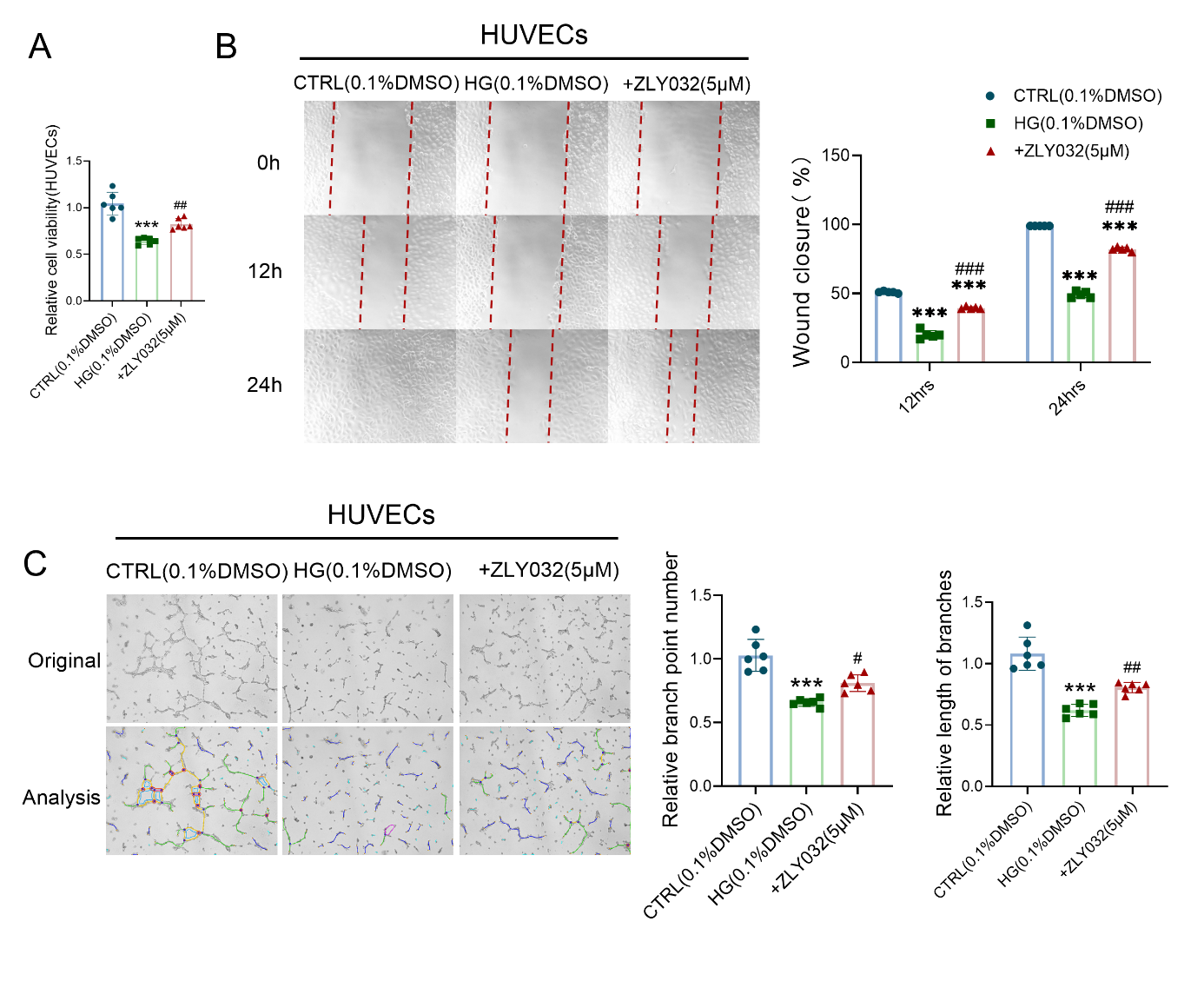


**S7. Effect of ZLY032 on the proliferation, migration, and tube formation of high-glucose-treated HUVECs.** **(A)** CCK-8 assay to detect the effect of ZLY032 on proliferation of high-glucose-treated HUVECs. *** *p*<0.001 vs CTRL(0.1% DMSO); ^##^*p*<0.01, vs HG (0.1% DMSO) ;n=6 for each group. (Mean ± SD; ordinary one- way ANOVA followed by Tukey's multiple comparisons test among multiple groups). **(B)** Scratch assay to evaluate the effect of ZLY032 on migration of high-glucose-treated HUVECs. *** *p*<0.001 vs CTRL(0.1% DMSO); ^###^*p*<0.001, vs HG (0.1% DMSO) ;n=6 for each group. (Mean ± SD; two-way ANOVA followed by Tukey's multiple comparisons test among multiple groups). **(C)** Tube formation assay to evaluate the effect of ZLY032 on tube formation in high-glucose-treated HUVECs. *** *p*<0.001 vs CTRL(0.1% DMSO); ^#^*p*<0.05, ^##^*p*<0.01 vs HG (0.1% DMSO) ;n=6 for each group. (Mean ± SD; ordinary one- way ANOVA followed by Tukey's multiple comparisons test among multiple groups)


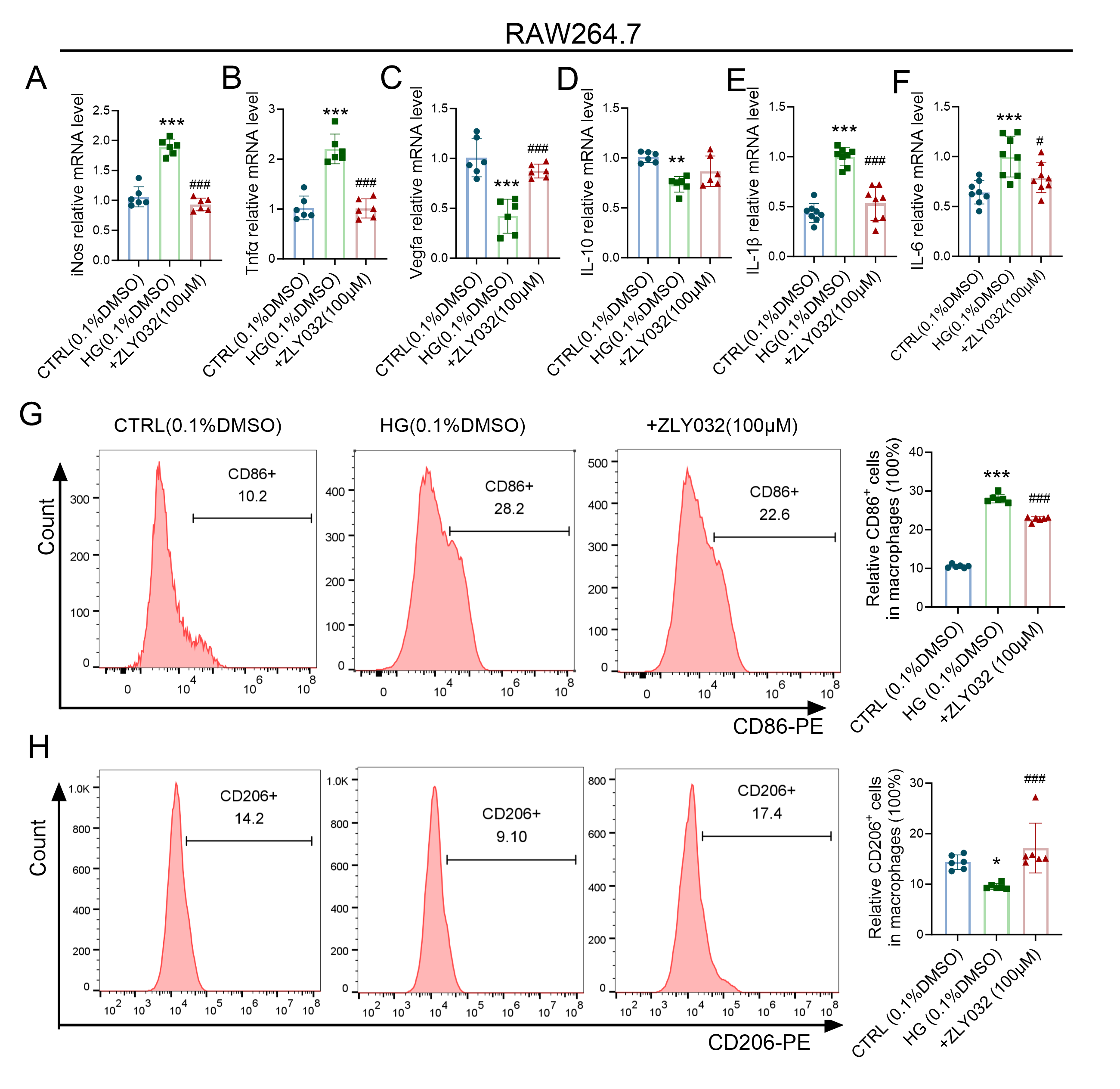


**S8. Effect of ZLY032 on high glucose treated RAW264.7 cells. (A-F)** qRT-PCR analysis of the effect of ZLY032 on the expression of iNos, Tnfα, IL-10, Vegfa, IL-6 and IL-1β in high-glucose-treated RAW264.7 cells. ***p*<0.01,****p*<0.001 vs. CTRL(0.1%DMSO), ^#^*p*<0.05, ^###^*p*<0.001 *vs.* HG(0.1%DMSO). (Mean ± SD; ordinary one- way ANOVA followed by Tukey's multiple comparisons test among multiple groups) **(G-H)** Flow cytometry analysis of ZLY032's effects on CD86^+^ or CD206^+^ macrophages in high-glucose-treated RAW264.7 cells. **p*<0.05,****p*<0.001 vs. CTRL(0.1%DMSO), ^###^*p*<0.01 *vs.* HG(0.1%DMSO); n=6 for each group. (Mean ± SD; ordinary one- way ANOVA followed by Tukey's multiple comparisons test among multiple groups).


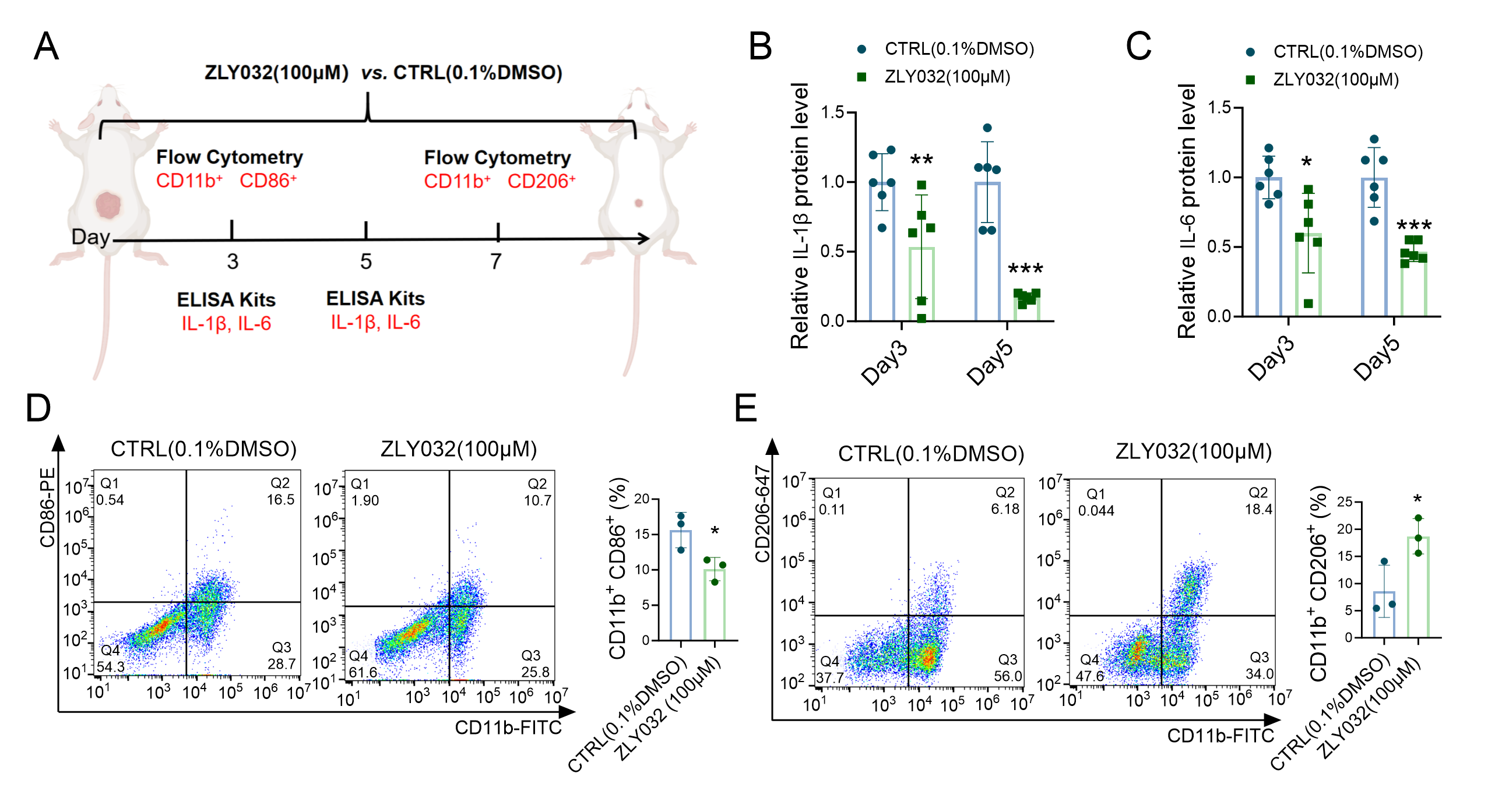


**S9. The effect of ZLY032 on inflammatory factors and macrophages at the wound site.** **(A)** Flow diagram. **(B-C)** ELISA kits to detect the expression level of IL-1β and IL-6 in the wound tissue on the 3rd and 5th day after ZLY032 (50 μM) administration. **p*<0.05, ***p*<0.01, ****p*<0.001 *vs*. CTRL(0.1%DMSO), n=6. (Mean ± SD; Student *t*-test for comparisons between two groups). **(D)** Flow cytometry to determine the amount of CD11b^+^ CD86^+^ macrophages at the wound site at day 3 after administration of ZLY032. **p<*0.05, n=3. (Mean ± SD; Student *t*-test for comparisons between two groups). **(E)** Flow cytometry to determine the amount of CD11b^+^ and CD206^+^ macrophages at the wound site on day 7 after administration of ZLY032. **p<*0.05, n=3. (Mean ± SD; Student *t*-test for comparisons between two groups).


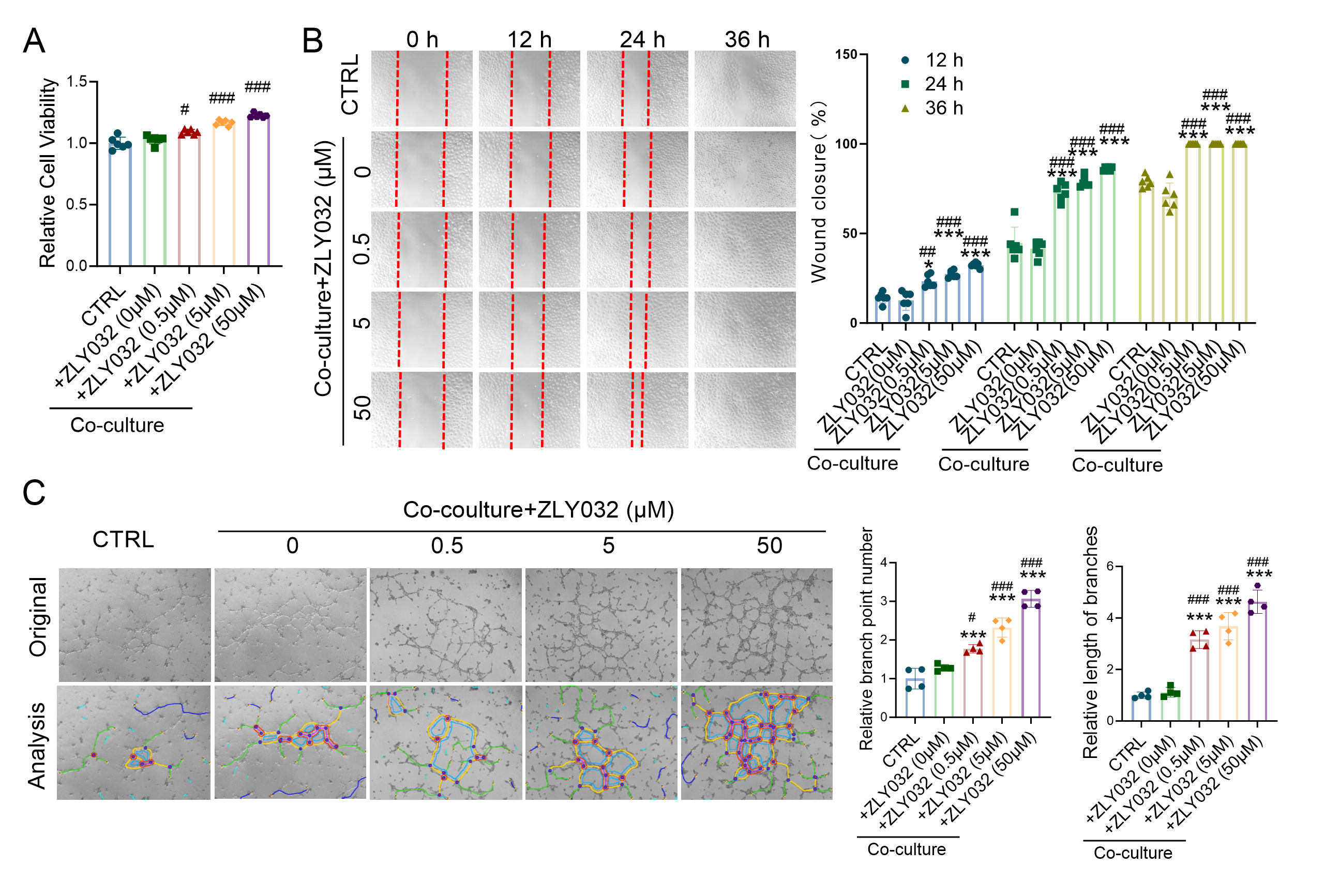


S10. ZLY032 indirectly promotes angiogenesis by modulating macrophages. (A) Macrophages (RAW264.7) treated with ZLY032 (0μM, 0.5μM, 5μM, 50μM) were co-cultured with HUVECs and the proliferation of HUVECs was verified by CCK8. ^#^*p*<0.05, ^###^*p*<0.001 *vs*. +ZLY032(0 μM); n=6. (Mean ± SD; ordinary one- way ANOVA followed by Tukey's multiple comparisons test among multiple groups). (B) The migration of HUVECs was tested by Scratch Assay, which were co-cultured with RAW264.7 cells treating with ZLY032 (0μM, 0.5μM, 5μM, 50μM). **p*<0.05,****p*<0.001 *vs*. CTRL;^##^*p*<0.01, ^###^*p*<0.001 *vs*. +ZLY032 n=6. (Mean ± SD; two-way ANOVA followed by Tukey's multiple comparisons test among multiple groups). (C) The tube formation of HUVECs was detected, which were co-cultured with ZLY032 (0μM, 0.5μM, 5μM, 50μM) treated macrophages (RAW264.7). ****p*<0.001 *vs*. CTRL;^#^*p*<0.05, ^###^*p*<0.001 *vs*. +ZLY032 ; n=4.(Mean ± SD; ordinary one- way ANOVA followed by Tukey's multiple comparisons test among multiple groups).


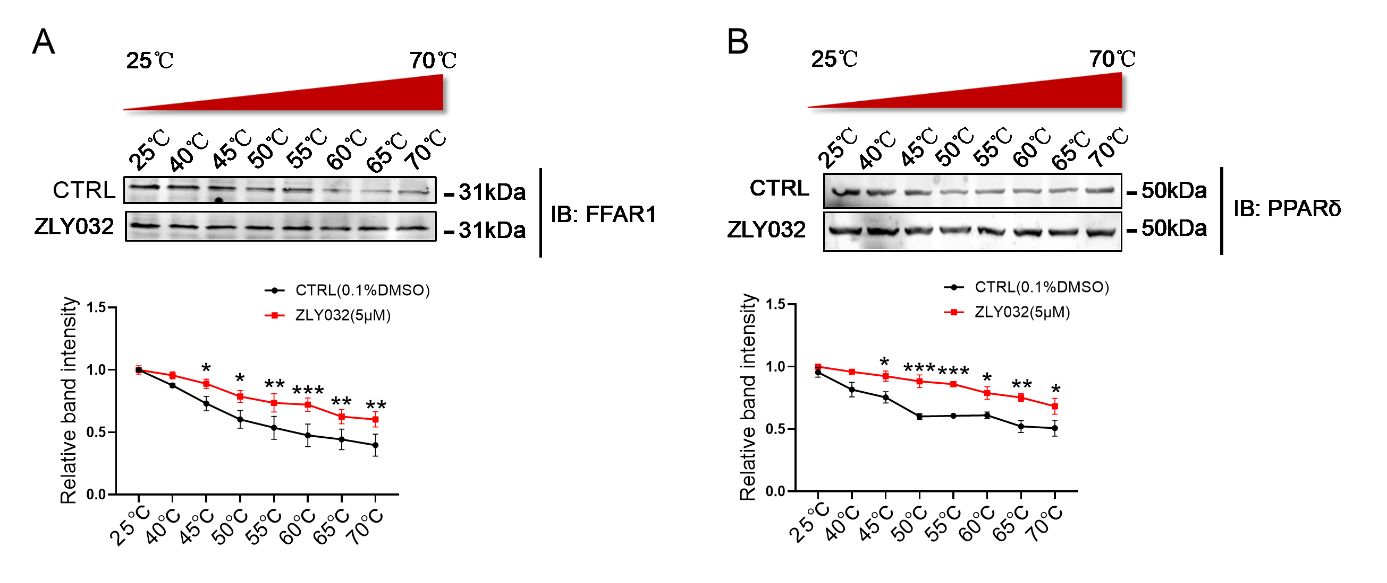


**S11. The binding of ZLY032 to FFA1 and PPARδ. (A-B)** Cellular thermal shift assays (CESTA) to detect the direct binding of ZLY032 on FFA1 and PPARδ. ***p*<0.01, ****p*<0.001 vs. CTRL(0.1%DMSO); n=3 for each group. (Mean ± SD; Student *t*-test for comparisons between two groups).


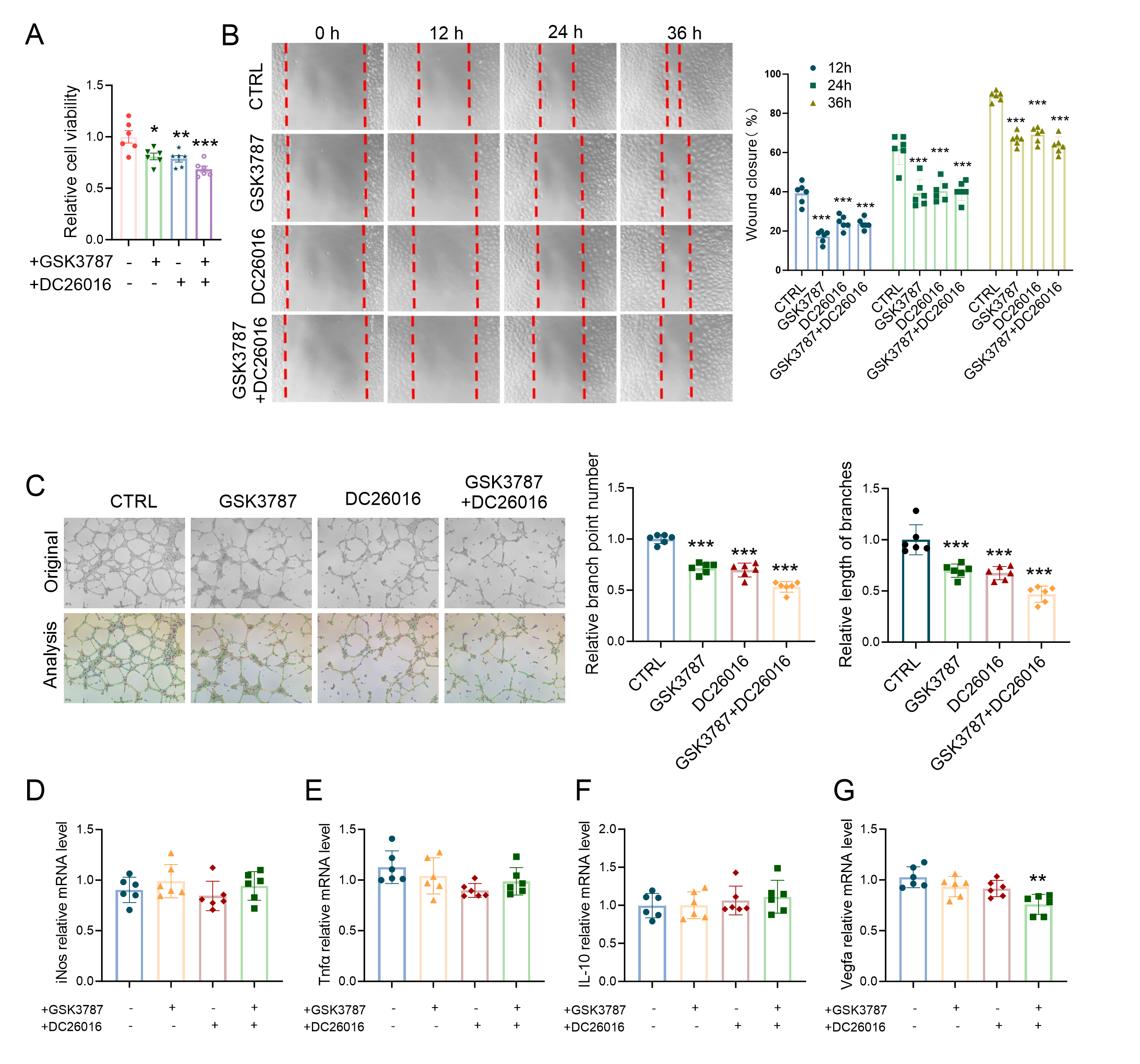


S12. The effects of GSK3787 and DC26016 on HUVECs and RAW264.7 cells. (A-C) The effects of GSK3787, DC26016 and GSK3787 and DC26016 together on the proliferation, migration (Mean ± SD; ordinary one- way ANOVA followed by Tukey's multiple comparisons test among multiple groups), tube formation of HUVECs. **p*<0.05, ***p*<0.01, ****p*<0.001 *vs.* CTRL; n=6 for each group. (D-G) The effects of GSK3787, DC26016 and GSK3787 and DC26016 together on the expression of iNos, Tnfα, IL-10 and Vegfa in RAW264.7 cells. ***p*<0.01 *vs.* CTRL; n=6. (Mean ± SD; ordinary one- way ANOVA followed by Tukey's multiple comparisons test among multiple groups)


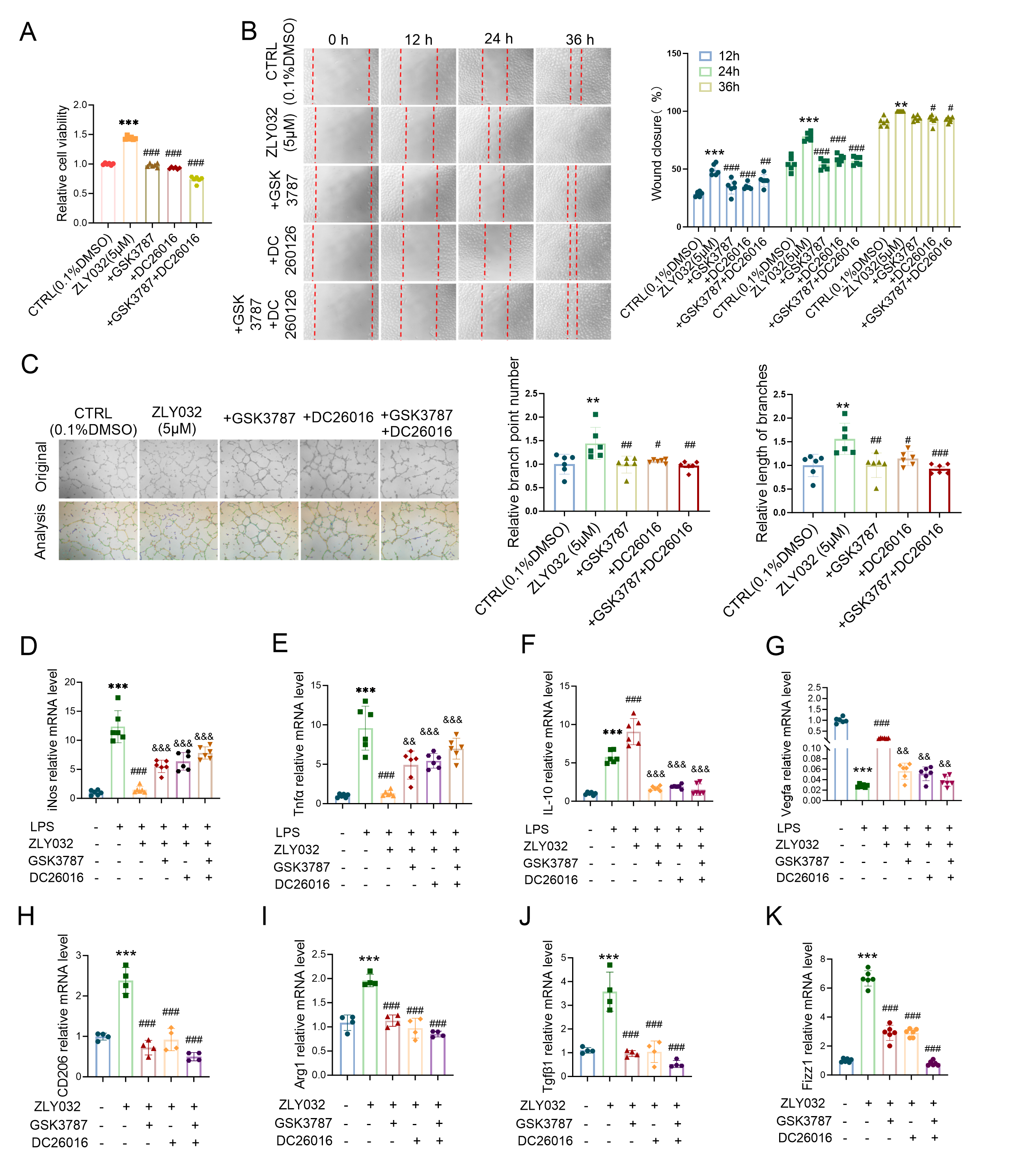
**S13. ZLY032 promotes angiogenesis and exerts anti-inflammatory effects by activating PPARδ and FFA1. (A)** Effect of ZLY032 (5 μM) in combination with the PPARδ inhibitor GSK3787 (0.5 μM) and the FFA1 inhibitor DC26016 (10 μM) on HUVEC activity as detected by CCK8 assay. ****p*<0.001 *vs*. CTRL(0.1%DMSO), ^###^*p*<0.001 *vs.*ZLY032(5 μM); n=6 for each group. (Mean ± SD; ordinary one- way ANOVA followed by Tukey's multiple comparisons test among multiple groups). **(B)** Wound healing assay was performed to detect the effect of ZLY032 (5 μM) on the migratory capacity of HUVECs after combination with the PPARδ inhibitor GSK3787 (0.5 μM) as well as the FFA1 inhibitor DC26016 (10 μM). ***p*<0.01,****p*<0.001 *vs*. CTRL(0.1%DMSO); ^#^*p*<0.05,^##^*p*<0.01,^###^*p*<0.001 *vs.*ZLY032(5 μM); n=6 for each group. (Mean ± SD; two-way ANOVA followed by Tukey's multiple comparisons test among multiple groups). **(C)** Tube formation assay to detect the effect of ZLY032 (5 μM) in combination with the PPARδ inhibitor GSK3787 (0.5 μM) and the FFA1 inhibitor DC26016 (10 μM) on the tube formation ability of HUVEC. ***p*<0.01 *vs*. CTRL(0.1%DMSO). ^#^*p*<0.05, ^##^*p*<0.01, ^###^*p*<0.001 *vs.*ZLY032(5 μM); n=6 for each group. (Mean ± SD; ordinary one- way ANOVA followed by Tukey's multiple comparisons test among multiple groups). **(D-G)** qRT-PCR was used to detect the effects of ZLY032 (5 μM) in combination with the PPARδ inhibitor GSK3787 (0.5 μM) and the FFA1 inhibitor DC26016 (10 μM) on the expression of iNos, Tnfα, IL-10 and Vegfa in LPS stimulated RAW264.7 cells. ****p*<0.001 *vs*. CTRL(0.1%DMSO), ^###^*p*<0.001 *vs.* LPS, ^&&^*p*<0.01, ^&&&^*p*<0.001 *vs.* ZLY032(5 μM); n=6 for each group. (Mean ± SD; ordinary one- way ANOVA followed by Tukey's multiple comparisons test among multiple groups). **(H-K)** qRT-PCR to detect the effects of PPARδ inhibitor GSK3787 (0.5 μM) and the FFA1 inhibitor DC26016 (10 μM) on the expression of CD206, Arg1, Tgfβ1 and Fizz1 in the ZLY032 (5 μM) treated RAW264.7 cells. ****p*<0.001 *vs*. CTRL(0.1%DMSO), ^###^*p*<0.001 *vs.* ZLY032(5 μM); n=4-6 for each group. (Mean ± SD; ordinary one- way ANOVA followed by Tukey's multiple comparisons test among multiple groups).


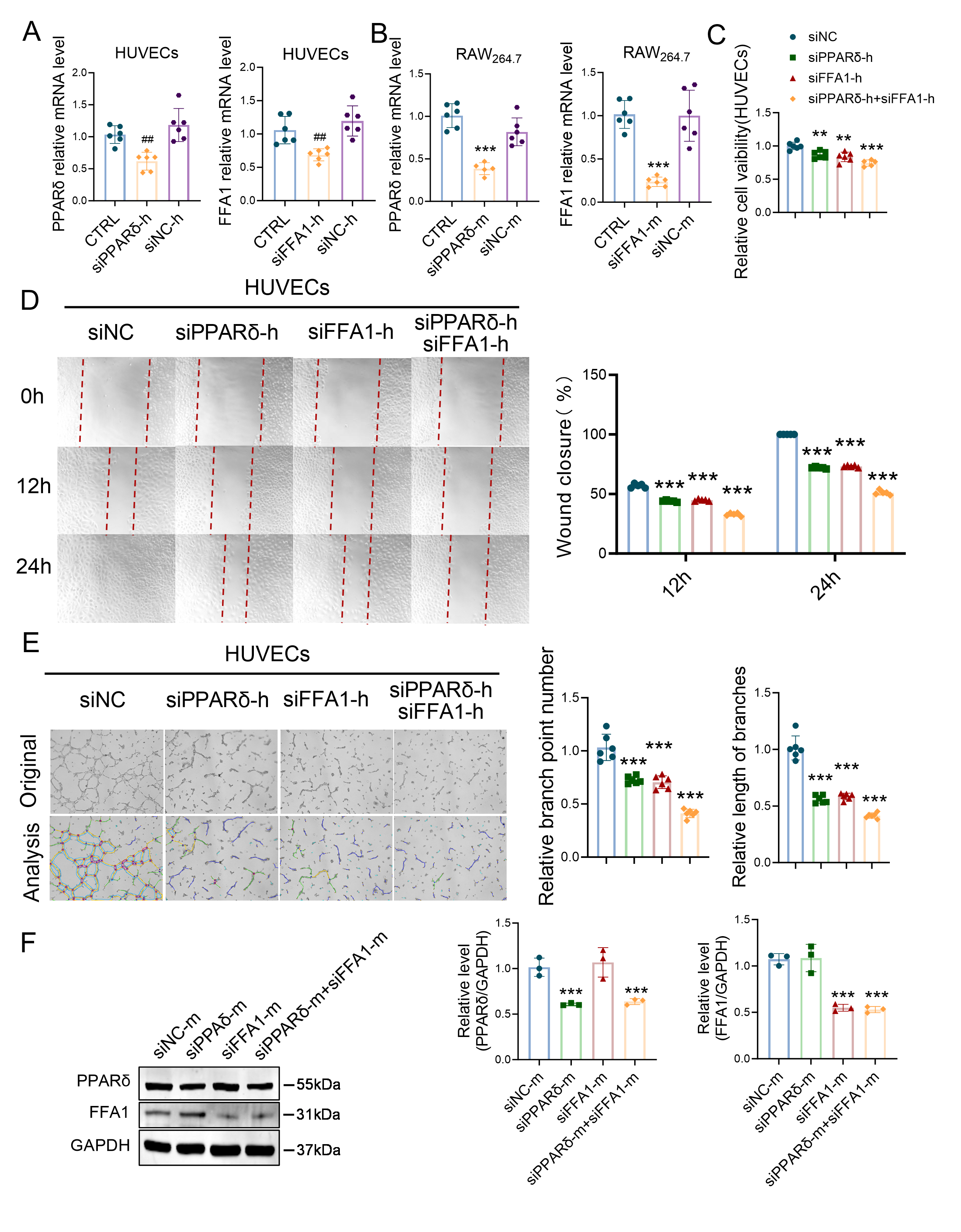


**S14.** **Effects of PPARδ and FFA1 knockdown on HUVECs.** **(A-B)** Knockdown efficiency of siRNAs was validated in HUVECs and RAW264.7 cells by qRT-PCR. ^##^*p*<0.01*vs.* siNC-h ***p*<0.01 *vs*. siNC-m. ; n=6 for each group. (Mean ± SD; ordinary one- way ANOVA followed by Tukey's multiple comparisons test among multiple groups). **(C)** CCK-8 assay for proliferation in HUVECs following single or combined knockdown of *PPARδ* and *FFA1*. ***p*<0.01, ****p*<0.001 *vs*. siNC-h ; n=6 for each group. (Mean ± SD; ordinary one- way ANOVA followed by Tukey's multiple comparisons test among multiple groups). **(D)** Scratch assay for migration in HUVECs following single or combined knockdown of PPARδ and FFA1. ****p*<0.001 *vs*. siNC-h ; n=6 for each group. (Mean ± SD; two-way ANOVA followed by Tukey's multiple comparisons test among multiple groups). **(E)** Tube formation assay for tube formation ability in HUVECs following single or combined knockdown of *PPARδ* and *FFA1.* ****p*<0.001 *vs*. siNC-h ; n=6 for each group. (Mean ± SD; ordinary one- way ANOVA followed by Tukey's multiple comparisons test among multiple groups). **(F)** Western blot was employed to evaluate the knockdown efficiency of siRNAs in skin tissue. ****p*<0.001 *vs*. siNC-m ; n=3 for each group. (Mean ± SD; ordinary one- way ANOVA followed by Tukey's multiple comparisons test among multiple groups).


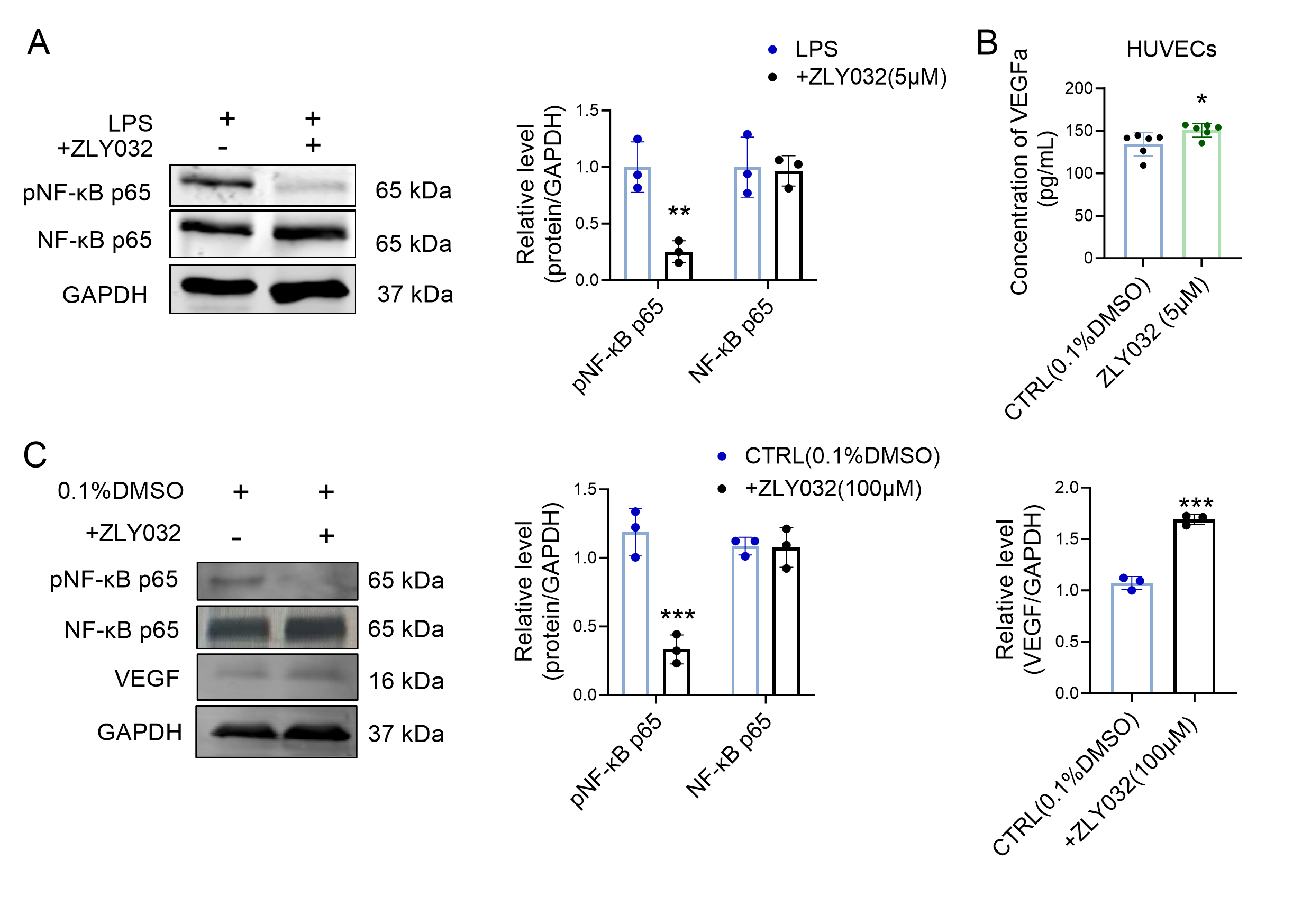


**S15.** **The Effects of ZLY032 on wound healing via FFA1/PPARδ/NF-κB and FFA1/PPARδ/VEGF pathways.** **(A)**The effect of ZLY032 on pNF-κB p65 and NF-κB p65 in LPS-treated RAW264.7 cells were detected by Western blot. ***p*<0.01 *vs.* LPS; n=3 for each group. (Mean ± SD; Student *t*-test for comparisons between two groups). **(B)** The effect of ZLY032 on the expression of VEGF in HUVECs was detected by ELISA. **p*<0.05 *vs.* CTRL(0.1%DMSO); n=6 for each group. (Mean ± SD; Student *t*-test for comparisons between two groups). **(C-D)**Western blot analysis of p-NF-κB p65, total NF-κB p65, and VEGF expression in wound tissues from drug-treated (ZLY032) and untreated mice. ****p*<0.001 *vs.*CTRL(0.1%DMSO); n=3 for each group. (Mean ± SD; Student *t*-test for comparisons between two groups).





S16. The effects of ZLY032 on induced 3T3-L1 cells. (A-E) The expression level of Fgf, Igf1r, Adipor1, IL-1β and IL-6 in induced 3T3-L1 cells evaluated by qRT-PCR. **p*<0.05, ****p*<0.001 *vs.* CTRL(0.1%DMSO); n=6 for each group. (Mean ± SD; Student *t*-test for comparisons between two groups).


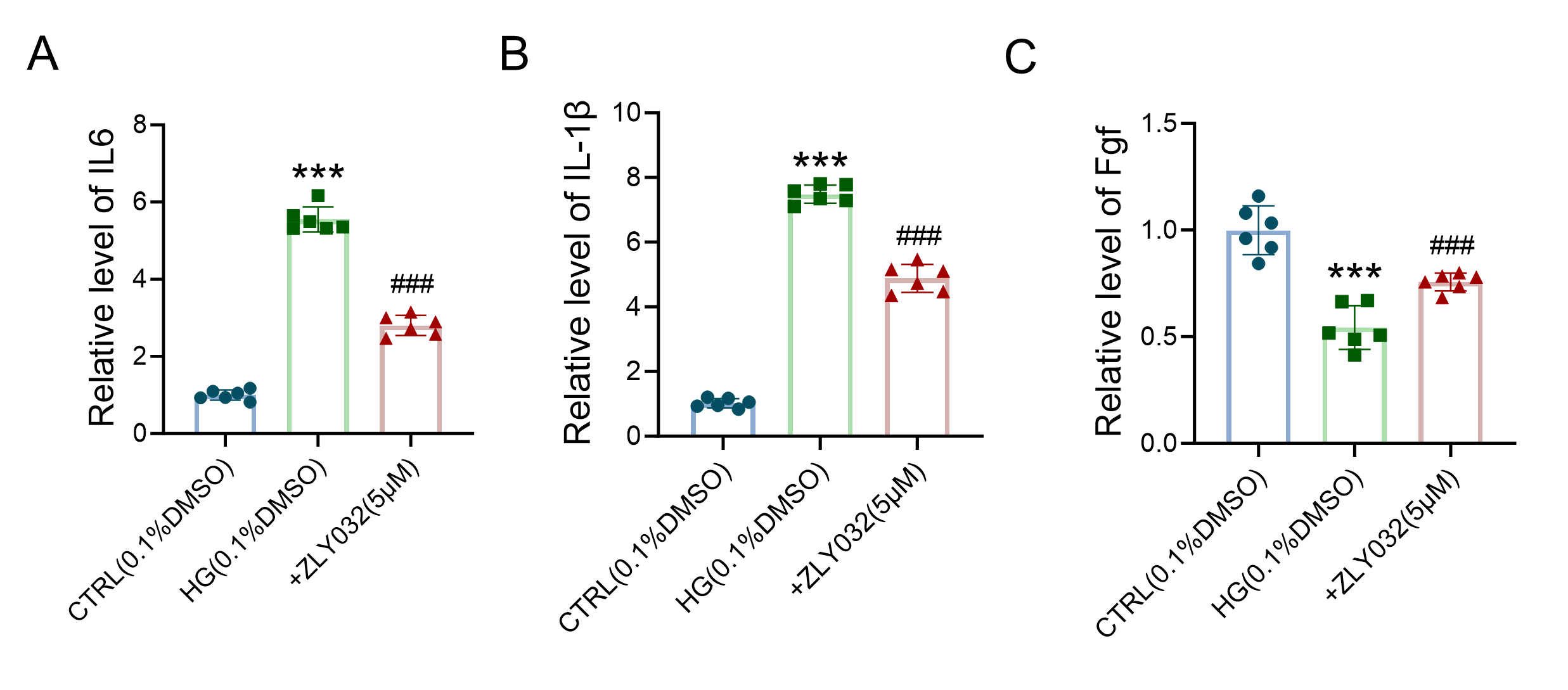


**S17. Effects of ZLY032 on high glucose-treated 3T3-L1 adipocytes.** **(A-C)** The expression level of IL-6, IL-1β and Fgf in high-glucose-treated induced-3T3-L1 cells was evaluated by qRT-PCR. ****p*<0.001(0.1%DMSO) *vs.* CTRL, ^###^*p*<0.001 *vs.* HG(0.1%DMSO); n=6 for each group. (Mean ± SD; ordinary one- way ANOVA followed by Tukey's multiple comparisons test among multiple groups).


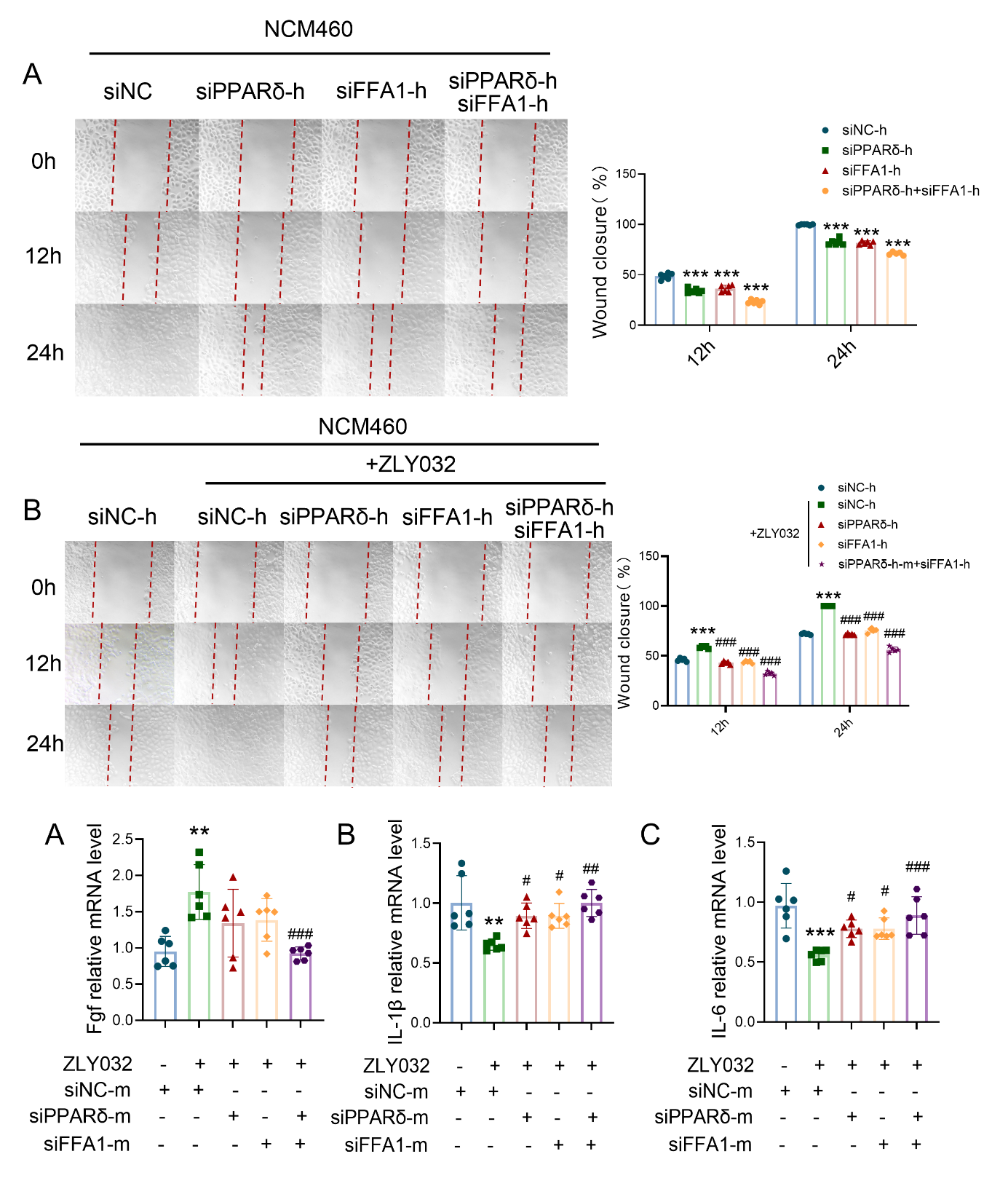


**S18. Effect of PPARδ and FFA1 knockdown on ZLY032-treated 3T3-L1 adipocytes. (A-C)** The expression level of Fgf, IL-1β and IL-6 in induced 3T3-L1 cells following single or combined knockdown of PPARδ and FFA1 evaluated by qRT-PCR. ***p*<0.01,****p*<0.001 *vs.* siNC-m; *^#^p*<0.05, *^##^p*<0.01,*^###^p*<0.001 *vs.* siNC-h+ZLY032; n=6 for each group. (Mean ± SD; ordinary one- way ANOVA followed by Tukey's multiple comparisons test among multiple groups).


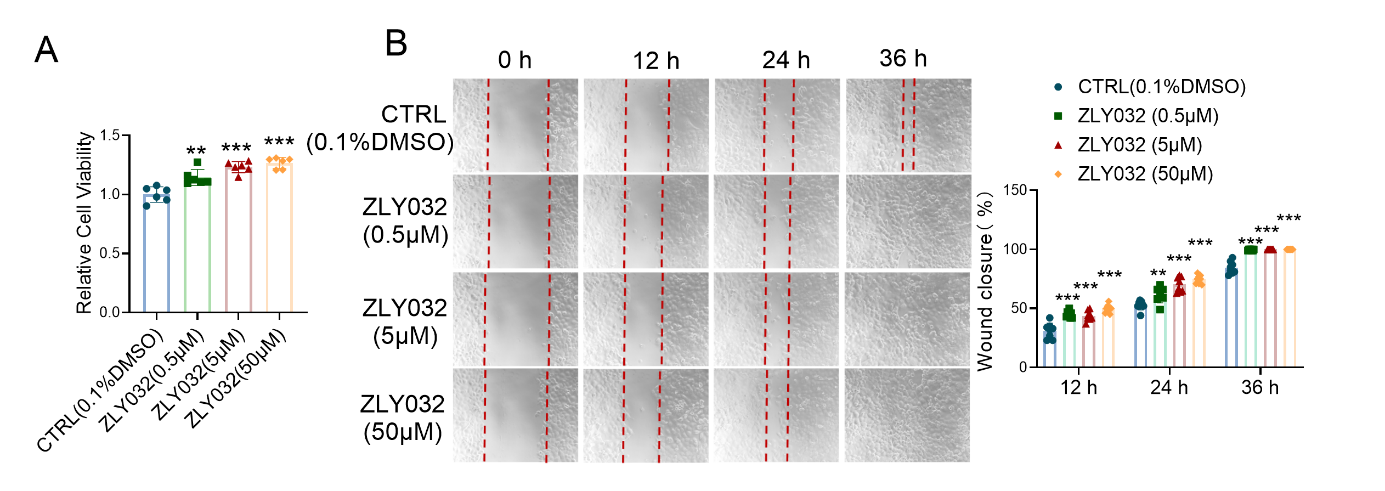


**S19. The effect of ZLY032 on epithelial cells (NCM460)**. **(A-B)** The effect of ZLY032 (0.5μM, 5μM, 50 μM) on the proliferation (Mean ± SD; ordinary one- way ANOVA followed by Tukey's multiple comparisons test among multiple groups) and migration of NCM460 cells was detected by CCK8 assay and scratch assay. ***p*<0.01, ****p*<0.001 *vs.* CTRL(0.1%DMSO); n=6 for each group. (Mean ± SD; two-way ANOVA followed by Tukey's multiple comparisons test among multiple groups).


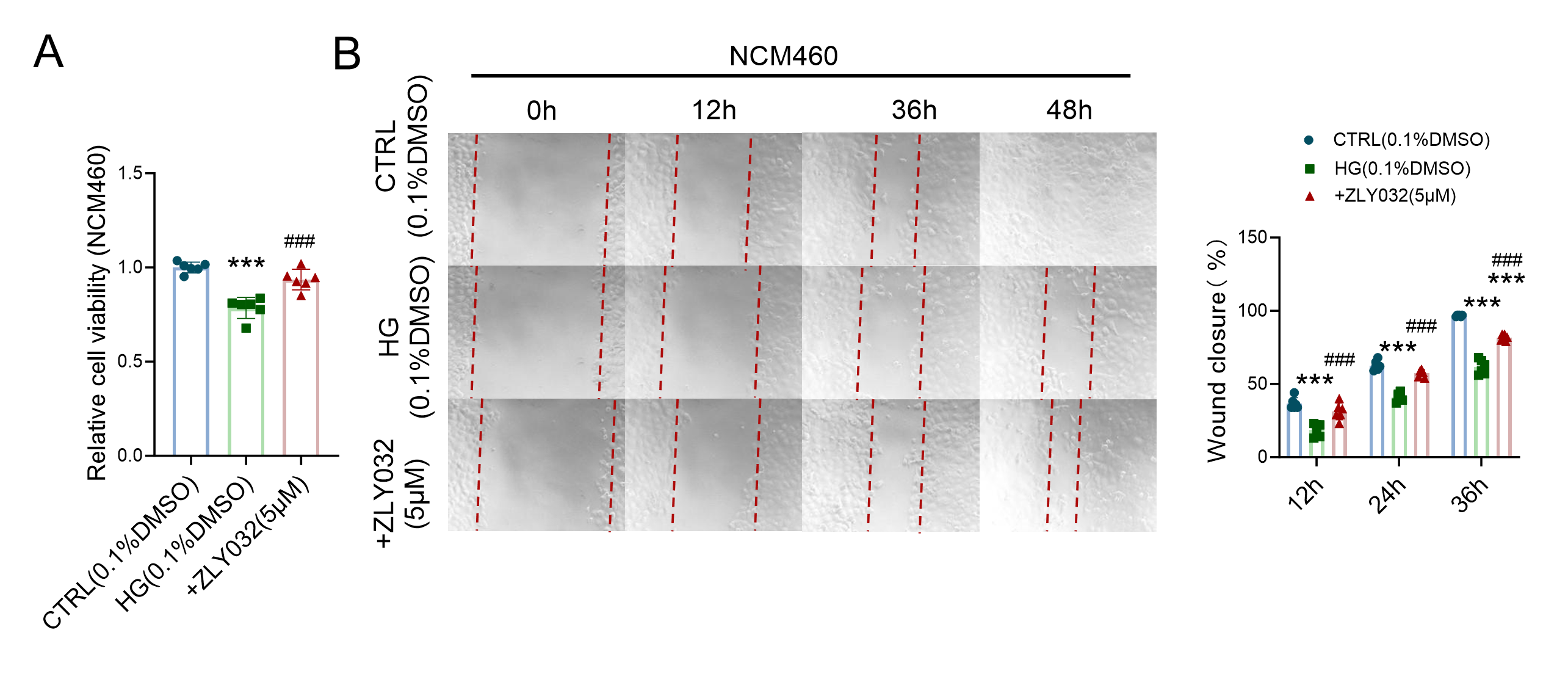


**S20. Effects of ZLY032 on high-glucose-treated NCM460 cells. (A-B)** The effect of ZLY032 on the proliferation (Mean ± SD; ordinary one- way ANOVA followed by Tukey's multiple comparisons test among multiple groups) and migration of NCM460 cells was detected by CCK8 assay and scratch assay. ****p*<0.001 *vs.* CTRL(0.1%DMSO); *^###^p*<0.001 *vs.* HG(0.1%DMSO) ;n=6 for each group. (Mean ± SD; two-way ANOVA followed by Tukey's multiple comparisons test among multiple groups).


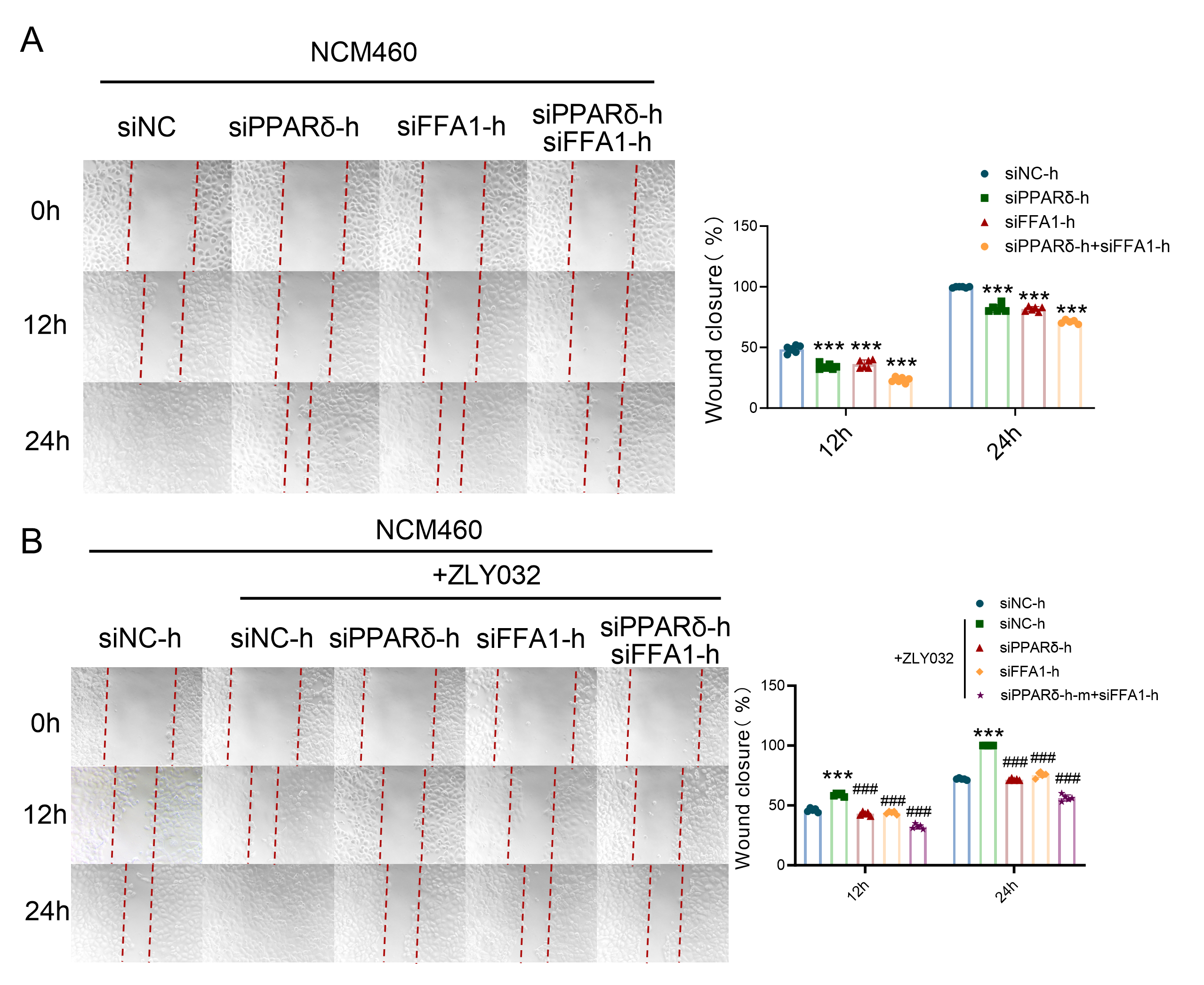


**S21. Effect of PPARδ and FFA1 knockdown on NCM460 cells. (A-B)** Scratch assay for migration in ZLY032 untreated and treated NCM460 cells following single or combined knockdown of PPARδ and FFA1. ****p*<0.001 *vs*. siNC-h ; ***^###^****p*<0.001 vs. siNC-h +ZLY032(5μM) ;n=6 for each group. (Mean ± SD; two-way ANOVA followed by Tukey's multiple comparisons test among multiple groups).


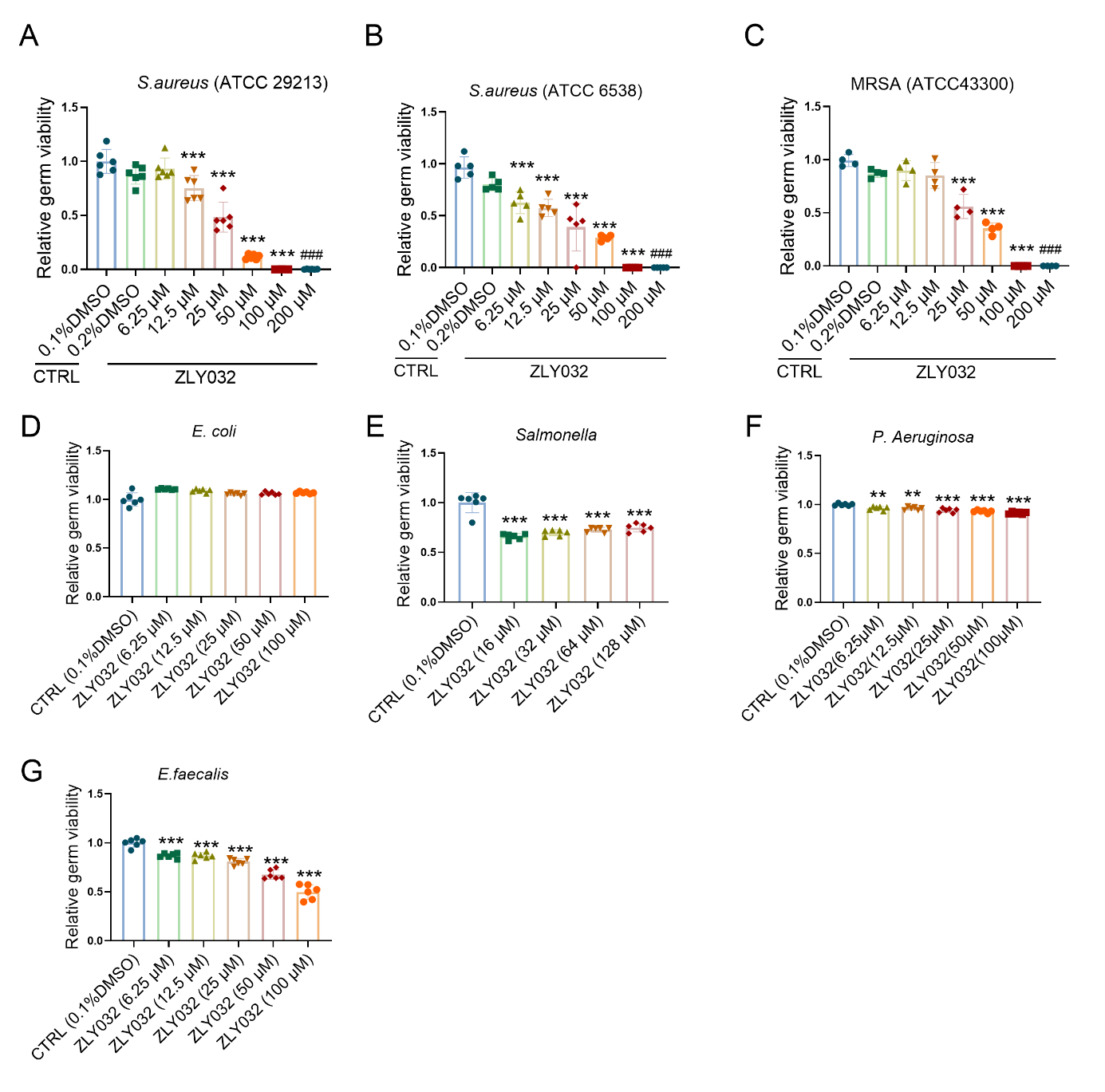


S22. Inhibitory effect of ZLY032 on Gram-negative bacteria. (A-C) The inhibition effects of ZLY032 on *S. aureus* (ATCC 29213, ATCC 6538) and MRSA (ATCC 43300) at the concentrations of 6.25μM, 12.5μM, 25μM, 50μM, 100μM and 200μM. **p*<0.05, ***p*<0.01, ****p*<0.001 *vs*. CTRL(0.1%DMSO), ^###^*p*<0.001 vs. CTRL(0.2%DMSO); n=4-6 for each group. (D) The effect of ZLY032 on E.coli at the concentrations of 6.25μM, 12.5μM,25μM, 50μM and 100μM. n=6 for each group. (E)The effects of ZLY032 on Salmonella at the concentrations of 16 μM, 32 μM, 64μM and 128 μM, ****p*<0.001 *vs.* CTRL(0.1%DMSO), n=6 for each group. (F)The effects of ZLY032 on P.Aeruginosa at the concentrations of 6.25μM, 12.5μM,25μM, 50μM and 100μM. ***p*<0.01 ,****p*<0.001vs. CTRL(0.1%DMSO); n=6 for each group. (G)The effects of ZLY032 on E.faecalis at the concentrations of 6.25μM, 12.5μM, 25μM, 50μM and 100μM. ****p*<0.001 *vs.* CTRL(0.1%DMSO); n=6 for each group. (Mean ± SD; ordinary one- way ANOVA followed by Tukey's multiple comparisons test among multiple groups).


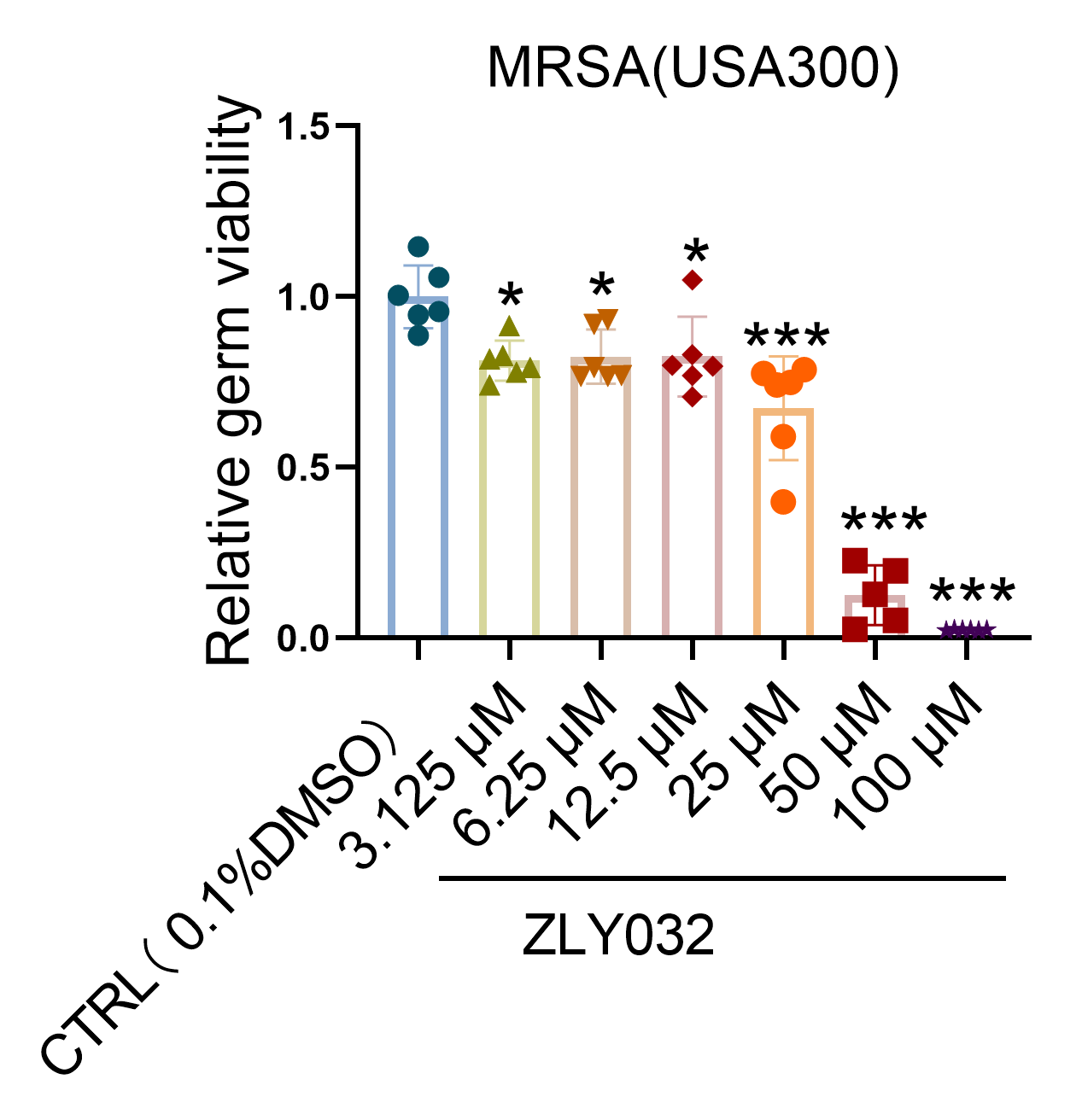


**S23. Evaluation of the anti-bacterial effects of ZLY032 against MRSA (USA300)**. **p*<0.05, ****p*<0.001 vs. CTRL(0.1%DMSO); n=6 for each group.(Mean ± SD; ordinary one- way ANOVA followed by Tukey's multiple comparisons test among multiple groups).

**
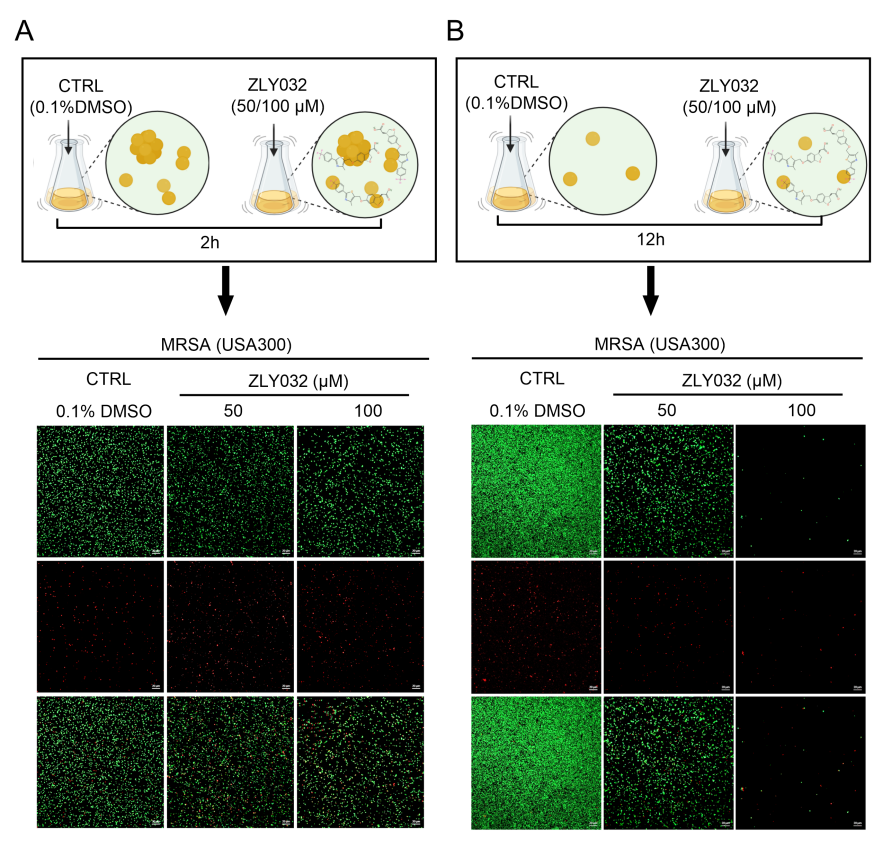
**

**S24. LIVE/DEAD assay to evaluate the effects of ZLY032 on MRSA (USA300). (A-B)** Green represents complete bacteria and red represents incomplete bacteria. Scale bar: 20 μM.


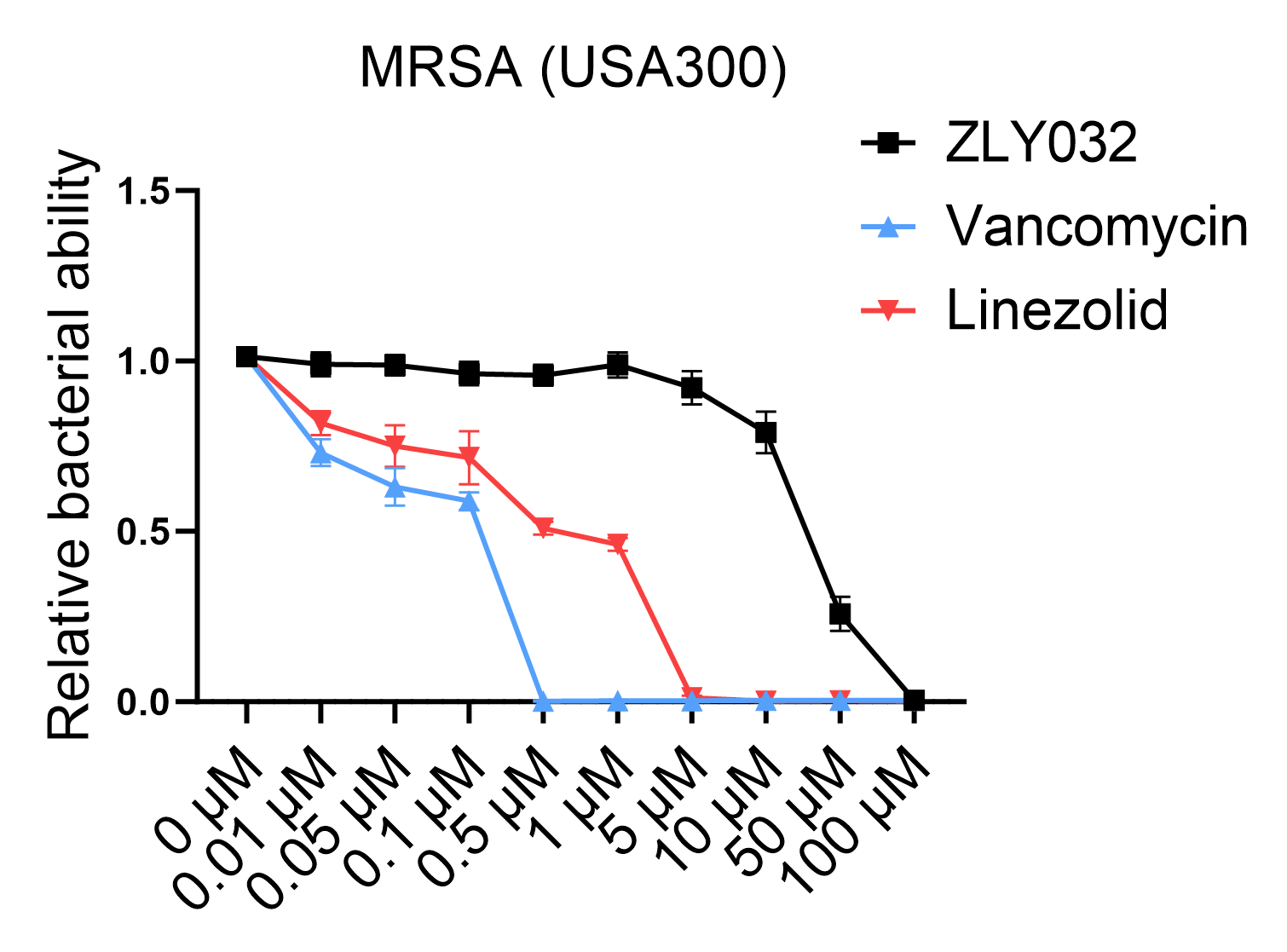


**S25.** The inhibition effects of vancomycin, linezolid and ZLY032 on MRSA (USA300) at the concentration of 0.01 μM, 0.05 μM, 0.1 μM, 0.5 μM, 1 μM, 5 μM, 10 μM, 50 μM and 100 μM. n=6.


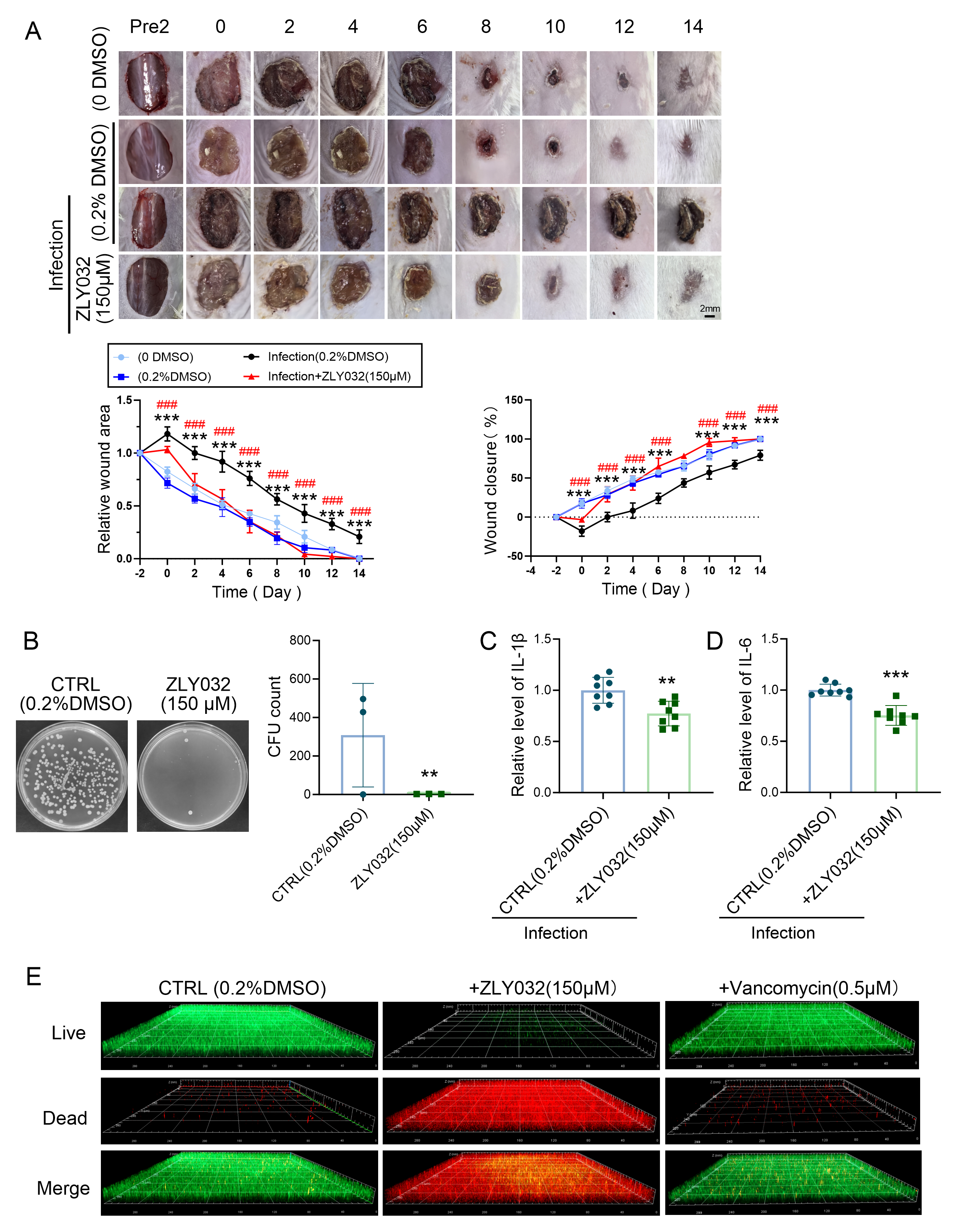


**S26.** **The inhibition effects of ZLY032 in promoting MRSA infected wound healing at MBC. (A)** Up panel: representative photographs showing the time-dependent closure of wounds in MRSA infected mice and the wound healing-promoting effect of ZLY032 at MBC (150μM). Down panel: the wound area and wound closure rate of each mice at varying time points. ****p*<0.001 *vs.* CTRL (0.2%DMSO); *^###^p*<0.001 vs. Infection (0.2%DMSO); n=6 for each group. (Mean ± SD; analysis of variance—ANOVA followed by Dunnett’s test for comparisons among multiple groups). (Mean ± SD; two-way ANOVA followed by Tukey's multiple comparisons test among multiple groups). **(B)** Representative images of bacterial colony in wound fluid in the wound tissue after treating by ZLY032 (150μM) on day 3,***p*<0.01 *vs.* CTRL (0.2%DMSO) ; n=3 for each group. (Mean ± SD; Student *t*-test for comparisons between two groups). **(C-D)** ELISA detection of IL-6 and IL-1β expression in infected wound tissues after ZLY032 treatment (150μM). ***p*<0.01 ****p*<0.001 *vs.* CTRL (0.2%DMSO) ; n=8 for each group. (Mean ± SD; Student *t*-test for comparisons between two groups). **(E)** Live/Dead staining and confocal laser scanning microscopy with 3D reconstruction, green fluorescence (viable MRSA) in upper layers, minimal red fluorescence (dead MRSA) in lower strata. In the ZLY032 (150 μM) treated group, there was marked reduction in green fluorescence and significant increase in red fluorescence. (Mean ± SD; two-way ANOVA followed by Tukey's multiple comparisons test among multiple groups and student *t*-test for comparisons between two groups).


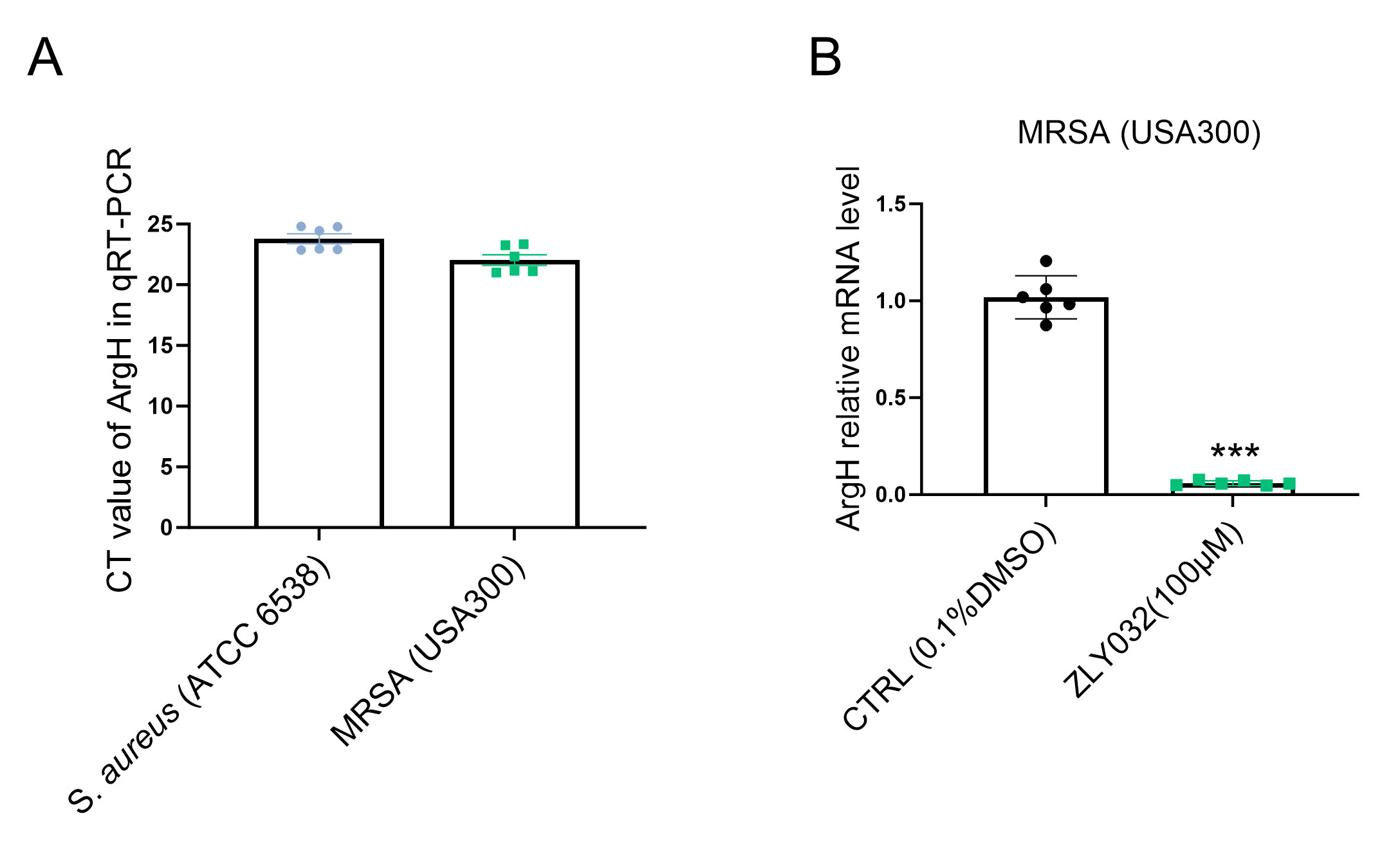


**S27. The expression of ArgH in S aureus (ATCC6538) and MRSA (USA300)**. **(A)** The CT value of ArgH from qRT-PCR in S aureus (ATCC6538) and MRSA (USA300). n=6. **(B)** The effects of ZLY032 on the expression level of ArgH in MRSA (USA300). ****p*<0.001 vs. CTRL(0.1%DMSO), n=6. (Mean ± SD; Student *t*-test for comparisons between two groups).


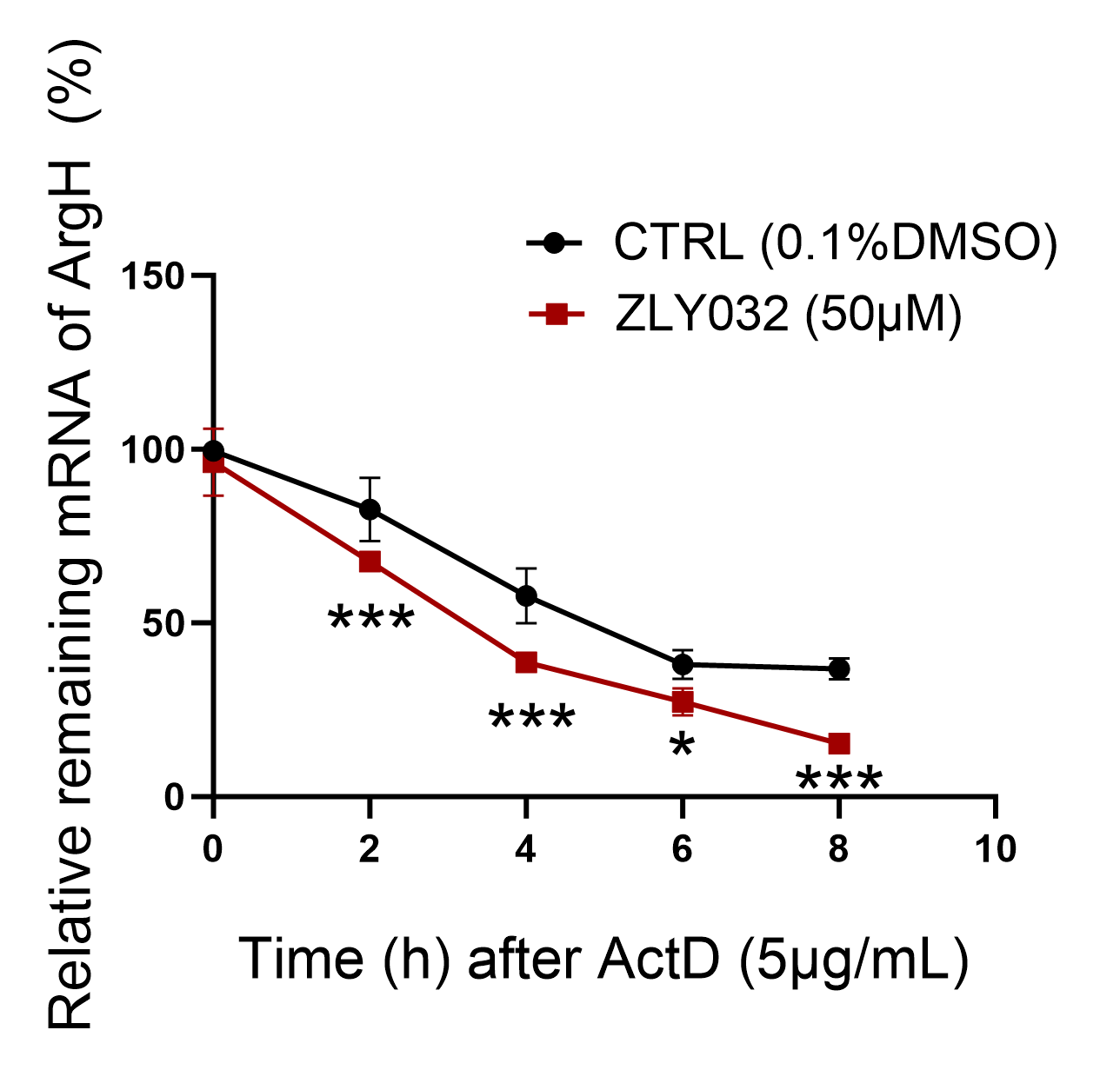


**S28.** **The effect of ZLY032 on the mRNA stability of argH.** ZLY032 significantly inhibited the RNA stability of argH (from S aureus) in actinomycetin D (ActD)-treated 293 cells from 2h, 4h, 6h and 8h, compared with the control group. **p*<0.05, ****p*<0.001 *vs.* CTRL(0.1%DMSO), n=6. (Mean ± SD; Student *t*-test for comparisons between two groups).


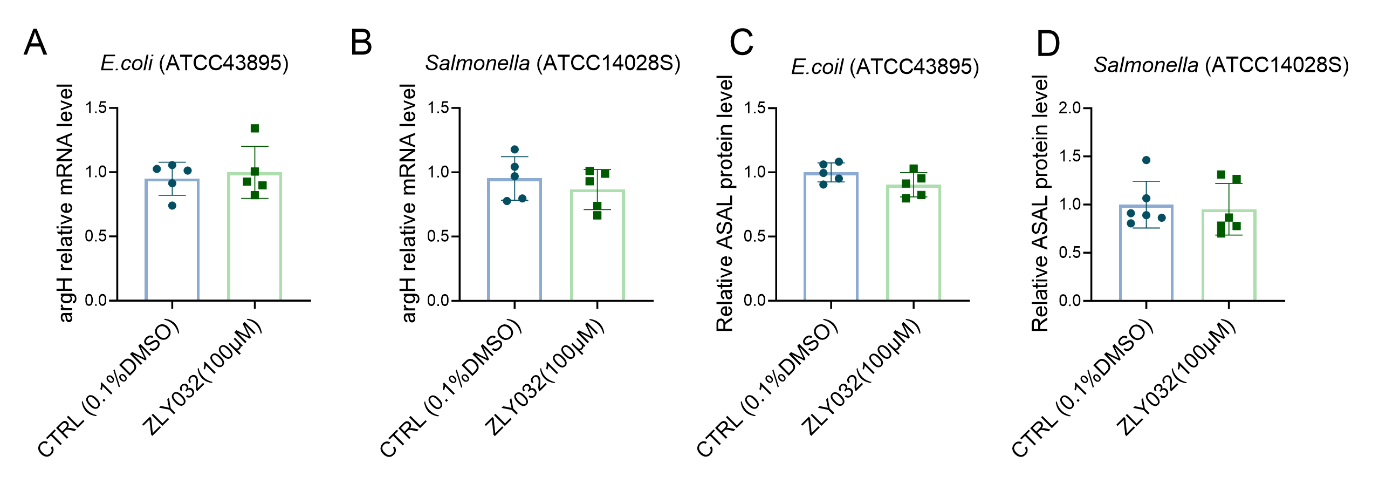


**S29. The effects of ZLY032 on the expression of ArgH and ASAL in *E.coli* and *Salmonella*. (A-B)** qRT-PCR to detect the mRNA expression level of ArgH in *E.coli* and Salmonella. n=5. **(C-D)** The expression level of ASAL in *E.coli* and *Salmonella* was detected by ELISA assay. n=5-6.(Mean ± SD; Student *t*-test for comparisons between two groups).


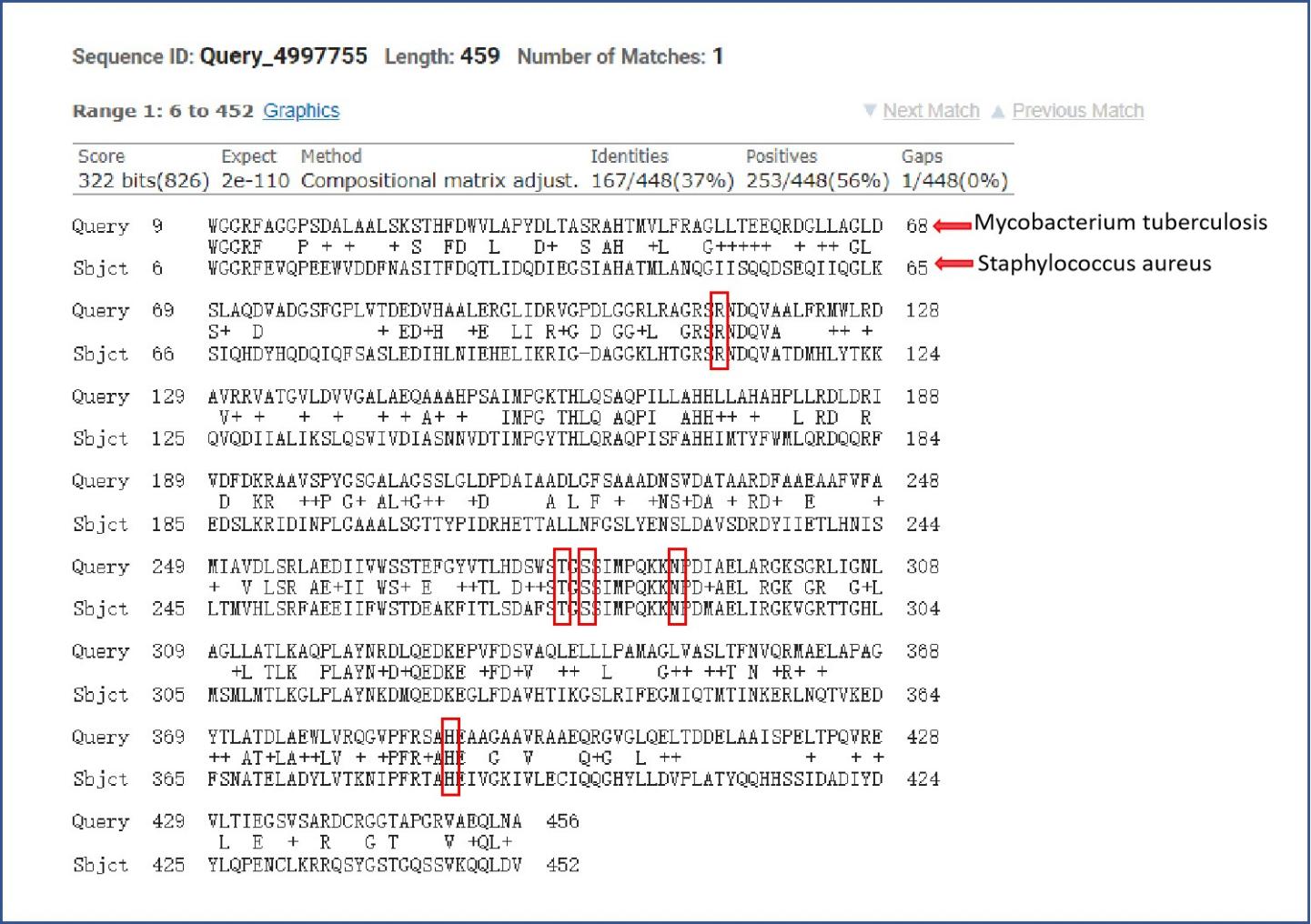


S30. Amino acid sequence blasting of ASAL between mycobacterium tuberculosis and staphylococcus aureus. The red boxes represent conserved binding sites between Mycobacterium tuberculosis and Staphylococcus aureus.


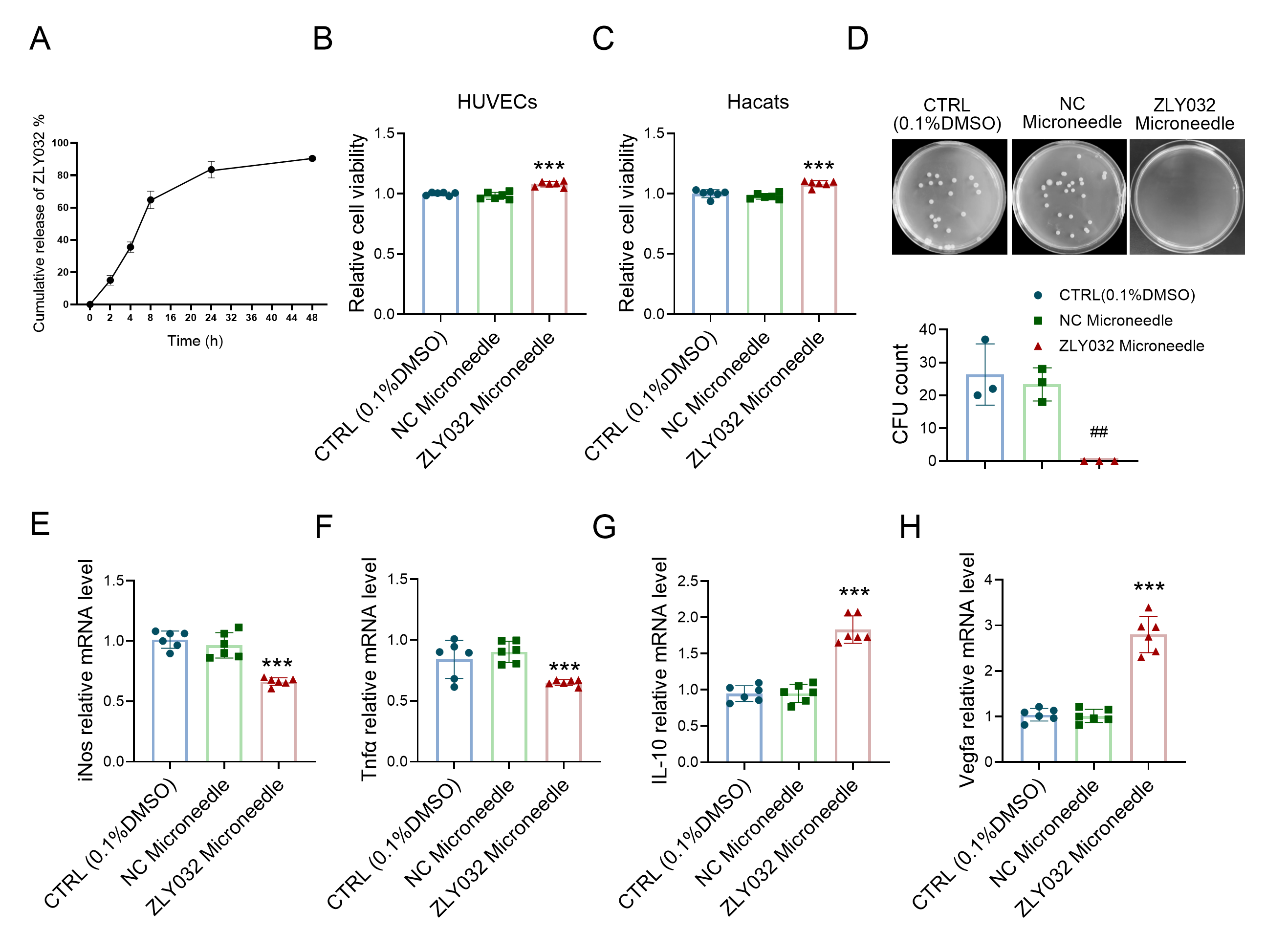


S31. Safety assessment of microneedles. (A) LC-MS quantification of cumulative ZLY032 release from drug-loaded microneedles. (B-C) CCK-8 assay for the effect of drug-loaded or blank microneedles on proliferation of HUVECs and HaCaT cells. ****p*<0.001 *vs.* NC Microneedle, n=8 for each group. (Mean ± SD; ordinary one- way ANOVA followed by Tukey's multiple comparisons test among multiple groups).(D) Representative images of bacterial colony in the wound tissue after treating by drug-loaded or blank microneedles.*^##^p*<0.01 *vs.* NC Microneedle ; n=3 for each group. (Mean ± SD; ordinary one- way ANOVA followed by Tukey's multiple comparisons test among multiple groups) (E-H) The expression level of iNOS, Tnfα, IL-10 and Vegfa in wound tissues treated with drug-loaded versus blank microneedles evaluated by qRT-PCR. ****p*<0.001 *vs.* NC Microneedle, n=6 for each group. (Mean ± SD; ordinary one- way ANOVA followed by Tukey's multiple comparisons test among multiple groups).


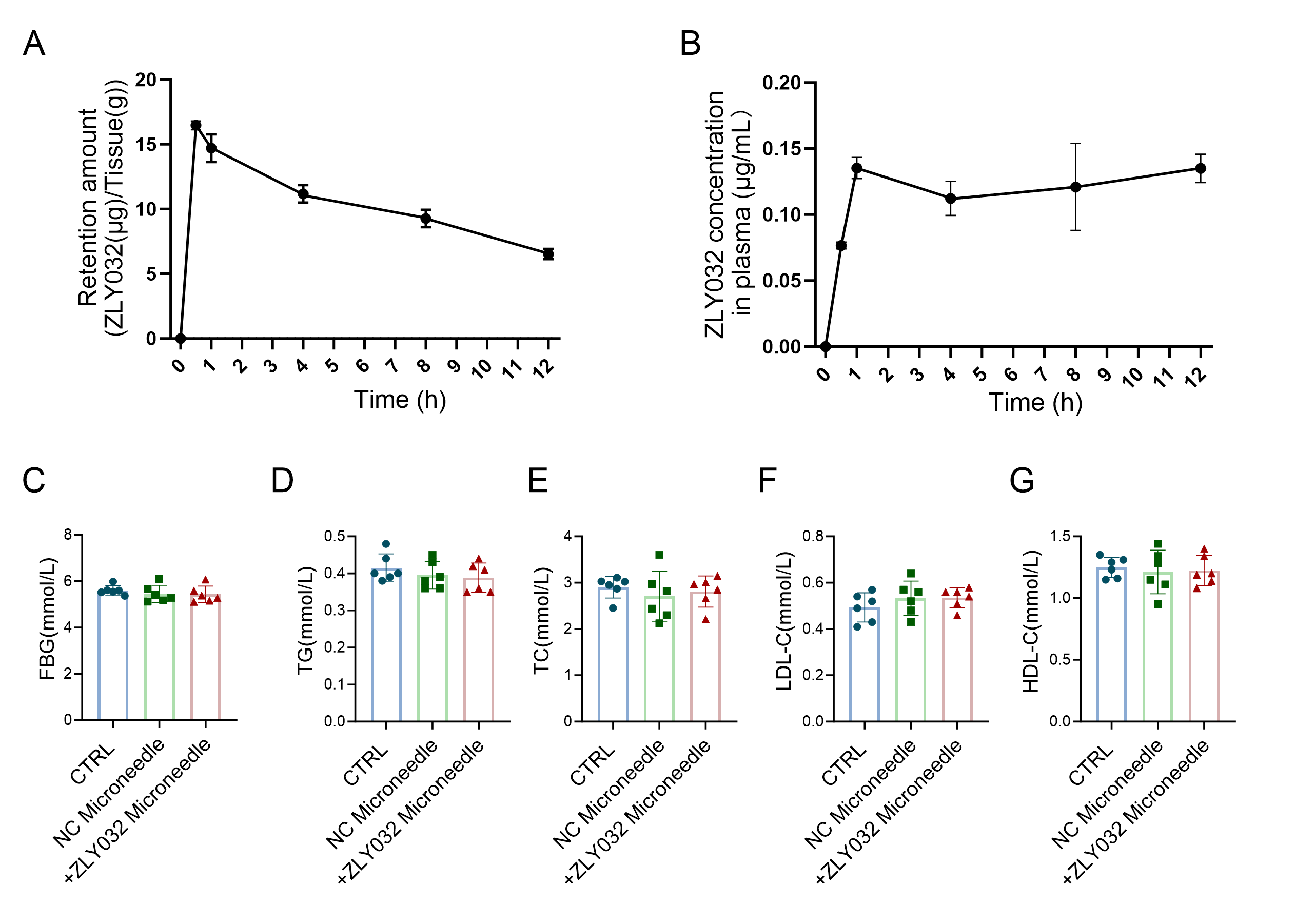


**S32.** The in vivo drug retention of ZLY032-loaded microneedles and its impact on glucose and lipid metabolism. **(A-B)** LC-MS to detect the content of ZLY032 in the local tissues and the circulating blood after the microneedle administered to the wound model mice at specified timepoints (0, 0.5, 1, 4, 8, 12 post-administration). **(C-G)** The effects of ZLY032 loaded microneedle (applied for 0.5h) on the level of fasting blood glucose (FBG), Triglycerides (TG), Total Cholesterol (TC), Low-Density Lipoprotein Cholesterol (LDL-C) and High-Density Lipoprotein Cholesterol (HDL-C) in the plasma of mice. (Mean ± SD; ordinary one- way ANOVA followed by Tukey's multiple comparisons test among multiple groups).

**S33. The synthesis procedure of compound ZLY032.**


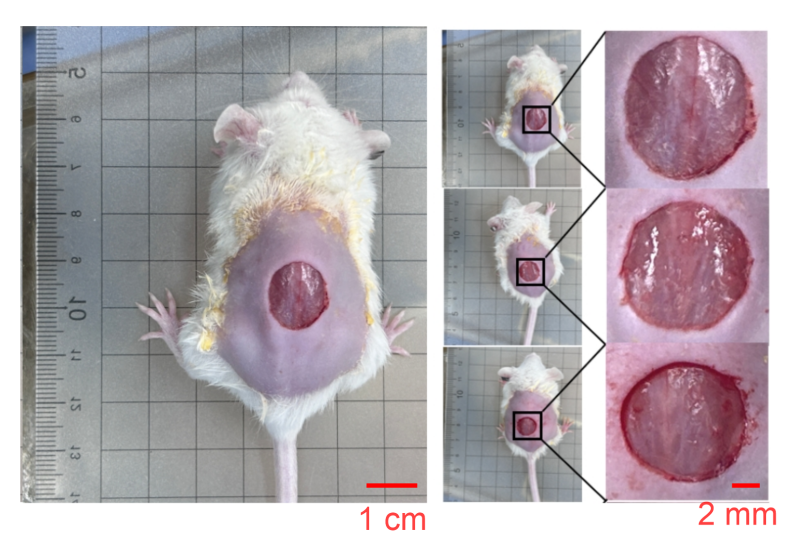


**S34.** Left: Mouse wound image collection; Right: A partial magnification of the wound. Scale：2mm.

| Day | Group | 0.5h | 1h | 2h | 4h | 8h |
| --- | --- | --- | --- | --- | --- | --- |
| DAY0 | ZLY032  (100 μM)-1 | <LLOQ | <LLOQ | <LLOQ | <LLOQ | <LLOQ |
| DAY0 | ZLY032  (100 μM)-2 | <LLOQ | <LLOQ | <LLOQ | <LLOQ | <LLOQ |
| DAY0 | ZLY032  (100 μM)-3 | <LLOQ | <LLOQ | <LLOQ | <LLOQ | <LLOQ |
| DAY3 | ZLY032  (100 μM)-1 | <LLOQ | <LLOQ | <LLOQ | <LLOQ | <LLOQ |
| DAY3 | ZLY032  (100 μM)-2 | <LLOQ | <LLOQ | <LLOQ | <LLOQ | <LLOQ |
| DAY3 | ZLY032  (100 μM)-3 | <LLOQ | <LLOQ | <LLOQ | <LLOQ | <LLOQ |
| DAY14 | ZLY032  (100 μM)-1 | <LLOQ | <LLOQ | <LLOQ | <LLOQ | <LLOQ |
| DAY14 | ZLY032  (100 μM)-2 | <LLOQ | <LLOQ | <LLOQ | <LLOQ | <LLOQ |
| DAY14 | ZLY032  (100 μM)-3 | <LLOQ | <LLOQ | <LLOQ | <LLOQ | <LLOQ |

**Table 1. LC-MS to detect the level of ZLY032 in blood of wound model mice.** LLOQ: Lower Limit Of Quantitation.

| Target | Primer sequence |
| --- | --- |
| iNOS | Forward: CTTGGAGCGAGTTGTGGATTGTC |
|  | Reverse:TCGTAATGTCCAGGAAGTAGGTGAG |
| Arg1 | Forward: AAGACAGCAGAGGAGGTGAAGAG |
|  | Reverse: TAGTCAGTCCCTGGCTTATGGTTAC |
| Tnfα | Forward: ACGTGGAACTGGCAGAAGAGG |
|  | Reverse: TGAGAAGAGGCTGAGACATAGGC |
| Tgfβ1 | Forward: GCAACAATTCCTGGCGATACCTC |
|  | Reverse: GTATTCCGTCTCCTTGGTTCAGC |
| Il-1β | Forward: CAAATCTCGCAGCAGCACATCAAC |
|  | Reverse: AGGTCCACGGGAAAGACACAGG |
| Cd206 | Forward: CTCGGGACTCTGGATTGGACTC |
|  | Reverse: TGATGATGGACTTCCTGGTAGCC |
| Fizz1 | Forward: TGGGTGTGCTTGTGGCTTTG |
|  | Reverse: CAGTGGTCCAGTCAACGAGTAAG |
| Adipor1 | Forward: ACCACCTATGCCCTCCTTTCG |
|  | Reverse: GATGTGTCCAGATGTTGCCAGTC |
| Fgf | Forward: ATGCTTCCACCTCGTCTGTCTAG |
|  | Reverse: TTCACACTCGTAGCCGTTTGC |
| Igf1r | Forward: TGAGAGCAGTGACTCGGATGG |
|  | Reverse: CTGTTGCGGATGAAGCCTGAG |
| Il-10 | Forward:GGACAACATACTGCTAACCGACTCC |
|  | Reverse: TTTCCGATAAGGCTTGGCAACCC |
| Vegfa | Forward:GCAGACCAAAGAAAGACAGAACAAAG |
|  | Reverse: AGTGAACGCTCCAGGATTTAAACC |
| argH | Forward: GATGCCGATATTTACGATTATTTGC |
|  | Reverse: GTTTGACCGATGATTGACCTGTT |
| rpoB | Forward: TGCGAACATGCAACGTCAAG |
|  | Reverse: GCTGTAATAGCCGCACCAGA |
| arcA | Forward: CAATGGAATGATGGCTCAAATACAT |
|  | Reverse: CGTACCAACTCGCTACCAGA |
| arcD | Forward: CGCTGAGAACCAATATCCCCA |
|  | Reverse: TTAACCAATGAACGGCCGGA |
| sdaA | Forward: CATGACCAGTGTAGCCCGTT |
|  | Reverse: TGCGAGCGCAAATGAAACAA |
| ureC | Forward: AGACTTCGGCGTTGATGGTT |
|  | Reverse: TGCACTCTAGGACCATCCGT |
| argB | Forward: TCAAACCAGCAAATCGAGCC |
|  | Reverse: GCAGAACATTGGTGCTGGTT |
| argC | Forward: CTGGCTGTTTCCCTACAGCA |
|  | Reverse: ACGCCGGTCTTAGCATCAAT |
| argF | Forward: TTTGAAGTTGCAGCGCATGA |
|  | Reverse: CCAAGCACACGTGCAGTATC |
| argJ | Forward: GCGAAGGCGCAACAAAGTTA |
|  | Reverse: ACCAAAATTGGCATCTTCGCC |
| arcC | Forward: TGGTAATGGACCACAGGTTGG |
|  | Reverse: TTGCGCCACATTCAGCAAAT |
| argH of Escherichia coli | Forward: CTGGTTGGACTGCCTGCATA |
|  | Reverse: GTTCGCGTAACCCTGTTGC |
| rpoB of Escherichia coli | Forward: CGTGGTGGTGTCGTTCAGTA |
|  | Reverse: AGAGACACACACGGCATCTG |
| argH of Salmon ellaenteritidis | Forward: CGTATGTCGAAATGCTGGCG |
|  | Reverse: CCAGCCTGCCAATTGTTCAC |
| rpoB of Salmon ellaenteritidis | Forward: ATGCCATGTGTGTCTCTGGG |
|  | Reverse: TCTTCCTGGACAACACGCTC |
| Actin | Forward: GCCCAGAGCAAGCGAGGTATC |
|  | Reverse: TTGTAGAAGGTGTGGTGCCAGATC |
| PPARδ(Mouse) | Forward:CTTCAGCAGCCTCTTCCTCAATG |
|  | Reverse: CCAGCAGCCCGTCTTTGTTG |
| PPARδ(Human) | Forward:CGTTGTGGCAAGCAGAGAAGG |
|  | Reverse: CGTTGAGGAAGAGGTTGCTGAAG |
| FFA1(Mouse) | Forward: GGCATCAACATACCCGTGAATGG |
|  | Reverse: GGCAGAAAGAAGAGCAGAATGGAG |
| FFA1(Human) | Forward:GAGTTCAAGACCAGCCTAGCCAAC |
|  | Reverse: TGCCTCAGCCTCCCAAGTAGC |

**Table 2.** Primer sequence.

| Target | siRNA sequence |
| --- | --- |
| PPARδ(Mouse) | GUGACAUCAUUGAGCCCAATT |
|  | UUGGGCUCAAUGAUGUCACTT |
| PPARδ(Human) | CUACAAUGCCUACCUGAAATT |
|  | UUUCAGGUAGGCAUUGUAGTT |
| FFA1(Mouse) | CAAUGUGGCUAGUUUCAUATT |
|  | UAUGAAACUAGCCACAUUGTT |
| FFA1(Human) | CGCUCAACGUCCUGGCCAUTT |
|  | AUGGCCAGGACGUUGAGCGTT |

**Table 3.** siRNA sequence.
